# Ligand Effects on the Spin Relaxation Dynamics and Coherent Manipulation of Organometallic La(II) Potential Qu*d*its

**DOI:** 10.1021/jacs.3c12827

**Published:** 2024-05-24

**Authors:** Lydia E. Nodaraki, Ana-Maria Ariciu, Daniel N. Huh, Jingjing Liu, Daniel O. T. A. Martins, Fabrizio Ortu, Richard E. P. Winpenny, Nicholas F. Chilton, Eric J. L. McInnes, David P. Mills, William J. Evans, Floriana Tuna

**Affiliations:** † Department of Chemistry, University of Manchester, Manchester M13 9PL, U.K.; ‡ Photon Science Institute, University of Manchester, Manchester M13 9PL, U.K.; § Department of Chemistry, University of California, Irvine, California 92697, United States of America; ∥ Department of Chemistry, University of Rhode Island, Kingston, Rhode Island 02881, United States of America; ⊥ School of Chemistry, University of Leicester, Leicester LE1 7RH, U.K.; # Research School of Chemistry, Australian National University, Canberra 2617, Australia

## Abstract

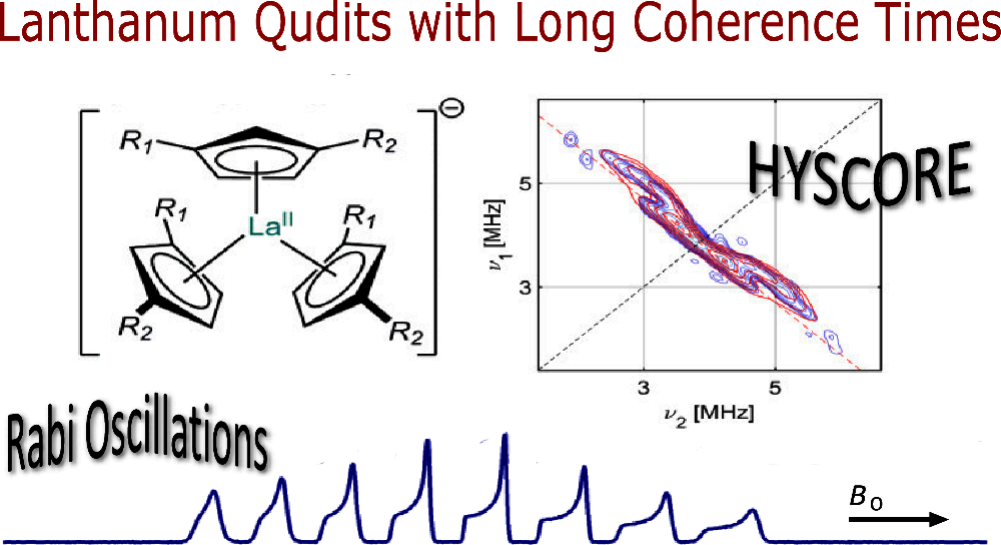

We present pulsed electron paramagnetic resonance (EPR) studies on three La(II) complexes, [K(2.2.2-cryptand)][La(Cp′)_3_] (**1**), [K(2.2.2-cryptand)][La(Cp″)_3_] (**2**), and [K(2.2.2-cryptand)][La(Cp^tt^)_3_] (**3**), which feature cyclopentadienyl derivatives as ligands [Cp′ = C_5_H_4_SiMe_3_; Cp″ = C_5_H_3_(SiMe_3_)_2_; Cp^tt^ = C_5_H_3_(CMe_3_)_2_] and display a *C*_*3*_ symmetry. Long spin–lattice relaxation (*T*_1_) and phase memory (*T*_m_) times are observed for all three compounds, but with significant variation in *T*_1_ among **1**–**3**, with **3** being the slowest relaxing due to higher s-character of the SOMO. The dephasing times can be extended by more than an order of magnitude via dynamical decoupling experiments using a Carr–Purcell–Meiboom–Gill (CPMG) sequence, reaching 161 μs (5 K) for **3**. Coherent spin manipulation is performed by the observation of Rabi quantum oscillations up to 80 K in this nuclear spin-rich environment (^1^H, ^13^C, and ^29^Si). The high nuclear spin of ^139^La (*I* = 7/2), and the ability to coherently manipulate all eight hyperfine transitions, makes these molecules promising candidates for application as qu*d*its (multilevel quantum systems featuring *d* quantum states; *d* >2) for performing quantum operations within a single molecule. Application of HYSCORE techniques allows us to quantify the electron spin density at ligand nuclei and interrogate the role of functional groups to the electron spin relaxation properties.

## Introduction

The chemistry of lanthanide (Ln) ions is largely dominated by the +3 oxidation state regardless of the number of 4f valence electrons because the +3 state constitutes a good balance between lattice and ionization enthalpies.^1^ The 4f^n^ Ln(III) to 4f^n-1^ Ln(II) reduction potentials of six lanthanides, Eu, Yb, Sm, Tm, Dy, and Nd, allow the isolation of compounds containing +2 ions, but Ln(II) complexes for the other lanthanides were not expected to be isolable.^2^ However, recent advances in organometallic chemistry have made stabilization of the +2 oxidation state possible for almost all elements of the 4f series, with tris(cyclopentadienyl) ligand environments being the first to stabilize Ln(II) complexes of all the other lanthanides except radioactive *Pm*.^3−5^ Structural, spectroscopic and density functional theory (DFT) studies have indicated that the trigonal environment generated by three substituted cyclopentadienyl (Cp^R^) ligands significantly stabilizes the (n+1) d_*z*_^2^ orbital, such that a 4f^n^5d^1^ ground state configuration is typically found for the reduced Ln(II) ions,^3−5^ and a 4d^1^ configuration for Y(II).^6^ Complexes involving such unusual Ln(II) oxidation states have gained interest because of their unusual reactivity^5,7^ and electronic structure.^8−10^

One interest is in the electron spin relaxation properties, which has led to interest in a quantum information science (QIS) context. We recently reported the relaxation properties of a tris-cyclopentadienyl yttrium(II) complex.^9^ In common with several [Ln^II^X_3_]^−^ type complexes (X = Cp^R^, amide, aryloxide), the *pseudo*-*C*_*3*_ symmetry enables direct mixing of the metal valence *s*- and *d*_*z*_^2^ atomic orbitals, resulting in a large and near isotropic metal hyperfine interaction. This has the knock-on effect of retarding electron spin *T*_1_ relaxation driven by spin–orbit coupling (which enables exchange of energy between the spin system and the lattice when the electronic energy levels are modulated by molecular motions) since the orbital angular momentum is largely quenched. The longer *T*_1_ then does not limit *T*_m_, the phase memory time, and allows coherent manipulation of the spin to higher temperatures (up to room temperature for the Y(II) example).^9^ This near isotropic nature has some analogies with ^2^*S*_1/2_ state ions such as ^171^Yb^+^ used as hyperfine qubits in ion-trap QIS,^10^ and has been exploited to observe clock transitions (transitions at avoided level crossings where relaxation is prolonged because the transition frequency becomes insensitive to magnetic field fluctuations) at X-band microwave frequencies.^11^ The combination of such strategies with other approaches that exploit, for example, atomic clock transitions,^12,13^ removal of nuclear spins,^14,15^ or reduction of vibrational relaxation,^16−18^ could sufficiently enhance molecular qubit properties without the need to apply a too high degree of magnetic dilution. This is particularly important for quantum computation as its implementation demands bringing qubits together and to perform qubit gates (logic operations)^19−22^ to carry out specific algorithms.

Another approach to the latter problem is to increase the dimension of the spin system (the Hilbert space) in individual molecules, rather than bringing multiple *S* = 1/2 qubits together. Such multistate systems are called qu*d*its (*d* is the dimension of the Hilbert space).^19,23^ For an *S* = 1/2 molecule, the dimension of the spin system can be expanded by coupling to a nuclear spin via the hyperfine interaction. Hence, for the Y(II) example above the spin system has a dimension *d* = (2*S*+1) × (2*I*+1) = 4 due to interaction of the electron spin *S* = 1/2 with the ^89^Y *I* = 1/2 nuclear spin, with the opportunity to encode information in the electronuclear states. Such an approach has been proven in molecular systems to implement, for example, Grover’s algorithm in [Tb(phthalocyanine)_2_] (^159^Tb, *I* = 3/2)^24^ and a quantum simulator using [Yb(trensal)] (^173^Yb, *I* = 5/2).^25^

Hence, following our work on [Y(Cp^R^)_3_]^−^ we were keen to study other members of the [Ln^II^(Cp^R^)_3_]^−^ family where the metal possesses a larger nuclear spin, such that several electronuclear transitions can be expected to be accessible for coherent spin manipulations via microwave pulses.^8,9,26−28^

**Figure 1:**
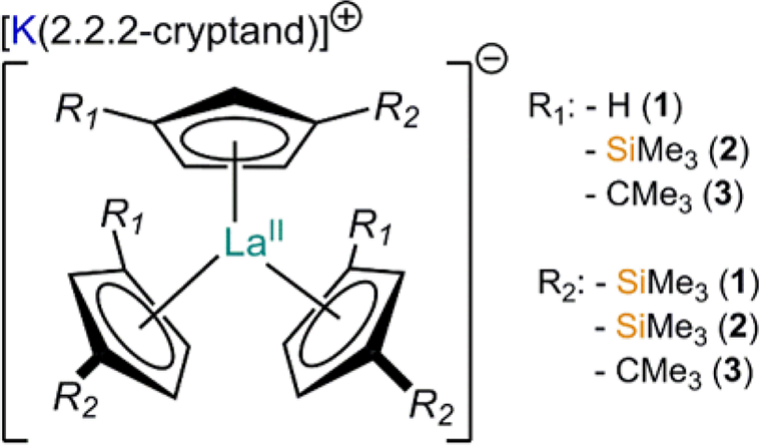
Schematic representation of the structures of **1**, **2** and **3**.

This can be regarded as an alternative route for the realization of multistate systems that open opportunity for larger and more complex calculation algorithms to be conducted within a single molecular unit.^29^ Such qu*d*it systems have been proposed for diminished quantum error rates and simplified quantum logic,^30^ and the potential advantages of molecules in this context have been reviewed recently elsewhere.^31^ Herein, we demonstrate that the nuclear Hilbert space of [Ln(Cp^R^)_3_]^−^ can be expanded through the hyperfine interaction with the nuclear spin of the ^139^La(II) isotope (*I* = 7/2; 99.95% natural abundance), thus leading to qu*d*its with *d* = 16. We report coherent spin manipulations across the multiple electronuclear spin states in the family of complexes, [K(2.2.2-cryptand)][La(Cp′)_3_]^32^ (**1**), [K(2.2.2-cryptand)][La(Cp″)_3_]^3^ (**2**) and [K(2.2.2-cryptand)][La(Cp^tt^)_3_]^33^ (**3**) (Figure 1; Cp′ = C_5_H_4_SiMe_3_; Cp″ = C_5_H_3_(SiMe_3_)_2_; Cp^tt^ = C_5_H_3_(CMe_3_)_2_) that contain one unpaired electron primarily residing in a low-energy s/d_z2_ orbital as described above. The three Cp^R^ ligands feature one or two SiMe_3_ or CMe_3_ functional groups; these have been chosen to allow systematic study of the effect of differing sterics and electron-donating/withdrawing nature of the substituents within the same ligand framework, and hence of spin density distribution between ligand and metal, on the spin dynamics.

## Results and Discussion

### Synthesis and structural characterization

The synthesis and structural characterization of **1**,^32^**2**,^3^ and **3**(33) were performed according to the previously reported methods; their crystal structures are presented in the Supporting Information, Figures S1–S3. In these complexes, La(II) resides at the center of the plane formed by the centroids of the three cyclopentadienyl ligands leading to a nearly trigonal planar arrangement. Complexes **1**–**3** each displays a local *pseudo*-*C*_*3*_ symmetry at the metal center, with the *C*_*3*_ axis passing through the metal, and perpendicular to the plane made by the centroids of the three Cp^R^ rings. The *C*_*3*_ symmetry is important as it dictates the d_*z*_^2^ orbital to be lowest in energy and thus able to accommodate the unpaired electron. The geometric parameters of the anions are very similar for the three different complexes; the mean La–C(Cp^R^) distances are 2.853(9) Å, 2.88(2) Å and 2.90(8) Å for **1**, **2** and **3**, respectively, while the mean La-centroid lengths are 2.586 Å (**1**) 2.620 Å (**2**) and 2.638 Å (**3**), in line with the varying steric effects of ring substituents.

### Electron paramagnetic resonance

**Figure 2:**
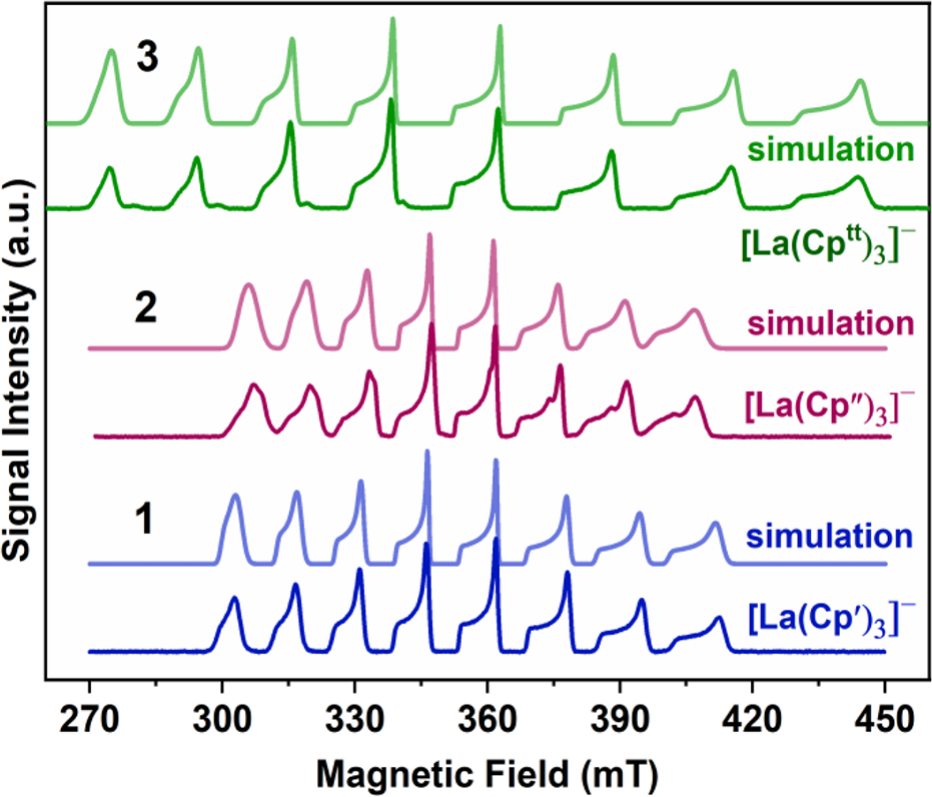
Echo-detected field-swept data of **1** (blue), **2** (purple) and **3** (green) recorded at X-band (9.67 GHz) on frozen MeTHF solutions (see Table 1 for simulation parameters).

Continuous-wave (CW) and echo-detected field-swept (EDFS) EPR spectra for **1**-**3** in MeTHF (Figure 2 and Figures S4–S8) display 8-line patterns due to the hyperfine interaction of the electron with the nuclear spin of ^139^La. Spectra are nicely simulated with the parameters in Table 1, using EasySpin^34^ and the spin Hamiltonian *Ĥ = g*μ_*B*_*BS + IAS*, with *g* and *A* as the axial *g*-tensor and the hyperfine coupling tensor, respectively. Simulations for **2** were further improved by adding a small amount of an unknown La(II) species (Figures S7 and S8). This is not unexpected, as other compounds, [K(dme)_*x*_][La(Cp″)_3_] and [La(Cp″)_2_(dme)_*y*_], were found to coexist in thermal equilibrium in the EPR spectra of THF solutions of [La(Cp″)_3_] undergoing reduction in the presence of K.^35^ The parameters in Table 1 are in line with the values reported in the literature for La(II) complexes.^3,9,35−37^ The pattern of *g*-values is consistent with a 5d_*z*_^2^ ground state with *g*_*z*_ ≈ *g*_e_ > *g*_x,y_, where *g*_e_ is the free-electron *g*-value. The largest *g*-anisotropy is observed for **3**, which is somewhat surprising given the heavier atoms in **1** and **2**.

**Table 1:** Extracted EPR parameters for 1, 2, and 3 (10 mM MeTHF)

**Complex**	*T***(K)**	***g***	*A***(MHz)**	*A***(G)**	*C***(10**^**–7**^**μs**^**–1**^**)**	***n***
**1**	80	*g*_∥_ = 1.999	*A*_∥_ = 420	*A*_∥_ = 150	10.7(4)	2.58(5)
		*g*_⊥_ = 1.956	*A*_⊥_ = 430	*A*_⊥_ = 157		
	295	*g* = 1.994	*A* = 430	*A* = 154^14^		
**2**	80	*g*_∥_ = 2.001	A_∥_ = 392	*A*_∥_ = 140	8.73(2)	2.71(2)
		*g*_⊥_ = 1.950	A_⊥_ = 385	A_⊥_ = 141		
	295	*g* = 1.990	*A* = 372	*A* = 133.5^10^		
**3**	40	*g*_∥_ = 1.998	*A*_∥_ = 650	*A*_∥_ = 232	1.23(7)	3.22(7)
		g_⊥_ = 1.934	*A*_⊥_ = 630	*A*_⊥_ = 233		

Hyperfine interactions can involve two different mechanisms: the Fermi-contact (through bond) interaction and the anisotropic dipolar (through space) coupling.^38^ The metal hyperfine interaction in **1** - **3** is dominated by the isotropic part in each case. This arises from electron spin density at the nucleus and can be related to the 6s-orbital character of the SOMO (which is predominantly 5d_*z*_^2^). The comparable *A*_iso_ values for complexes **1** and **2** (averaged anisotropic values of 426.7 and 391.7 MHz, respectively), that incorporate one and two SiMe_3_ substituents respectively, suggest a similar degree of 6s-5d_*z*_^2^ mixing in the SOMO, while that for **3** is substantially larger. The valence 6s-orbital spin population can be estimated by dividing *A*_iso_ by the atomic isotopic hyperfine constant, here *A* = 6007 MHz for ^139^La.^39^ The results indicate that the SOMO has approximately 7.1% and 6.5% 6s character in **1** and **2**, respectively, which is consistent with previous DFT calculations on [Ln(Cp′)_3_]^−^ (Ln = Y, La).^9^ Complex **3** shows a larger *A*_iso_ (636.7 MHz), from which we derive a 6s-spin population of 10.6%, indicating a higher degree of 6s-5d_*z*_^2^ mixing relative to **1** and **2**. Hence, replacement of SiMe_3_ in **2** with CMe_3_ in **3** leads to a substantial increase in s-electron density at the La, i.e. the electronic properties of the ligands are a major factor in the orbital admixing at the metal ion. This finding is significant because the spin relaxation properties of the compounds relate to the population of the SOMO, with a more pronounced s-character of SOMO leading to prolonged electron spin relaxation.^8^

**Figure 3:**
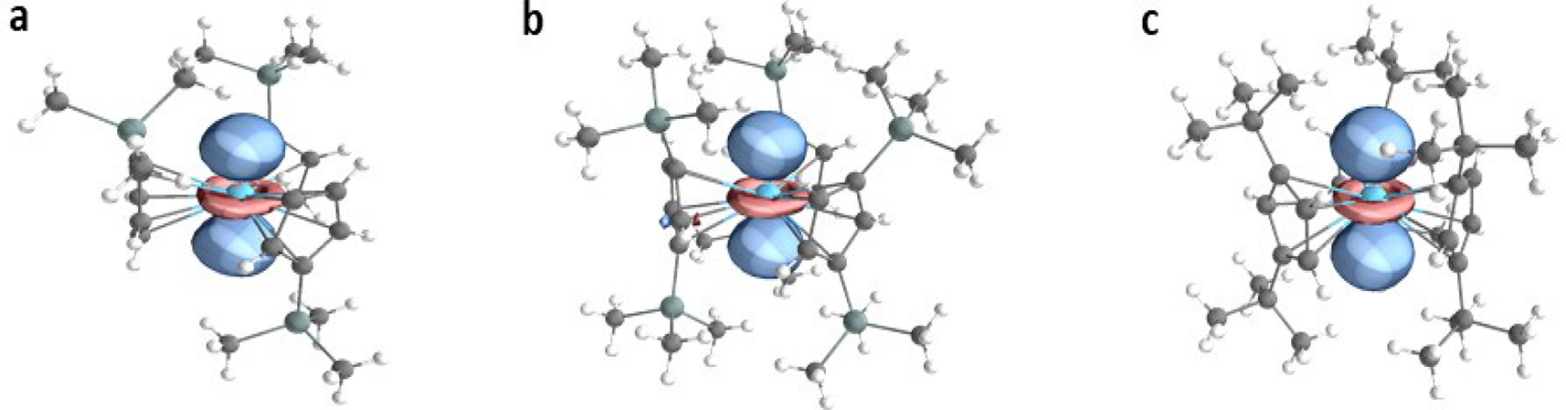
Renderings of the SOMO for (a) **1**; (b) **2;** and (c) **3,** from DFT calculations on the crystal structures of the anions.

DFT calculations on the crystal structures of the anions in **1**–**3** provide full support for these findings, with *A*_iso_ values of 390 MHz for **1**, 360 MHz for **2**, and 641 MHz for **3** (Table S13), also reproducing the experimental trend **3** ≫ **1** > **2** and the minimal hyperfine anisotropy. Löwdin population analysis of the SOMO (Figure 3) in each case gives 7.7, 7.8 and 10.0% s-character for **1**-**3**, respectively, in agreement with the experimentally derived values above, and reports 59, 59 and 61% d-character, respectively (Table S14). Löwdin analysis of the spin density places 0.71 electron spin density on the La site in **1** and **2**, but 0.76 spin density on the La site in **3**, and hence ca. 30% of the electron spin density is associated with the ligand scaffold.

### Spin–lattice relaxation and spin coherence times

**Figure 4:**
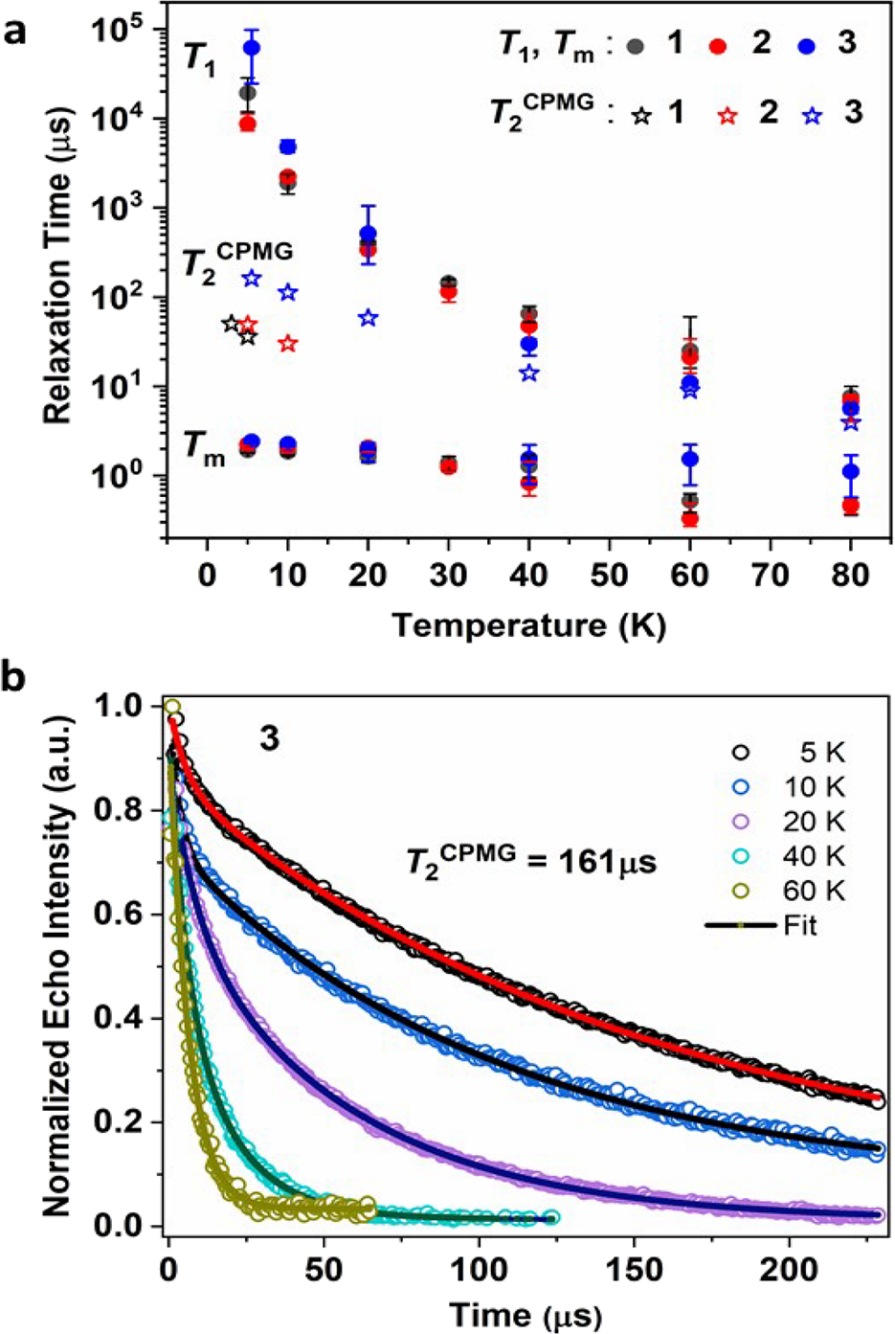
(a) Temperature dependence of *T*_1_, *T*_2_ and *T*_2_^CPMG^ for **1**–**3**. (b) Decay of the echo intensity for **3** at temperatures between 5 and 60 K, measured at 366.2 mT with a CPMG sequence. Solid lines are exponential fits (parameters in Table S9).

Observation of well resolved EDFS spectra up to ca. 100 K in frozen MeTHF solutions of **1**–**3** encouraged us to measure relaxation times, as rare earth complexes rarely show spectra at such a high temperature. The spin–lattice relaxation time constants (*T*_1_) at different temperatures were derived by fitting inversion recovery data (π–t−π/2−τ–π–τ–*echo*, with variable *t*)^40^ to a biexponential function (Figures 4a, Figures S9–S17, and Tables S1–S3). *T*_1_ is strongly temperature dependent down to 5 K and reaches values of *ca*. 17, 9, and 50 ms for **1**, **2** and **3**, respectively, measured on peaks corresponding to x,y orientations (*B*_0_ ⊥ C_3_). There is significant variation across measurement positions, with longer relaxation times found for the *z*-orientation (*B*_0_ ∥ C_3_), reaching as long as 98 ms for **3** (Tables S1–S3). Fitting of the data assuming a Raman-like dependence of *T*_1_ with temperature (*CT*^*n*^)^9,27^ gave the Raman parameters in Table 1 (Figures S23, S27, and S30). The coefficient *C* is noticeably lower for **3**. It is tempting to ascribe the faster *T*_1_ relaxation for **1** and **2** than for **3** as being due to enhanced spin–orbit coupling (SOC) due to the heavier nuclei (Si) in the ligand set.^41^ However, as noted above, the *g*-anisotropy, also dependent on SOC, is also greatest for complex **3**. Hence, the slower relaxation of **3** must be due to another factor, and a likely candidate is the significant difference in electron spin distribution as evident from the metal hyperfine interaction. The *T*_1_ times correlate with the isotropic metal hyperfine, and hence with the metal valence orbital *s*-character of the SOMO, with **3** ≫ **1** > **2**. Other possible factors affecting the relaxation times are the interactions of the electron with the environmental nuclear spins.^42,43^ Rotations of Me groups in particular cause spin relaxation through a mechanism known as spectral diffusion (SD).^42^ Fitting of the inversion recovery data of **1**–**3** included a temperature-dependent term associated with spectral diffusion (SD).^19^ The obtained *T*_SD_ values are significantly smaller than *T*_1_ and follows the variation in *T*_1_, *i.e*. *T*_SD_ decreases as the temperature is raised, and is shortest for **2** (Tables S1–S3). This is consistent with spectral diffusion effects, and the steric demands of the Cp^R^ ligands. SiMe_3_ substituents in **2** are less hindered than CMe_3_ in **3,** because of longer C(Cp)-Si bonds, leading to larger separations between substituents and Cp rings in **2** than **3**. Thus, Me groups in **2** are likely more efficient in triggering relaxation by spectral diffusion mechanism.^42^

Phase memory time constants (*T*_m_), measured with a Hahn echo decay sequence,^39^ are temperature independent below *ca*. 10 K, and reach values of 2.0, 2.2, and 2.4 μs for **1**, **2** and **3**, respectively, at 5 K (Figures S18–S30 and Tables S4–S6). Similar *T*_m_ values have been observed for related complexes^9^ which, in common with **1**-**3**, give electron spin echoes up to relatively high temperatures despite the ^1^H and Me-group rich environment (and without optimization of concentration). In that work it was argued that *T*_m_ was limited by nuclear spin diffusion, thought that spectral diffusion effects cannot be excluded.

The effect of such processes on relaxation can be suppressed by dynamical decoupling experiments using the Carr-Purcell-Meiboom-Gull (CPMG) pulse sequence,^44^ which involves an initial π/2 followed by a series of π pulses. Indeed, such experiments on **1**-**3** gave much longer time constants than two-pulse Hahn echo experiments (Figure 4, Figures S31–S36, and Tables S7–S9). For **1**, **2** and **3**, respectively, we find CPMG time constants as high as 36, 49, and 161 μs at 5 K, with the higher values observed for z orientations. The reason for the much greater enhancement for **3** than for **1** and **2** is not immediately clear.

### Coherent manipulation of spins by microwave pulses

Coherent manipulation of the electron spin in **1**–**3** was studied by transient nutation experiments, using a 3-pulse sequence that involves an incremented tipping pulse, *t*_p_, followed by Hahn echo detection.^27,45^ This corresponds to creation and measurement of arbitrary superposition states. The oscillation in the echo intensity as a function of the duration of the initial pulse is known as a Rabi oscillation.^46^

Rabi oscillations were detected for all eight hyperfine transitions of the three complexes (Figure 5a and Figures S36–S45,S48–S56 and S59–S62) up to the temperature of 80 K. Fourier transform analysis (Figure 5b and Figures S36–S45, S48–S56,and S59–S62) reveals a clear linear dependence of the oscillation frequency with the microwave field strength, *B*_1_ (Figure 5c and Figures S46, S57, and S63). This confirms their origin as Rabi oscillations with Rabi frequency Ω_R_. There is also a sharp *B*_1_-independent peak observed, which corresponds to the ^1^H Larmor frequency,^27^ due to interactions with ^1^H nuclei on ligands and/or solvent.

**Figure 5:**
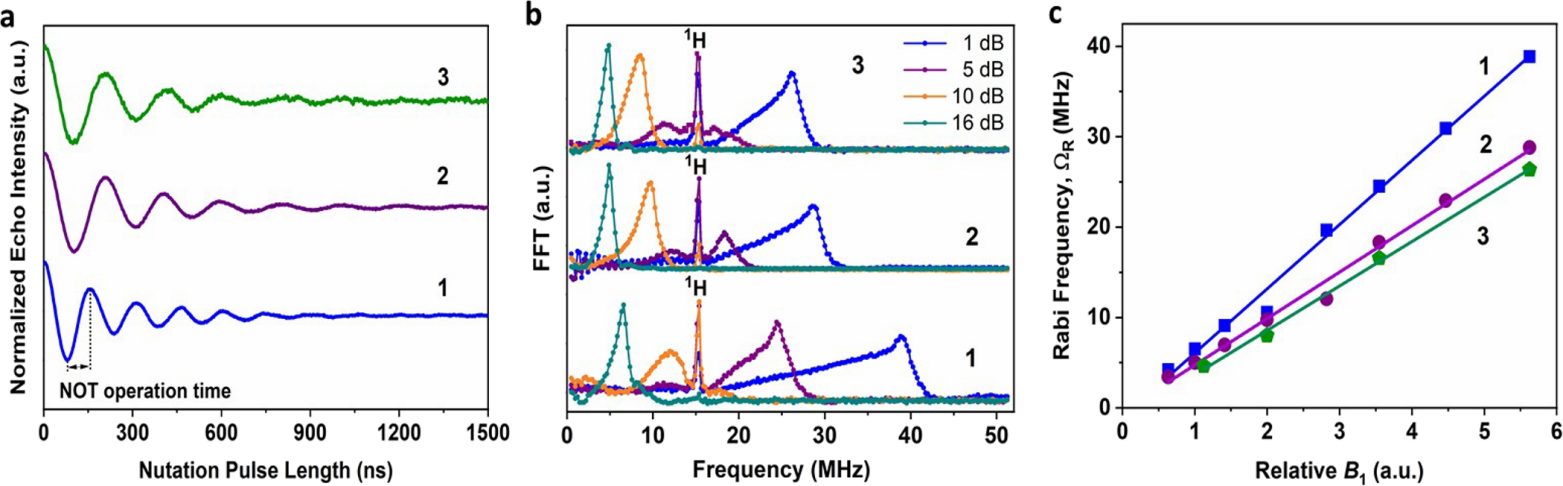
(a) Nutation (Rabi oscillations) data for **1**, **2** and **3** measured at 16 dB at 20 K; (b) Fourier transforms of the nutation data at different microwave powers; and (c) *B*_1_ dependence of the Rabi frequency (Ω_R_); the solid line is a guide of the eye emphasizing the linear dependence.

The nutation data provide information on the properties of **1**-**3** as potential qubits. The time period between a maximum and adjacent minimum of the oscillation corresponds to the flipping time of the spin, meaning the time required for executing a logical operation.^47^ Crucially, the operation time parameter needs to be notably shorter than the lifetime of the qubit in order the qubit to be functional. This operation time is 78, 115, and 105 ns for **1**, **2** and **3**, respectively, from the nutation data recorded under 16 dB applied mw power (Figure 5a). This time reduces significantly for higher power pulses. The qubit figure of merit, Q_M_, is defined as 2Ω_R_T_2_ and represents the number of coherent single–qubit NOT computational operations.^48^ For complexes **1**, **2** and **3** the Q_M_ value is 156, 128 and 130, respectively. These values are significant for rare earth qubits,^26^ and comparable to the Q_M_ values of other molecular systems.^49^ With *T*_2_^CPMG^ = 161 μs (**3**), we predict unprecedented Q_M_ = 8720 for lanthanide complexes. The fact that such manipulations can be performed at 80 K on all eight hyperfine transitions indicates that this system has robust quantum properties and great ability to form a qu*d*it with *d* = 16.

### HYSCORE spectroscopy

As noted above, stabilization of the +2 oxidation state of the lanthanum ion implies an electronic configuration change from 4f^0^5d^0^ to 4f^0^5d^1^ upon reduction of La(III) to La(II).^3,32,33^ However, the assumption that the unpaired electron of La(II) purely resides in the lowest energy 5d_*z*_^2^ orbital is inconsistent with the small anisotropy of the *g*- and hyperfine *A*- tensors. This implies significant electron spin density on the ligands. In the context of QIP, weak electron–nuclear interactions can contribute to decoherence, shortening qubit lifetime.^28^ Given the CPMG results, and observation of ^1^H interaction in the nutation experiments, we probed the weak ligand hyperfine interactions in **1**, **2** and **3** by using the two-dimensional hyperfine sublevel correlation (HYSCORE) technique.^50^ This hyperfine method allows quantifying small (down to sub-MHz) hyperfine couplings to nuclear spins such as ^1^H, ^13^C or ^29^Si.^51,52^

HYSCORE uses a four-pulse electron spin–echo sequence (π/2 - τ - π/2 - *t*_1_ - π - *t*_2_ - π/2 - τ – *echo*, with fixed τ and variable *t*_1_ and *t*_2_), which creates correlations between the nuclear frequencies in the α and β electron spin manifolds in a 2D experiment. For each of **1**–**3** we observe signals centered on the ^1^H and ^13^C Larmor frequencies of the HYSCORE spectra (Figure 6 and Figures S64–S74).

**Figure 6:**
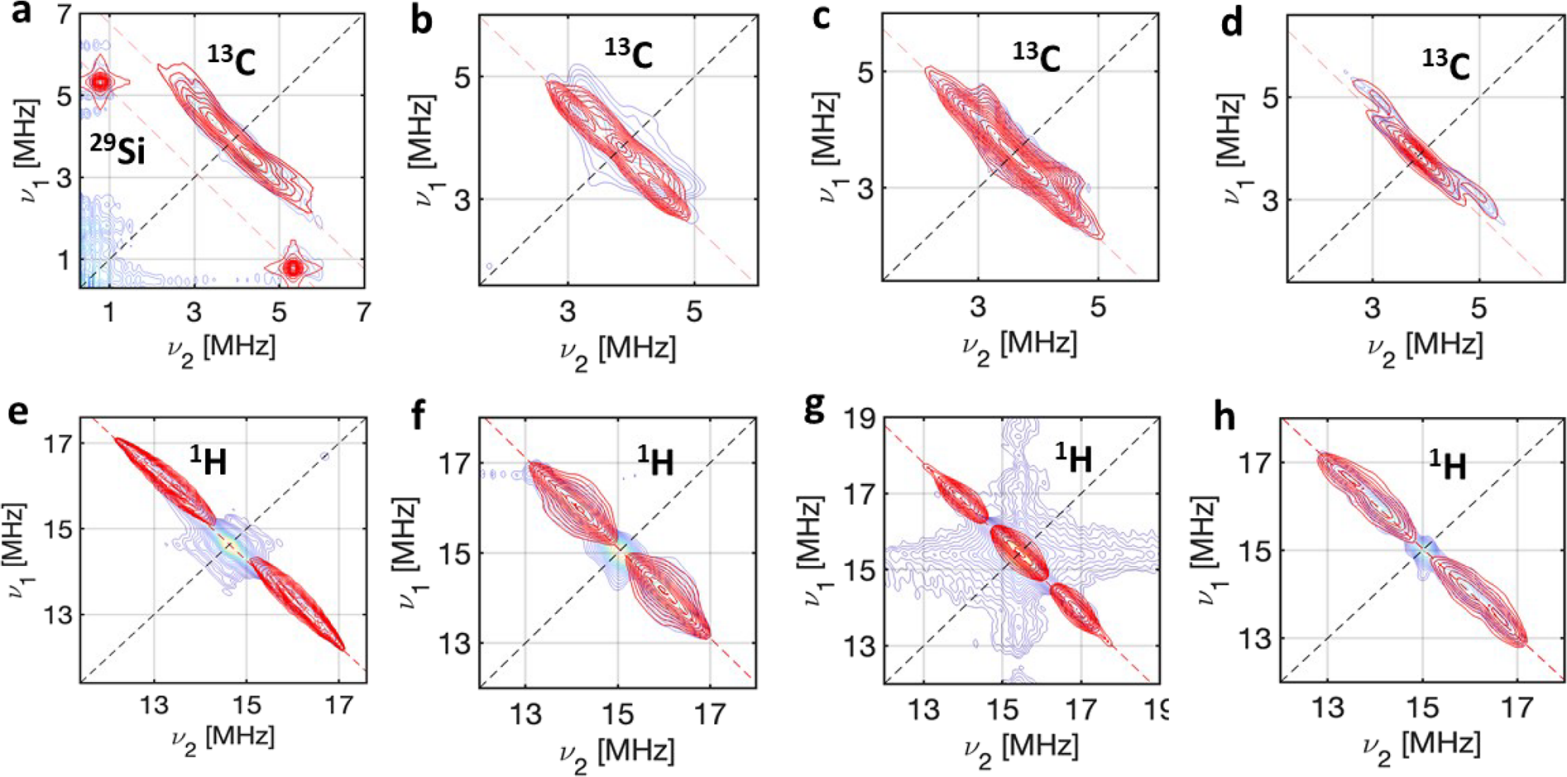
X-band HYSORE spectra: (a) ^13^C and ^29^Si region for **1** at a static field of *B*_0_ = 360.8 mT (at *g*_*xy*_); (b) ^13^C region for **1** at *B*_0_ = 353.8 mT (at *g*_*z*_); (c) ^13^C region for **2** at *B*_0_ = 333.3 mT (at *g*_*xy*_); (d) ^13^C region for **3** at *B*_0_ = 366.2 mT (at *g*_*xy*_); (e) ^1^H region for **1** at a static field of *B*_0_ = 345.3 mT (at *g*_*xy*_); (f) ^1^H region for **1** at a static field of *B*_0_ = 353.8 mT (at *g*_*z*_); (g) ^1^H region for **2** at a static field of *B*_0_ = 361.7 mT (at *g*_*xy*_); (h) ^1^H region for **2** at a static field of *B*_0_ = 352.8 mT (at g_*z*_), with the simulation in red based on the model described in the text.

To model the spectra we have taken an approach similar to that described elsewhere on a related molecule.^53^ We initially focused on the ^13^C carbon region, because the main metal–ligand bonding interaction involves the 2p_π_ orbitals of the cyclopentadienyl ligands. The observed hyperfine coupling (*A*) for a given ^13^C nucleus of the Cp rings is taken as the sum of contributions from spin density at that carbon *n* (*A*^Cn^) and the point dipole (through space) interaction with spin density at other atoms (*A*^dip^). The dipolar contributions, based on a point dipolar model, are given by
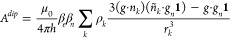


where **g**and *g*_n_**1** are the electronic and nuclear ***g*** matrices (*g*_n_ is the nuclear *g*-value; **1** is the unit matrix), β_e_ and β_n_ are the electron and nuclear magnetons, ρ_k_ is the spin population at the atom *k*, ***n*_k_** a is the n···k unit vector (expressed in the molecular frame), *r_k_is n...k* distance, *h* is Planck’s constant and μ_0_ the vacuum permittivity.

**Figure 7:**
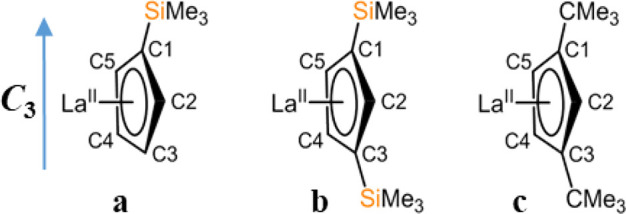
Schematic representation of the (a) La-Cp′ (in **1**), (b) La-Cp″ (in **2**) and (c) La-Cp^tt^ (in **3**) along with the labels used for the HYSCORE simulations and the orientation of the *C*_3_ axis.

Assuming dominant spin density on the La(II) ions (ρ_La_ = 1), the *A*^dip^ interactions of each carbon position in the cyclopentadienyl ring were calculated based on the crystallographic coordinates. The molecular axis system was defined in reference to the *g*-tensor, with the *g*_*z*_ component lying along the *pseudo*-*C*_3_ axis (Figure 7).

Calculated spectra based purely on these calculated *A*^dip^ matrices do not match the experimental data (Figures S64–S73). Hence, an additional contribution to the ^13^C hyperfines was introduced via *A*^*Cn*^. We assumed this additional contribution to be axial with the unique axis aligned with the 2p_π_ direction (i.e., in the molecular *xy* plane), giving *A*_∥_ and *A*_⊥_ as parameters for each C atom.

For complex **2**, good simulation of the experimental ^13^C HYSCORE spectra was achieved with A_∥, ⊥_^C2^ = 9.0, 1.2 MHz and A_∥, ⊥_^C1,3^ = 0.48, −1.44 MHz (Figure 6c and S67), with negligible densities considered for C^4^ and C^5^. For complex **3**, we found good agreement with A_∥, ⊥_^C2^ = 5.9, 0.5 MHz, A_∥, ⊥_^C1,3^ = −0.4, −1.5 MHz and A_∥, ⊥_^C4,5^ = 0.56, 0.28 MHz (Figure 6d and Figure S71). For **2** and **3**, which have the same 1,3-substitution pattern on the Cp rings, with the C2 position locked in the xy plane, previous work on related compounds has shown the C2 > C1,3 > C4,5 pattern as taken here,^53^ and the dominant C2 position can be rationalized by molecular orbital theory treatments of [M(Cp)_3_].^54^ For **1**, we found good simulations with *A*_∥, ⊥_^C2,5^ = 7.0, 2.3 MHz and *A*_∥, ⊥_^C3,4^ = 4.2, 0.65 MHz (Figure 6a; at g_*xy*_). For this complex, we also observe hyperfine to the ^29^Si atoms of the SiMe_3_ groups, quantified as *A*_iso_(Si)= −4.6 MHz (Figure 6a and Figure S64). This indicates that ^29^Si can contribute to spin decoherence in this compound. For the z-orientation, we measure weaker ^13^C hyperfines (*A*_∥, ⊥_^C1^ = 1.38,1.02 MHz *A*_∥, ⊥_^C2,5^ = 1.42, 0.27 MHz and *A*_∥, ⊥_^C3,4^ = 2.61, 0.36 MHz), and no ^29^Si coupling is observed (Figure 6b and Figure S66). From these values, the 2p_π_ spin population (ρ_p_) for each carbon atom on the ring can be estimated from , where *P*_p_ is the electron nuclear dipolar coupling parameter for unit population (ρ_p_ = 1) of a ^13^C 2p orbital. With *P*_p_ = 268 MHz,^39^ and the hyperfine values deducted by ^13^C HYSCORE, we obtained the population at C atoms on cyclopentadienyl rings in **1**–**3** (Table 2).

**Table 2:** Spin Hamiltonian parameters via HYSCORE experiments for 1, 2, and 3 (10 mM MeTHF).^a^

	**1**-La	**2**-La	**3**-La
**C**^**1**^	-	*A*_∥_ = 0.48	*A*_∥_ = −0.4
		*A*_⊥_ = −1.44	*A*_⊥_ = −1.5
		ρ_P_ = 0.007	ρ_P_ = 0.003
**C**^**2**^	*A*_∥_ = 7.0	A_∥_ = 9.0	*A*_∥_ = 5.9
	*A*_⊥_ = 2.3	A_⊥_ = 1.2	*A*_⊥_ = 0.5
	ρ_P_ = 0.015	ρ_P_ = 0.024	ρ_P_ = 0.017
	H^2^ α_iso_ = −1.227	H^2^ α_iso_ = −2.037	H^2^ α_iso_ = −1.41
**C**^**3**^	*A*_∥_ = 4.2	*A*_∥_ = 0.48	*A*_∥_ = −0.4
	*A*_⊥_ = 0.65	*A*_⊥_ = −1.44	*A*_⊥_ = −1.5
	ρ_P_ = 0.011,	ρ_P_ = 0.007	ρ_P_ = 0.003
	H^3^ α_iso_ = −0.927		
**C**^**4**^	*A*_∥_ = 4.2	-	A_∥_ = 0.56
	*A*_⊥_ = 0.65		A_⊥_ = 0.28
	ρ_P_ = 0.011		ρ_P_ = 0.0009
	H^4^ α_iso_ = −0.927		H^4^ α_iso_ = −0.073
**C**^**5**^	A_∥_ = 7.0	-	*A*_∥_ = 0.56
	A_⊥_ = 2.3		*A*_⊥_ = 0.28
	ρ_P_ = 0.015		ρ_P_ = 0.0009
	H^5^ α_iso_ = −1.227		H^5^ α_iso_ = −0.073

a All hyperfine constants are provided in MHz.

DFT calculations using the crystal structures of **1**-**3** provide estimates of ^13^C hyperfine coupling and C spin density on the Cp^R^ rings (Tables S15–S20), which are on the order of the values determined from the model of the HYSCORE data here. Notably, a large *A*_∥_ is obtained for **C^5^** in **1** and for **C^2^** in **2** and **3**, in agreement with the HYSCORE model. However, these calculations account for the asymmetry of the solid-state structures, which have significant influences on the calculated spin densities and ligand hyperfine couplings and we also observe some rhombic ^13^C hyperfine couplings. Hence, we have optimized the geometries of **1**-**3** in the gas-phase and recalculated the hyperfine coupling and atomic spin densities (Tables S21–S28). There are very little changes when it comes to the ^139^La hyperfine, and some ^13^C couplings on the Cp^R^ rings are still very rhombic. But overall, for complexes **2** and **3** which have the most sterically demanding ligands, we observe that the ligand hyperfine is not much different than that obtained with the crystal structures, and that the three ligands now show more symmetric values. For **1** however, there is a larger deviation between the two calculations, presumably owing to greater molecular flexibility, and we also find that the calculated values for one ligand in the optimized geometry are quite different than for the other two ligands, likely a result of the particular conformational minimum obtained in the optimization. Given the significant dependence of the calculated hyperfine coupling and spin densities on the structural details and that the experiments are performed on amorphous frozen solution samples with inherent molecular distributions, it is not assured that a simple model such as the one employed here should work. That it manages to capture similar results compared to the DFT calculations is quite remarkable.

For the ^1^H HYSCORE spectra, the point dipole-only model again failed to reproduce the experimental data, although dipolar interactions are larger because of the larger magnetic moment of ^1^H *cf*. ^13^C. Thus, in order to reproduce the spectra we added a hyperfine contribution due to the spin density on the carbon to which it is bound via spin polarization.^55^ The hyperfine matrix to an α proton in organic π-radicals typically takes the form [α_iso_/2, α_iso_, 3α_iso_/2], where the smallest component is oriented along the C–H bond, the middle one along the 2p_π_ direction, and the largest along the cross-product of the 2p_π_ and C–H orientation. With this model, with a single variable (α_iso_) for each ^1^H, excellent simulations of the ^1^H spectra were obtained (Figure 6e–h). For **1**, we found α_iso_ = −1.227 MHz for H^2,5^ and α_iso_ = −0.927 MHz for H^3,4^ (Figure 6d). For complexes **2** (Figure 6g,h) and **3** (Figure S72 data were satisfactory simulated with α_iso_ = −2.037 and −1.41 MHz for H^2^, respectively (contributions from the protons attached to C^4,5^ are negligible due to the negligible densities at these carbons, and C^1,3^ have no attached protons). The isotropic hyperfine constant α_iso_ at the α-proton is linked to the spin density at the associated C 2p_π_ orbital by the simple McConnell relationship, α_iso_ = Q_CH_·ρ_P_,^56^ where *Q*_CH_ is the ^1^H hyperfine coupling that would be observed for ρ_P_ = 1. With *Q*_CH_ = −84 MHz from studies of Cp radicals,^57^ we obtained spin densities at C which agree with those derived from the ^13^C data (Table 2), providing a consistent analysis.

Summing up the HYSCORE results, there are considerable differences in the electron spin density measured at ligand atoms. The total measured carbon-2p_π_ spin population on the three ligands is estimated to be 15.6%, 11.4% and 7.4% for **1**, **2** and **3**, respectively, which is in agreement with the trend predicted by DFT calculations, of *ca*. 13.5% (**1**) > 12.0% (**2**) ≫ 5.97% (**3**) (Tables S15–S23). These results are at first unexpected as **1** and **2** contain one and two SiMe_3_ groups, respectively, while **3** contains two CMe_3_ groups. As the silyl substituents are electron withdrawing in cyclopentadienyl rings the order **2** > **1** ≫ **3** might have been predicted. On the other hand, comparing **2** and **3** which have the same substitution pattern, we find that there is substantially greater ligand spin population in **2** than in **3**. Recalling that the metal hyperfine for **2** is much smaller than that for **3**, the conclusion is that the electron-withdrawing nature of the SiMe_3_ groups in **2** (cf. to electron-donating CMe_3_ groups in **3**) leads to a greater ligand spin density and a smaller metal spin density. This is not what one would predict based on a simplistic MO argument for [M(Cp)_3_] complexes,^58^ where the decrease in energy of the Cp^R^π-orbitals would lead to a poorer energy match with the metal 5d_*z*_^2^ that dominated the SOMO and hence less ligand character in the a_1_ SOMO. It is more difficult to make arguments for complex **1** because the different substitution pattern affects the pattern of spin distribution. It is interesting that despite the greater ligand character in **2** (and **1**) than in **3**, the electron spin relaxation times for **3** are longer. This would be consistent with the metal s-character being more important.

## Conclusions

Pulsed EPR studies on three organometallic La^II^ complexes based on Cp′ (**1**), Cp″ (**2**), and Cp^tt^ (**3**) are reported. By modifying the chemical structure of the molecules, we investigated the effect of various substituents on the spin dynamics. We found the spin–lattice relaxation time *T*_1_ and the electronic coherence *T*_CPMG_ times to vary in line with the 6s-orbital character of SOMO, that is 6.5% (**2**) < 7.1% (**1**) ≪ 10.6% (**3**). At 5 K, *T*_1_ is incredibly long, varying from *ca*. 7 ms (**2**) to 19 ms (**1**) and, more importantly, to 50 ms for **3**. *T*_CPMG_ reaches 36 and 49 μs at 5 K for **1** and **2** respectively, increasing to 161 μs for **3**. Notably, the same trend is observed at 80 K, with the highest *T*_CPMG_ value (4 μs) being measured for **3**, which has the smallest spin delocalization onto ligands and the largest 6s character of La(II) single occupied molecular orbital (SOMO) most likely due to the strong electron-donating nature of CMe_3_ substitutes. *T*_1_ is also largest for **3**, whose SOMO has a significant s-orbital character (10.6%), which reduces the effect of SOC via metal. Complex **1** also has a longer *T*_1_ than complex **2**, and has greater La 6s-orbital and ligand character than **2.** Phase memory time values display only small differences between the three complexes, and the largest value (2.4 μs) is measured for **3**. We find *T*_m_ to be limited by interactions with the environmental nuclear spins, and used advanced HYSCORE techniques to quantify such interactions. We measure significant spin density at the ^1^H protons of Cp rings, and ^29^Si of SiMe_3_ groups (1), indicating that these nuclei participate to decoherence. Coherent spin manipulations were probed for up to eight hyperfine transitions for **1**–**3**, with Rabi oscillations observed up to 80 K. Coherence times can be extended by CMPG methods. Additionally, similar NOT operation times and Q_M_ values were determined for all complexes, with **1** implementing a faster inversion of the qubit and thus, allowing a higher number of single–qubit NOT computational operations in a given time.

For future studies on ^139^La, there are significant advantages to growing magnetically diluted crystals of **1**–**3** as measurement of relaxation times and manipulation of the electron and nuclear spins should be achievable up to room temperature for these prototype qu*d*its. Using Davis ENDOR one can explore the coherent spin properties of ^139^La nuclear spins, including nuclear spin Rabi oscillations, which will provide significant insight into the dynamics of the nuclear spins, allowing us to also measure the nuclear spin relaxation and the nuclear spin coherence times. These experiments are feasible and were used to study the coherent dynamics of ^171^Yb ions in yttrium orthosilicate.^64^

## Experimental Section

### Experimental materials and methods

All manipulations and syntheses were conducted with rigorous exclusion of air and water using standard Schlenk line and glovebox techniques under an argon or dinitrogen atmosphere. The preparation and characterization of complexes **1**–**3**^3,32,33^ and KC_8_^59^ followed previously reported methods. All glassware was flame-dried under vacuum or stored overnight in a hot oven prior to use. Argon and dinitrogen were passed from cylinders through columns of activated 3 Å molecular sieves and Cu catalyst prior to use. MeTHF was refluxed over molten K for 3 days, distilled, and stored over activated 4 Å molecular sieves. As expected for early metal organometallic complexes, **1**–**3** are highly air- and moisture-sensitive.^2^ In addition, all three complexes are temperature-sensitive and ethereal solutions have been reported to decompose rapidly above −20 °C,^3,32,33^ in common with other similar trigonal planar La(II) complexes.^5,6^ All samples for frozen solution EPR spectroscopy were prepared under strict anaerobic conditions and measured in flame-sealed quartz EPR tubes to avoid oxidation of La^2+^ to La^3+^, and to enable safe investigation. Two different sample preparation procedures were followed. For samples of **1** and **2**, aliquots of 10 mM MeTHF solutions of the respective complex at −30 °C were transferred to precooled quartz EPR tubes (to a sample height of *ca*. 5 cm) within an Ar glovebox. These tubes were quickly attached to an appropriate set of apparatus to make an airtight seal, removed from the glovebox, and the solutions flash-frozen in a Dewar containing liquid nitrogen. The frozen solutions were placed under vacuum on a Schlenk line to the point at which the apparatus could maintain a constant static pressure of less than 5 × 10^–3^ mbar, and then flame-sealed under a dynamic vacuum. For the sample of **3**, a solution of [La(Cp^tt^)_3_] in MeTHF in a sample vial in a glovebox at −30 °C was treated with KC_8_ and stirred for 10 min at this temperature to give a *ca*. 10 mM solution of **3**. An aliquot of the suspension was transferred to a precooled quartz EPR tube (to a sample height of *ca*. 5 cm), and flame-sealed under dynamic vacuum following the same method as above. Frozen solution samples were stored in liquid nitrogen until measurement.

The c.w. EPR spectra were recorded at X-band (ca. 9.4 GHz) mw frequency using a Bruker EMX 300 EPR spectrometer equipped with a 1.8 T electromagnet and a Stinger closed-cycle helium gas cryostat. Field corrections were applied using Bruker strong pitch (*g* = 2.0028) as a reference, and spectra were baselined before simulation. EPR spectra were simulated using the EasySpin toolbox implemented within Matlab.^34^ The simulation model has assumed *S* = 1/2 for **1**–**3** (5d^1^), with axial (*g*_*x*_ = *g*_*y*_ ≠ *g*_*z*_) g-tensors. Where hyperfine coupling was resolved, an anisotropic hyperfine coupling, *A* = [*A*_*x*_, *A*_*x*_, *A*_*3*_], was included in the model, with *A* and *g* tensors assumed to be collinear. Line broadening is the result of a distribution of *g*-values, unresolved hyperfine coupling/a distribution of *A-*values, and dipolar coupling, among other effects. Anisotropic line broadenings have been modeled with phenomenological *g*-strains (distribution of *g*-values) to account for all broadening effects. Pulsed EPR X-band studies were performed on a Bruker ElexSys E580 spectrometer. The primary Hahn echo sequence *(π/*2-τ–π–τ-*echo*)^40^ was used for the two-pulse electron spin echo measurements, with initial π/2 and π pulse of 16 and 32 ns, respectively. For the relaxation time measurements, *T*_m_ studies were made by incrementing the τ time in the Hahn echo sequence (longer pulses were used to suppress the ^1^H modulation). *T*_1_ was measured by the inversion recovery sequence *(π-t-π/*2*-τ–π–τ-echo*)^40^ with π/2 and π pulses of 16 and 32 ns, respectively, with τ = 300 ns and varying *t*. The dynamic decoupling measurements were carried out by a CPMG sequence *(π/*2*-τ-(π-*2*τ)*_n_*-π–τ-echo*) with n = 300, π/2 and π pulse of 16 and 32 ns, respectively, and using 16-step phase cycling. HYSCORE measurements were performed using the four-pulse sequence (π/2*-τ-*π/2*-t*_*1*_*-π-t*_*2*_*-π/*2*-echo*),^40^ π/2 and π pulse of 16 and 32 ns, respectively, initial times *t*_1,2_ = 0.1 μs and τ values of 136 and 200 ns.

### Computational methods

Density functional theory (DFT) calculations were performed with the TPSSH functional and the ZORA relativistic Hamiltonian^60^ with Orca 5.0.4.^61^ The ZORA-def2-TZVP basis set^62^ was used for all nonmetal atoms, and the SARC-ZORA-TZVP basis set^63^ was used for La. Calculations were performed either on nonoptimized crystal structures, or optimized geometries using the above method in conjunction with the COSMO solvent model for THF. Hyperfine couplings were calculated using picture change corrections. We find Löwdin population analysis for the La components is the closest to the traditional EPR analysis methods and is consistent with our usual MO analysis techniques, however we find that the Mulliken spin density values of the ligand atoms correlate with their DFT-calculated hyperfine couplings (where the equivalent Löwdin values do not) and so we use the latter there.

## Supporting Information Available

Molecular structures of complexes **1**, **2** and **3**, continuous-wave electron paramagnetic resonance spectra, additional echo-detected field-swept spectra, spin–lattice relaxation measurements, phase memory time measurements, additional transient nutation data, HYSCORE spectra and simulations, and DFT data are available in the Supporting Information (PDF)
