# Supplementary material for: Ligand Effects on the Spin Relaxation Dynamics and Coherent Manipulation of Organometallic La(II) Potential Qudits
Source: J Am Chem Soc. 2024 May 24;146(22):15000–9. doi: 10.1021/jacs.3c12827 (PMC11157535; doi:10.1021/jacs.3c12827)
Supplement: Supplementary file 1 — ja3c12827_si_001.pdf [file ja3c12827_si_001.pdf]

Electronic Supplementary Information for:

Ligand Effects on the Spin Relaxation Dynamics and Coherent Manipulation of Organometallic La(II) Potential Qudits

Lydia E. Nodaraki,[1,2] Ana-Maria Ariciu,[1,2] Daniel N. Huh,[3,4] Jingjing Liu,[1] Daniel O. T. A. Martins,[1,2] Fabrizio Ortu,[1,5] Richard E.P. Winpenny,[1] Nicholas F. Chilton,[1,6] Eric J. L. McInnes,[1,2] David P. Mills,[1,*] William J. Evans[3,*] and Floriana Tuna[1,2,*]

[1] Department of Chemistry, University of Manchester, Manchester M13 9PL (UK)

[2] Photon Science Institute, University of Manchester, Manchester M13 9PL (UK)

[3] Department of Chemistry, University of California, Irvine, California 92697 (USA)

[4] Department of Chemistry, University of Rhode Island, Kingston RI 02881 (USA)

[5] School of Chemistry, University of Leicester, Leicester LE1 7RH (UK)

[6] Research School of Chemistry, Australian National University, Canberra 2617 (Australia)

Table of Contents

1. Molecular Structures of Complexes **1**, **2** and **3** S2
2. Additional EPR Spectra for Complexes **1**, **2** and **3** S5
    2.1 CW and EDFS EPR Spectra S5
    2.2 Echo-Setected Field Swept (EDFS) Spectra S7
    2.3 Spin-Lattice Relaxation Measurements S10
    2.4 Phase Memory Time Measurements S18
    2.5 Carr-Purcell-Meiboom-Gill (CPMG) Experiments S27
    2.6 Transient Nutation Experiments S32
    2.7 HYSCORE Spectra S49
3. DFT Calculations for **1**, **2** and **3** S56
4. References S61

1. Molecular Structures of Complexes 1, 2 and 3.

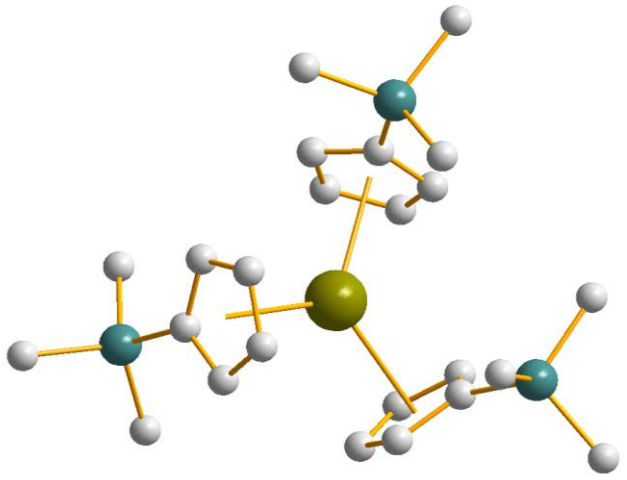

Figure S1. Molecular structure of $[La(Cp')_3]^-$ (**1**).[1] Colour code: La-gold, C-grey, Si-dark cyan. Counterions and hydrogen atoms are omitted for clarity.

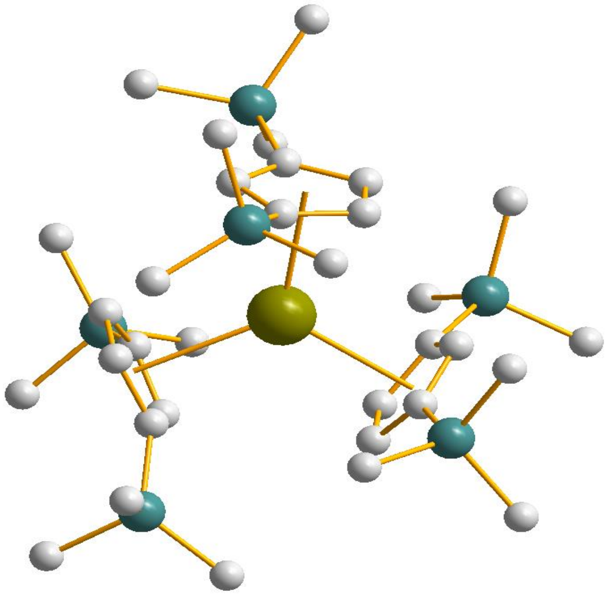

Figure S2. Molecular structure of [La(Cp")$_3$]$^-$ (**2**).[2] Colour code: La-gold, C-grey, Si-dark cyan. Counterions and hydrogen atoms are omitted for clarity.

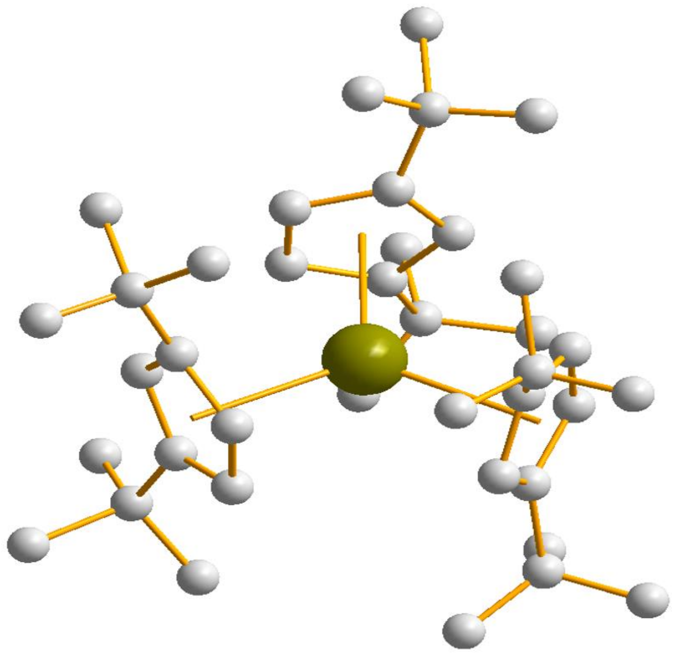

Figure S3. Molecular structure of $[La(Cp^{tt})_3]^-$ (**3**).[3] Colour code: La-gold, C-grey. Counterions and hydrogen atoms are omitted for clarity.

2. Additional EPR spectra for complexes 1, 2 and 3

2.1 Continous-wave (CW) EPR spectra

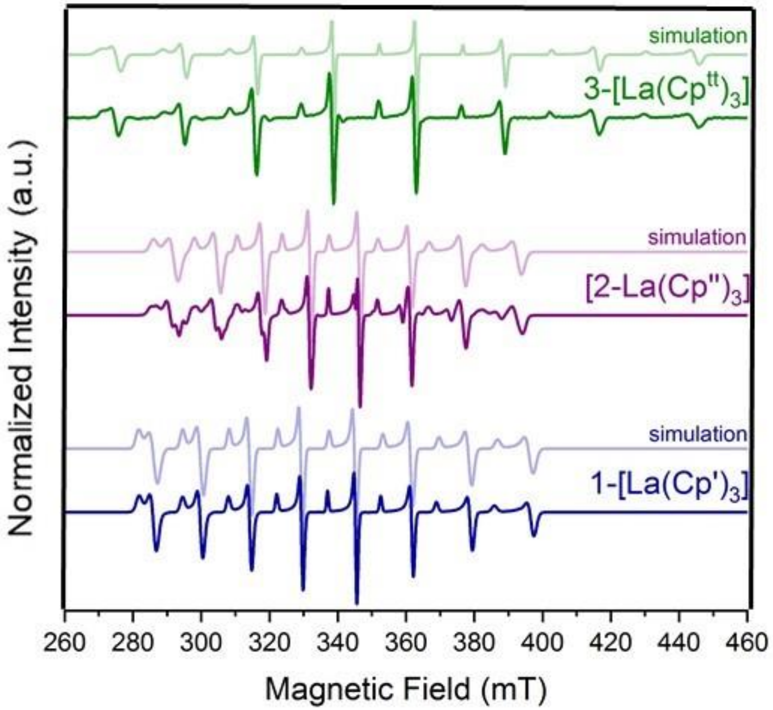

Figure S4. Experimental (bold line) and simulated (transparent line) CW EPR spectra at X-band (9.4 GHz) of frozen solutions (10 mM, MeTHF) of **1** at 80 K (blue), **2** at 80 K (purple), and **3** at 40 K (green). Simulation parameters are provided in Table 1.

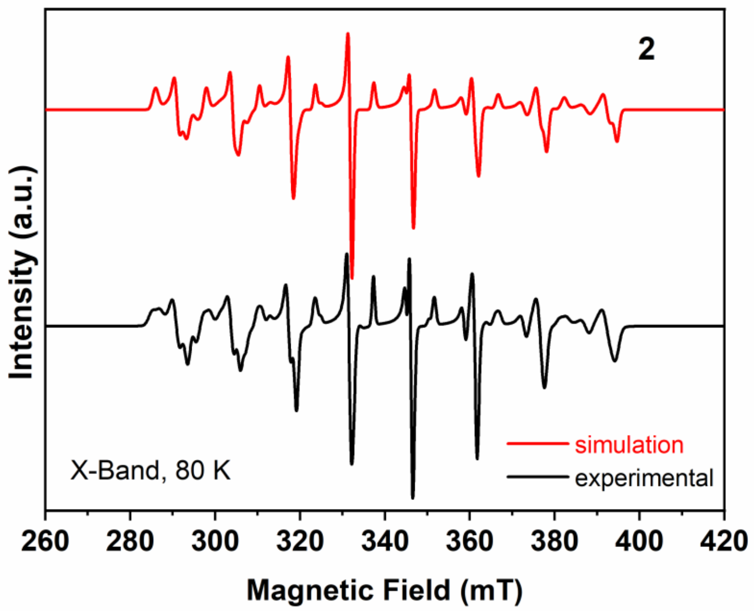

Figure S5. Experimental (black) and simulated (red) X-band (9.4 GHz) EPR spectrum for **2** in MeTHF (10 mM) at 80 K. Simulation included a small amount of a secondary La(II) species ($g_{\parallel}$ = 2.001, $g_{\perp}$ = 1.958, $A_{\parallel}$ = 352 MHz and $A_{\perp}$ = 362 MHz).

2.2 Echo-Detected Field-Swept (EDFS) spectra

EDFS spectra were recorded with a standard Hahn-echo sequence, $\pi/2$ - τ - π - τ - *echo*,[4] with $\pi/2$ and π pulse lengths of 16 ns and 32 ns, respectively, and a fixed delay time of τ = 180 ns.

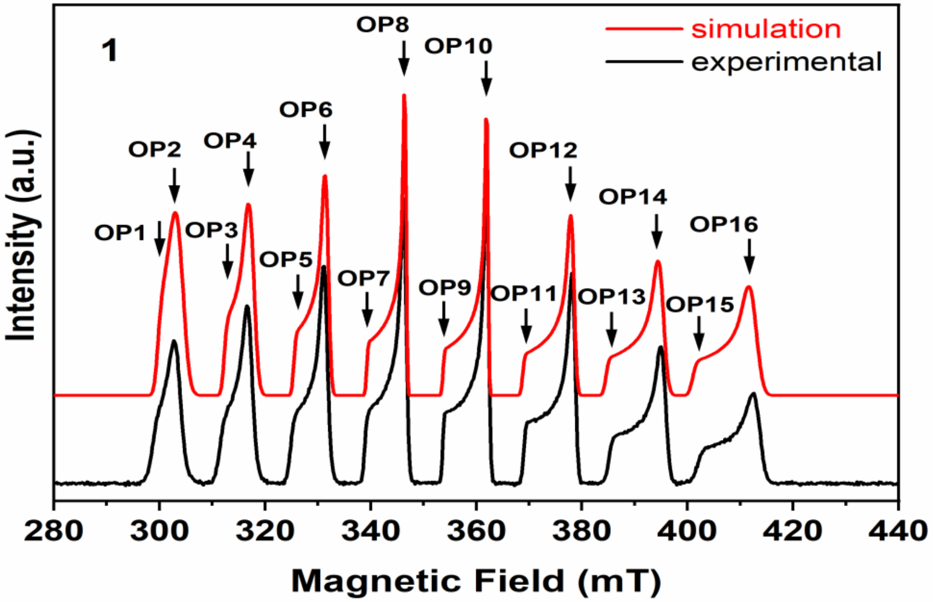

Figure S6. X-band EDFS spectrum for a frozen solution of **1** in MeTHF (10 mM) at 40 K. The experimental spectrum is shown in black, and its simulation in red. Simulation parameters are similar to CW spectra (Figure S4; Table 1). Observer positions OP1-OP16 mark the magnetic fields at which further pulse EPR experiments were performed.

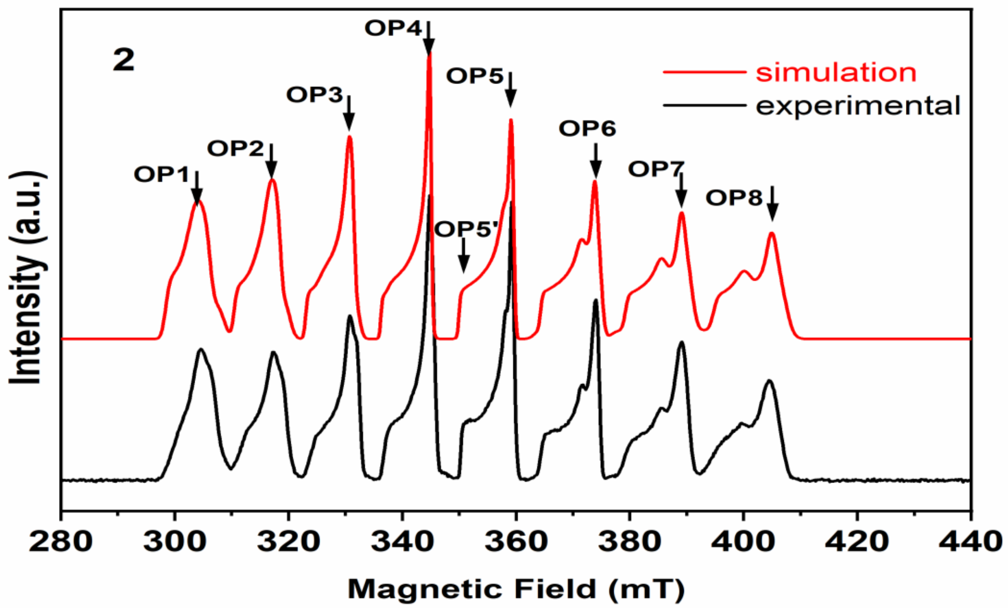

Figure S7. X-band EDFS spectra for a frozen solution of **2** in MeTHF (10 mM) at 40 K. The experimental spectrum is shown in black, and its simulation in red. Simulation parameters are identical to those for CW spectra (Figure S5; Table 1). Observer positions OP1-OP8 and OP5' mark the magnetic fields at which further pulse EPR experiments were performed.

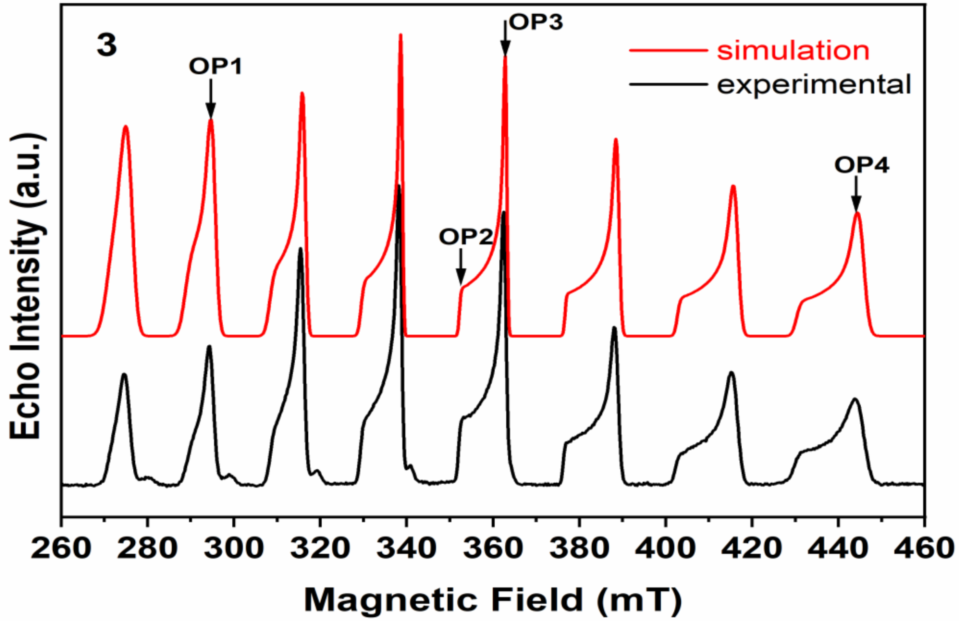

Figure S8. X-band EDFS spectra for a frozen solution of **3** in MeTHF (10 mM) at 40 K. The experimental spectrum is shown in black, and its simulation in red. Simulation parameters are identical to those for CW spectra (Figure S4; Table 1). Observer positions OP1-OP4 mark the magnetic fields at which further pulse EPR experiments were performed.

2.3 Spin-Lattice Relaxation Measurements

Measurements of the spin–lattice relaxation time (T_1) were carried out with a magnetization inversion recovery pulse sequence (π–t–π/2–τ–π–τ–*echo*)[4] with π/2 and π pulse lengths of 16 and 32 ns, respectively, fixed τ and variable t. The time constant T_1 was extracted by fitting the experimental data according to the equation (1):[4]

$$Y(t) = Y(0) + Y_1 e^{(-t/T_1)} + Y_{SD} e^{(-t/T_{SD})} \quad (1)$$

where Y_1 and Y_{SD} are the amplitudes and T_{SD} is the spectral diffusion time constant. Results are presented in Figures S9-S17 and Tables S1-S3.

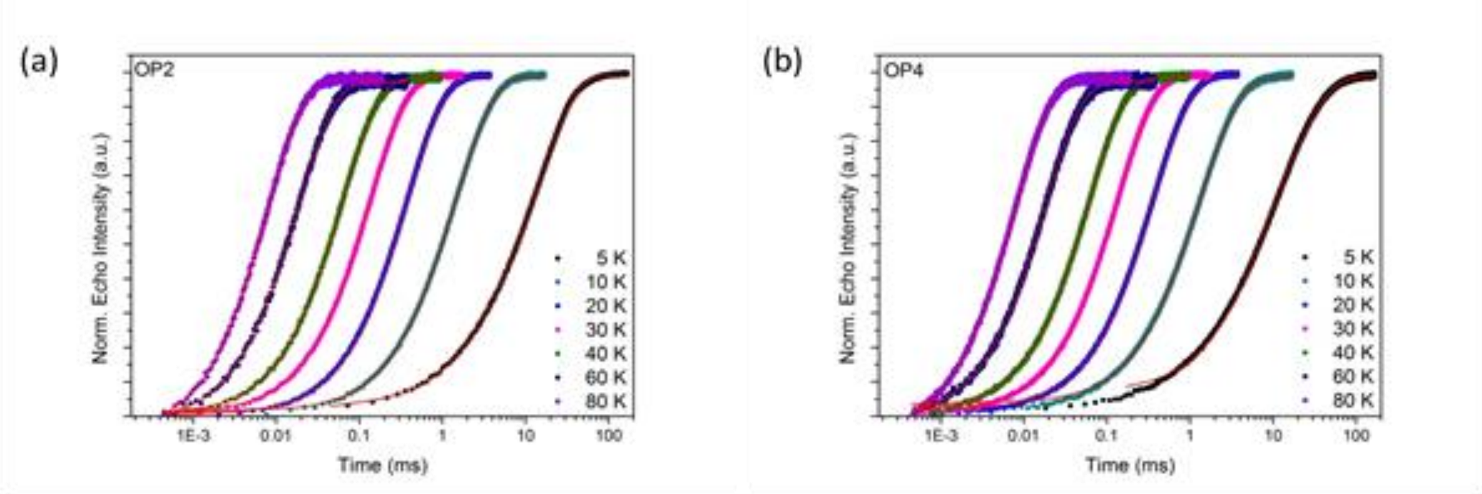

Figure S9. X-band inversion recovery curves for **1** at (a) OP2, and (b) OP4 (Figure S6), at the indicated temperatures. The red lines represent the best fitting of the data to the biexponential model equation (1), with the parameters in Table S1.

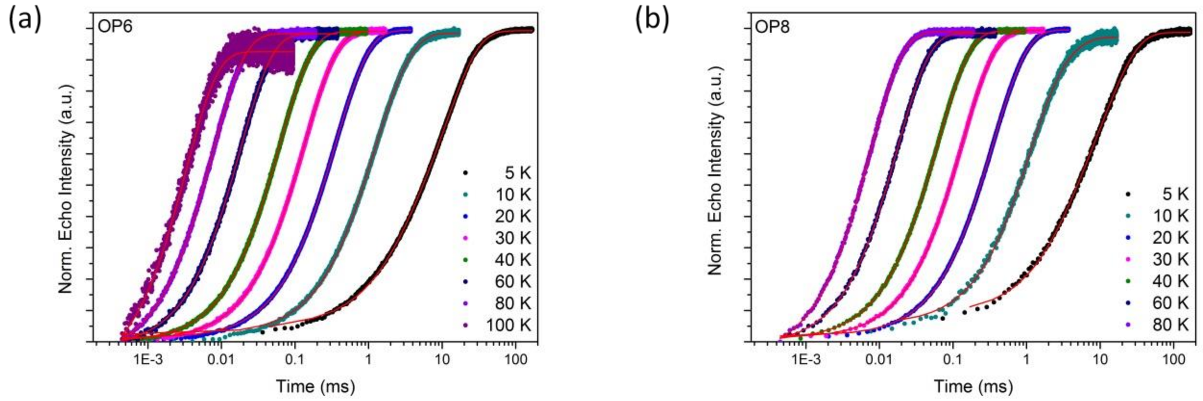

Figure S10. X-band inversion recovery curves for **1** at (a) OP6, and (b) OP8 (Figure S6), at the indicated temperatures. The red lines represent the best fitting of the data to the biexponential model equation (1), with the parameters in Table S1.

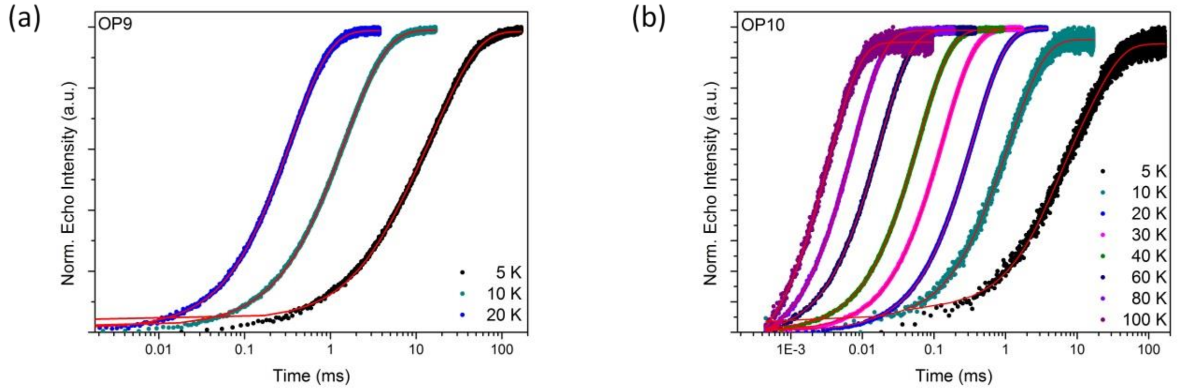

Figure S11. X-band inversion recovery curves for **1** at (a) OP9, and (b) OP10 (Figure S6), at the indicated temperatures. The red lines represent the best fitting of the data to the biexponential model equation (1), with the parameters in Table S1.

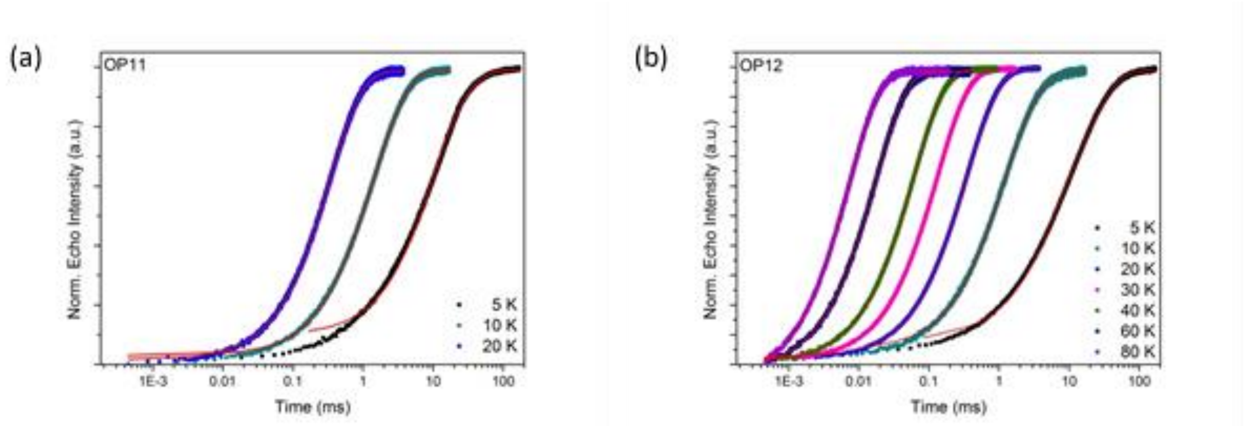

Figure S12. X-band inversion recovery curves for **1** at (a) OP11, and (b) OP12 (Figure S6), at the indicated temperatures. The red lines represent the best fitting of the data to the biexponential model equation (1), with the parameters in Table S1.

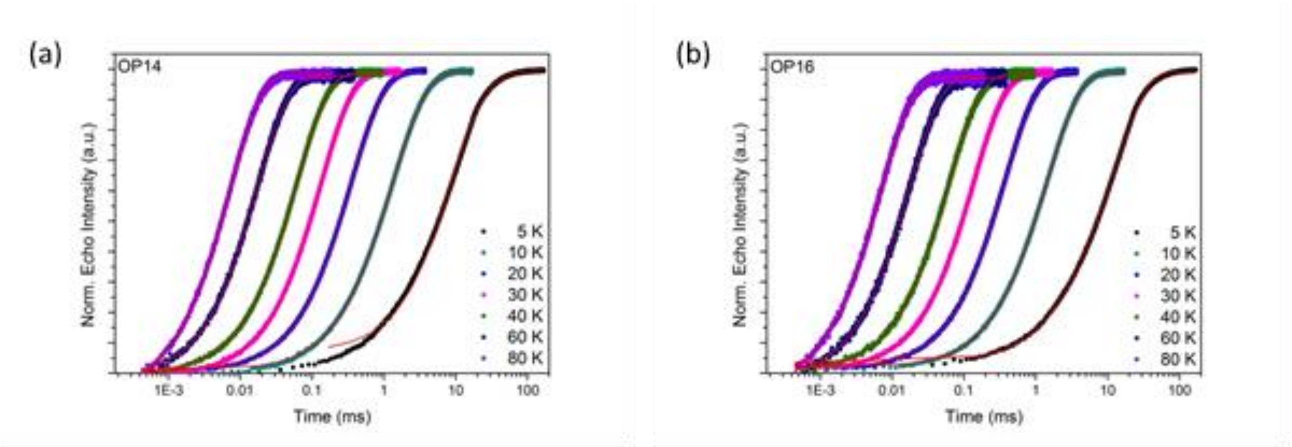

Figure S13. X-band inversion recovery curves for **1** at (a) OP14, and (b) OP16 (Figure S6), at the indicated temperatures. The red lines represent the best fitting of the data to the biexponential model equation (1), with the parameters in Table S1.

Table S1. Extracted spin-lattice relaxation time constants (T_1) for **1** in μs (10 mM, MeTHF) at X-band, based on Figures S9 –S13.

T(K)	OP1		OP2		OP3		OP4	
	T_1 (μs)	T_{SD} (μs)	T_1 (μs)	T_{SD} (μs)	T_1 (μs)	T_{SD} (μs)	T_1 (μs)	T_{SD} (μs)
5	24781	5006	14982	1963	17419	2387	19396	5237
10	1888	743	1699	627	1891	773	1855	857
20	416	234	408	161	389	147	420	219
30			146	62			149	79
40			68	38			67	43
60			20	10			20	12
80			9	6			8	6

T(K)	OP5		OP6		OP7		OP8	
	T_1 (μs)	T_{SD} (μs)	T_1 (μs)	T_{SD} (μs)	T_1 (μs)	T_{SD} (μs)	T_1 (μs)	T_{SD} (μs)
5	28448	2845	11860	1930	26998	6482	12616	2724
10	1848	712	1730	743	1900	777	2360	912
20	390	167	409	190	392	174	406	184
30			149	84	133	13	145	73
40			67	40	52	3	64	20
60			24	16	20	15	60	12
80			8	6	5	2	8	6

T(K)	OP9		OP10		OP11		OP12	
	T_1 (μs)	T_{SD} (μs)	T_1 (μs)	T_{SD} (μs)	T_1 (μs)	T_{SD} (μs)	T_1 (μs)	T_{SD} (μs)
5	21008	4251	16654	3400	22785	4514	19017	4529
10	1879	747	1429	411	1879	725	2403	965
20	393	183	399	180	394	183	401	187
30	124	41	149	87			147	79
40	53	36	79	54			65	35

60	18	4	21	15			16	11
80	5	1	10	7			8	6

T(K)	OP13		OP14		OP15		OP16	
	T_1 (μs)	T_{SD} (μs)	T_1 (μs)	T_{SD} (μs)	T_1 (μs)	T_{SD} (μs)	T_1 (μs)	T_{SD} (μs)
5	16390	2807	24863	8217	18885	2400	13143	1768
10	1916	793	1667	664	2114	1056	1656	600
20	392	168	413	209	401	185	409	187
30			145	74			148	74
40			65	33			66	31
60			19	14			18	4
80			7	4			8	5

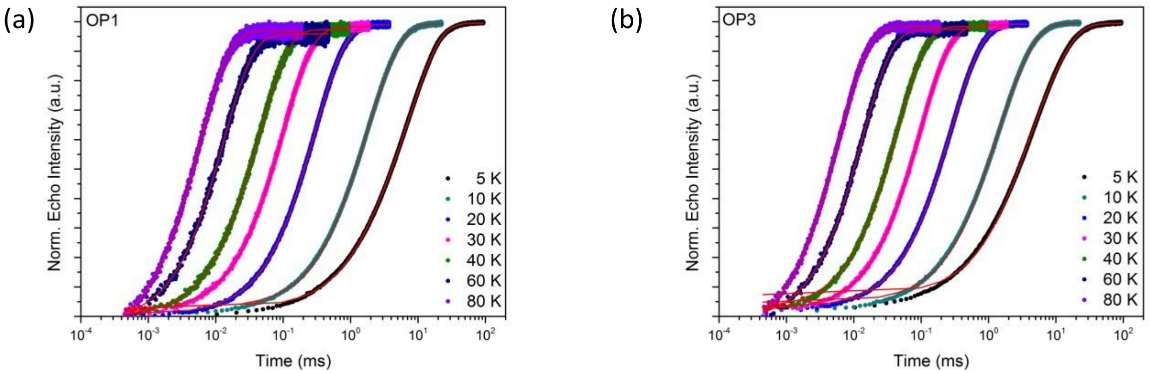

Figure S14. X-band inversion recovery curves for **2** at (a) OP1, and (b) OP3 (Figure S7), at the indicated temperatures. The red lines represent the best fitting of the data to the biexponential model equation (1), with the parameters in Table S2.

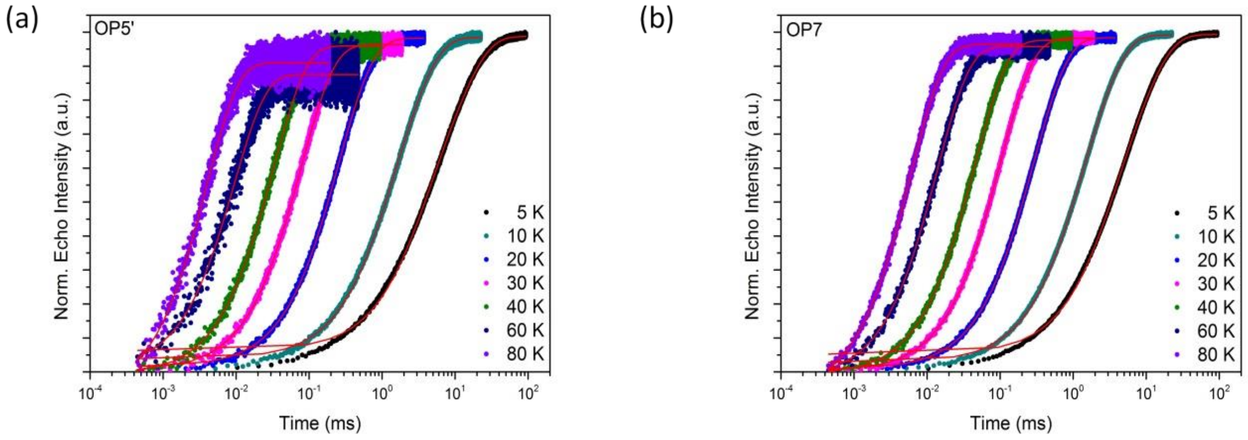

Figure S15. X-band inversion recovery curves for **2** at (a) OP5’, and (b) OP7 (Figure S7), at the indicated temperatures. The red lines represent the best fitting of the data to the biexponential model equation (1), with the parameters in Table S2.

Table S2. Extracted spin-lattice relaxation time constants (T_1) for **2** in μs (10 mM MeTHF) at X-band (9.7 GHz), based on Figures S14–S15.

T(K)	OP1		OP2		OP5’		OP5		OP7	
	T_1 (μs)	T_{SD} (μs)	T_1 (μs)	T_{SD} (μs)	T_1 (μs)	T_{SD} (μs)	T_1 (μs)	T_{SD}(μs)	T_1 (μs)	T_{SD}(μs)
5	8062	1407	8005	2127	11211	2764	7295	1953	8878	2474
10	2297	860	2169	758	2501	854	1968	677	2156	780
20	344	186	341	163	333	148	-	-	332	143
30	121	81	129	82	88	46	117	74	120	76
40	46	17	44		34	9	65	41	48	17
60	27	12	39	13	9		15	10	15	9
80	7	5	8	5	4		8	6	7	4

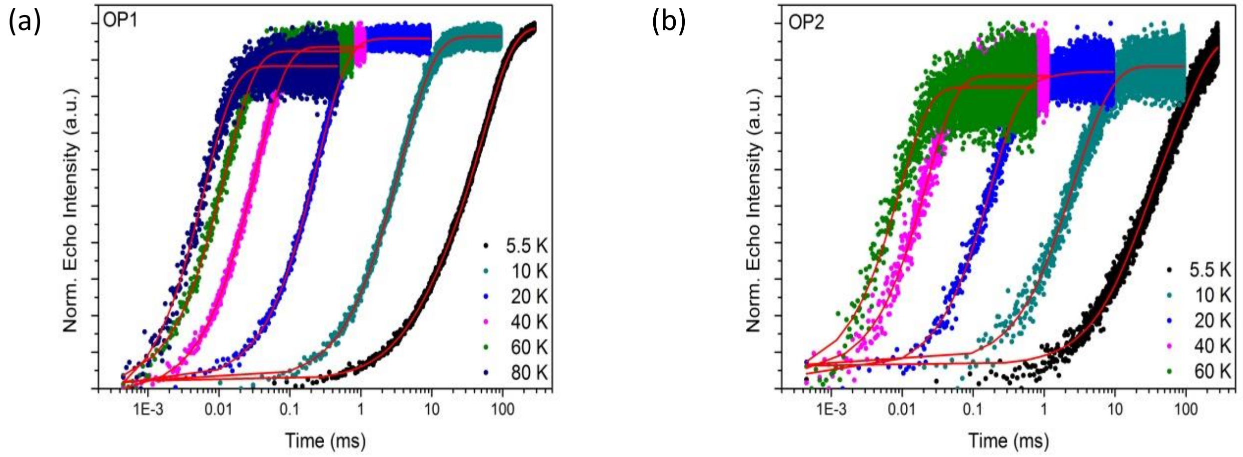

Figure S16. X-band inversion recovery curves for **3** at (a) OP1, and (b) OP2 (Figure S8), at the indicated temperatures. The red lines represent the best fitting of the data to the biexponential model equation (1), with the parameters in Table S3.

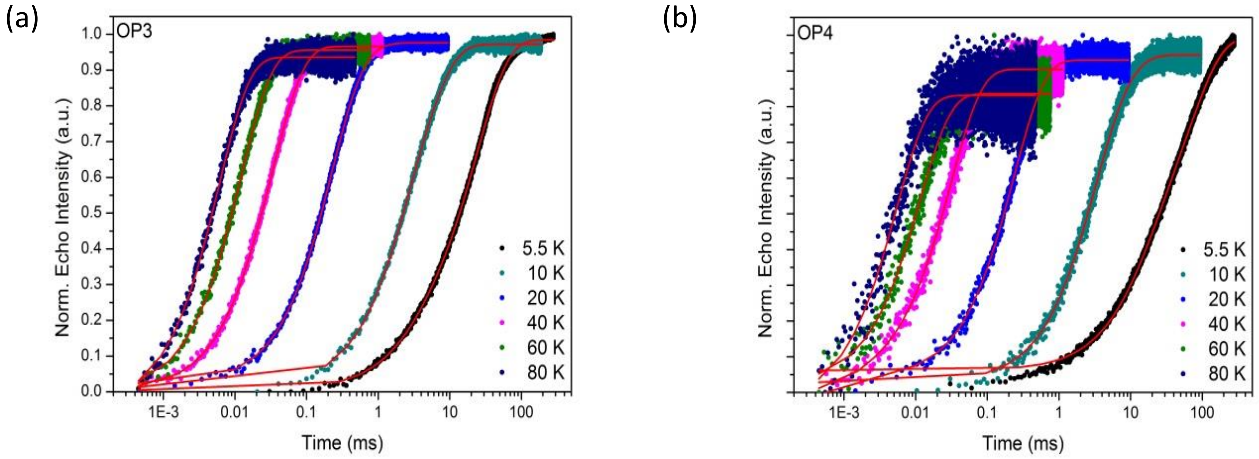

Figure S17. X-band inversion recovery curves for **3** at (a) OP3, and (b) OP4 (Figure S8), at the indicated temperatures. The red lines represent the best fitting of the data to the biexponential model equation (1), with the parameters in Table S3.

Table S3. Extracted spin-lattice relaxation time constants for **3** in μs (10 mM MeTHF) at X-band (9.7 GHz), based on Figures S16–S17.

T (K)	OP1		OP2		OP3		OP4	
	T_1 (μs)	T_{SD} (μs)	T_1 (μs)	T_{SD} (μs)	T_1 (μs)	T_{SD} (μs)	T_1 (μs)	T_{SD} (μs)
5.5	52891	6244	97780	16458	24463	2683	71807	13205
10	4478	1650	4199	1416	4752	1725	5688	2603
20	357	224	1047	173	429	212	234	2
40	33	7	22		34	12	31	2
60	12		9		12		11	
80	6				6		5	

2.4 Phase Memory Time Measurements

Measurements of the phase memory time, T_m, were carried out using a Hahn echo sequence (π/2-τ-π-τ–*echo*)[4] that implies a gradual increase of the inter-pulse delay, τ. For microwave pulse lengths of π = 32 or 128 ns, strong proton-electron spin modulation was observed. In order to suppress the ^1H modulation in the echo decays, longer microwave pulses of length π = 256 or 1000 ns were used. Data were modelled using equation (2),

$$\mathrm{Y}(2\tau) = \mathrm{Y}(0)\mathrm{e}^{(-2\tau/T_\mathrm{m})x} \quad (2)$$

where x is the stretch factor. Results are presented in Figures S18 – S30 and Tables S4-S6.

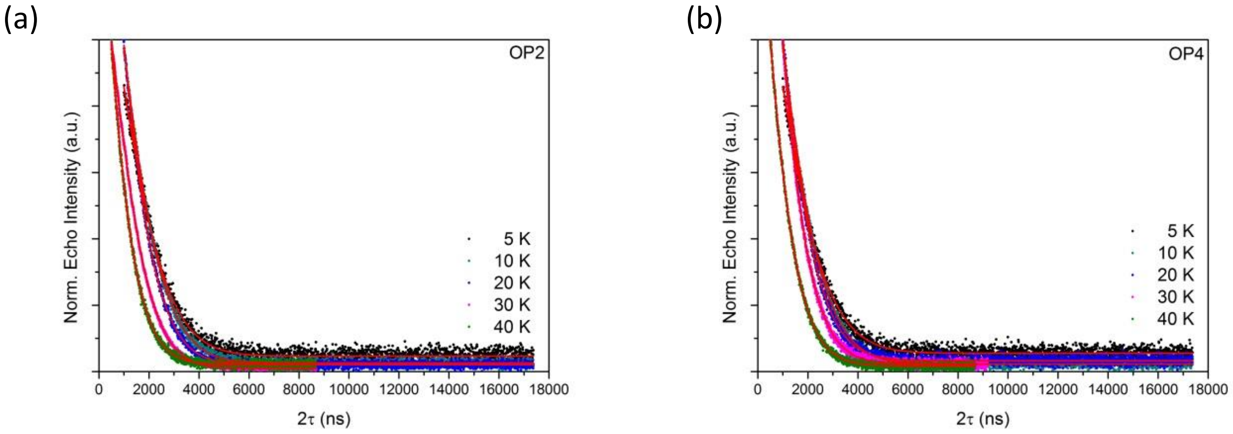

Figure S18. Normalized echo integral of **1** as a function of 2τ measured at (a) OP2 and (b) OP4 (Figure S6), at the indicated temperatures. The red lines represent the best fits to equation (2), with the parameters in Table S4.

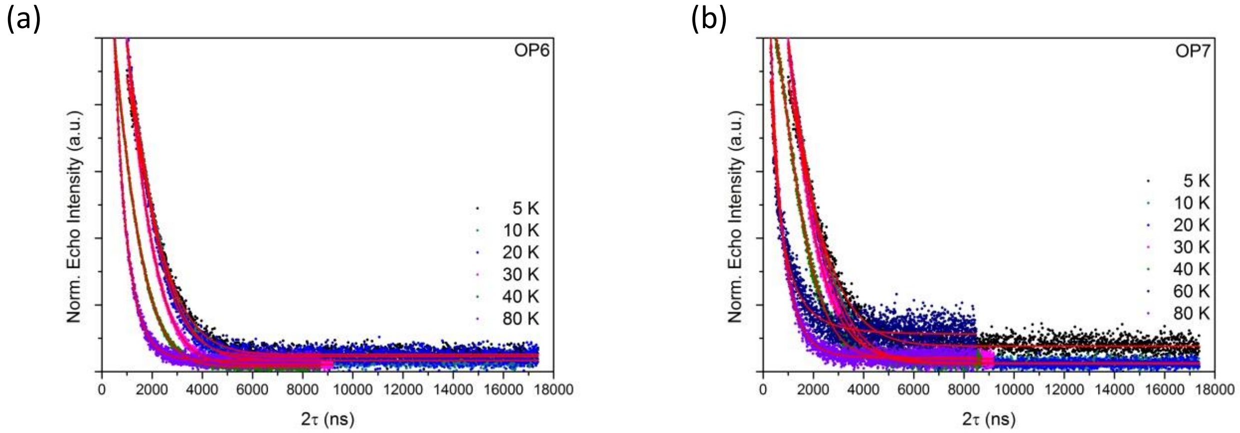

Figure S19. Normalized echo integral of **1** as a function of 2τ measured at (a) OP6 and (b) OP7 (Figure S6), at the indicated temperatures. The red lines represent the best fits to equation (2), with the parameters in Table S4.

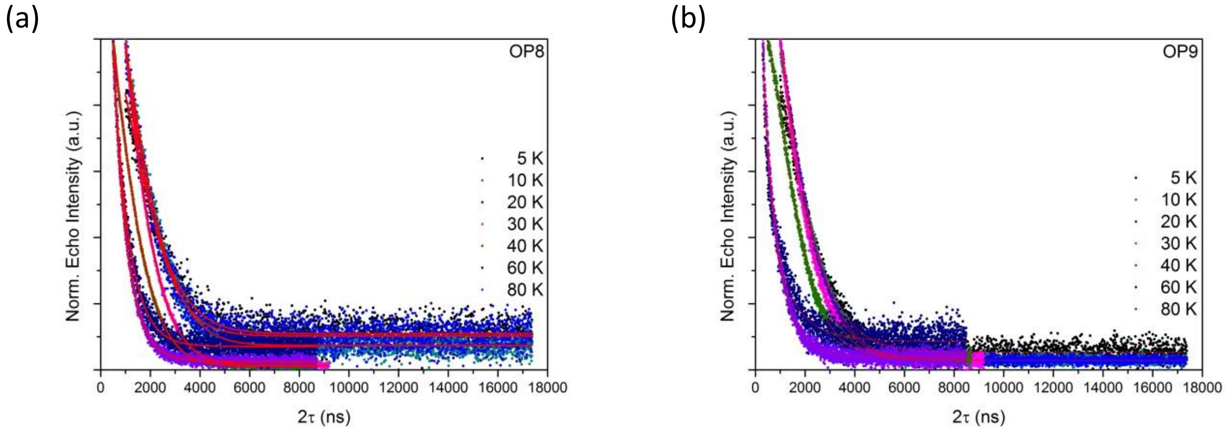

Figure S20. Normalized echo integral of **1** as a function of 2τ measured at (a) OP8 and (b) OP9 (Figure S6), at the indicated temperatures. The red lines represent the best fits to equation (2), with the parameters in Table S4.

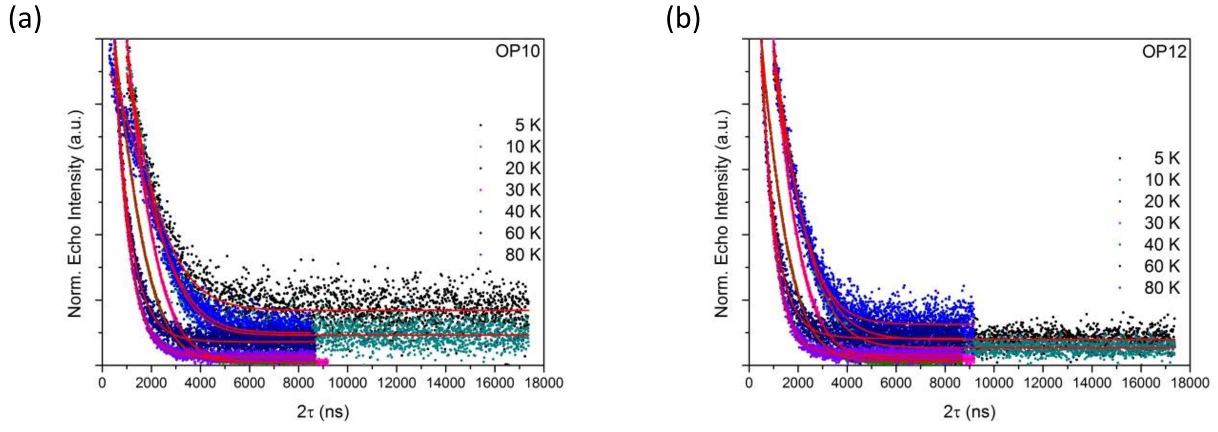

Figure S21. Normalized echo integral of **1** as a function of 2τ measured at (a) OP10 and (b) OP12 (Figure S6), at the indicated temperatures. The red lines represent the best fits to equation (2), with the parameters in Table S4

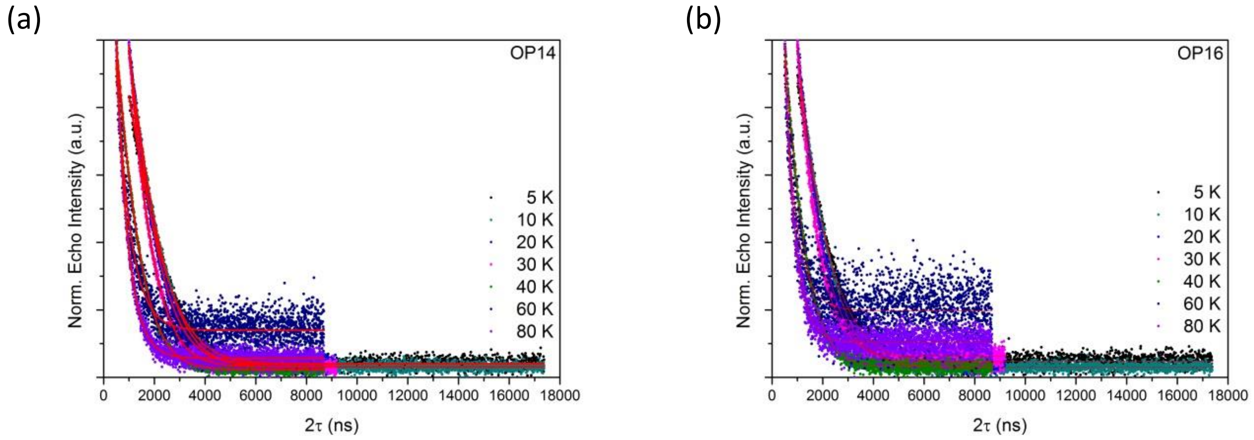

Figure S22. Normalized echo integral of **1** as a function of 2τ measured at (a) OP14 and (b) OP16 (Figure S6), at the indicated temperatures. The red lines represent the best fits to equation (2), with the parameters in Table S4.

Table S4. Extracted phase memory time (T_m) for **1** in μs (10 mM MeTHF) at X-band, based on Figures S18–S22.

T (K)	OP1	OP2	OP3	0P4	OP5	OP6	OP7	OP8
5	1.839	1.903	1.883	1.886	1.944	1.979	2.012	2.016
10	1.703	1.715	1.757	1.789	1.85	1.879	1.953	1.964
20	1.497	1.482	1.537	1.573	1.664	1.706	1.794	1.743
30		1.176		1.283		1.429	1.483	1.529
40		1.013		1.108		1.248	1.537	1.407
60		0.484		0.474		0.569	0.481	0.628
80		0.393		0.436		0.463	0.4	0.54

T (K)	OP9	OP10	OP11	OP12	OP13	OP14	OP15	OP16
5	2.041	1.899	2.01	1.93	1.912	1.905	1.807	1.822
10	2	1.999	1.962	1.906	1.808	1.778	1.754	1.683
20	1.884	1.738	1.793	1.72	1.624	1.555	1.457	1.394
30	1.645	1.558		1.411		1.236		1.119
40	1.71	1.45		1.292		1.113		0.951
60	0.43	0.629		0.552		0.572		0.388
80	0.477	0.576		0.513		0.447		0.359

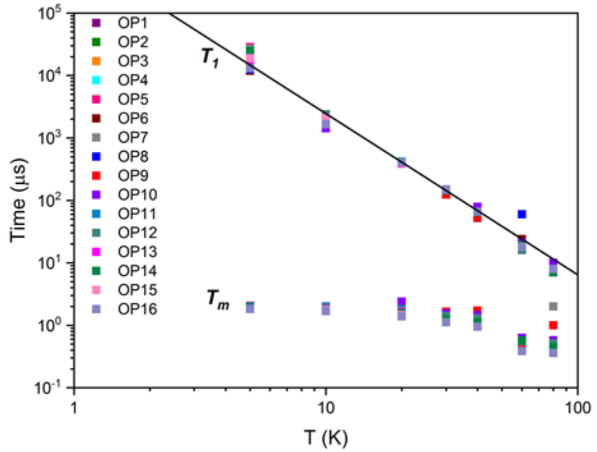

Figure S23. Temperature dependence of T_1 and T_m for **1** at X-band (9.7 GHz) and at different observer positions, as indicated in the Figure S6. The solid line corresponds to the best fit of the T_1 data with the following equation $T_1^{-1} = CT^n$ with the Raman parameters of C_1 = 1.07(4) $\cdot 10^{-6}$ μs^{-1} and n_1 = 2.58(5).

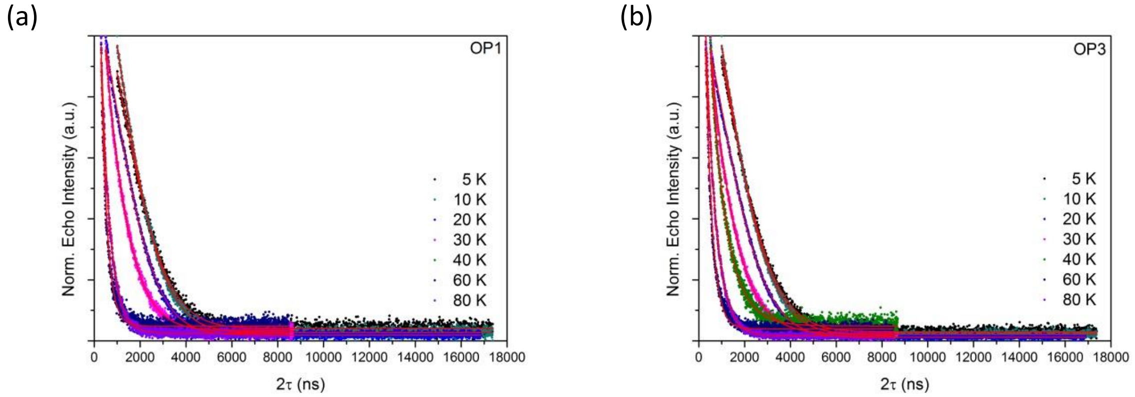

Figure S24. Normalized echo integral of **2** as a function of 2τ measured at (a) OP1and (b) OP3 (Figure S7), at the indicated temperatures. The red lines represent the best fits to equation (2), with the parameters in Table S5.

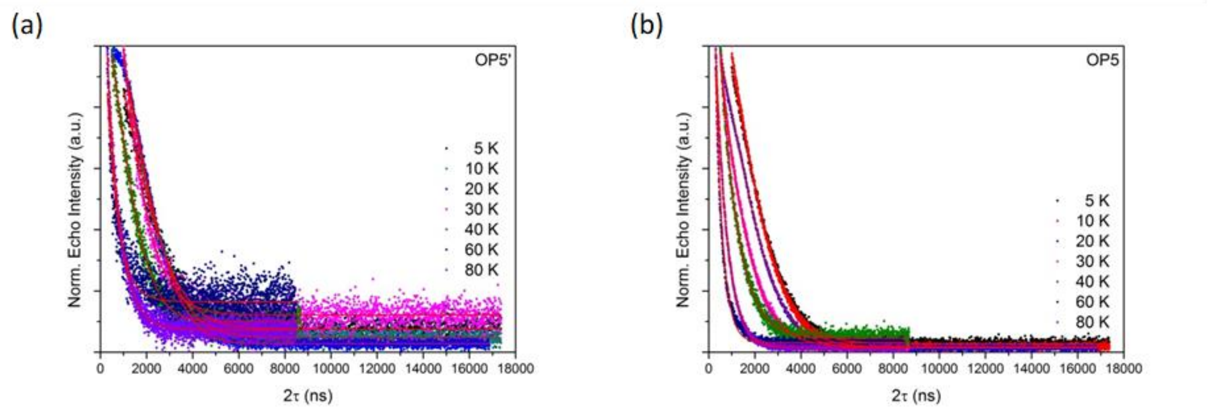

Figure S25. Normalized echo integral of **2** as a function of 2τ measured at (a) OP5'and (b) OP5 (Figure S7), at the indicated temperatures. The red lines represent the best fits to equation (2), with the parameters in Table S5.

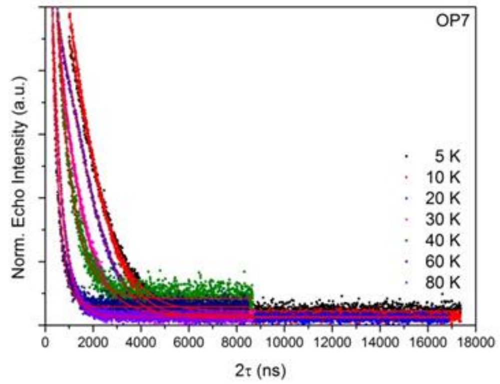

Figure S26. Normalized echo integral of **2** as a function of 2τ measured at OP7 (Figure S7), at the indicated temperatures. The red lines represent the best fits to equation (2), with the parameters in Table S5.

Table S5. Extracted phase memory time constants (T_m) for **2** in μs (10 mM MeTHF) at X-band (9.7 GHz), based on Figures S24–S26.

T (K)	OP1	OP3	OP5’	OP5	OP7
5	2.144	2.255	2.218	2.243	2.100
10	2.021	2.136	2.145	2.131	1.998
20	1.854	2.003	2.361	2.099	1.947
30	1.104	1.201	1.381	1.375	1.156
40	0.623	0.659	1.429	0.811	0.594
60	0.269	0.293	0.492	0.316	0.284
80	0.374	0.468	0.555	0.519	0.407

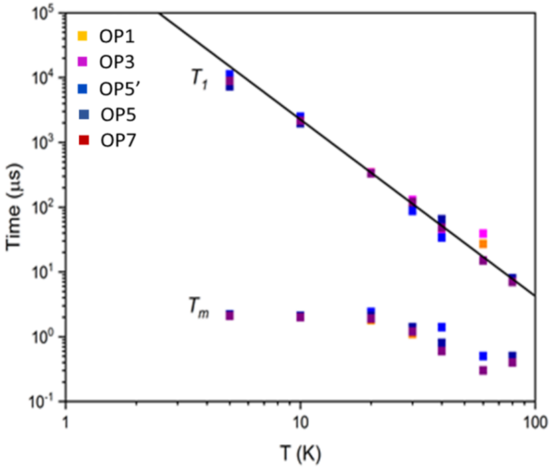

Figure S27. Temperature dependence of T_1 and T_m for **2** at X-band (9.7 GHz) and at different observer positions (Figure S7). The solid line corresponds to the best fit of the T_1 data with the following equation $T_1^{-1} = CT^n$ with the Raman parameters of C_2 = 8.73(2) $\cdot 10^{-7}$ μs^{-1} and n_2 = 2.71(2).

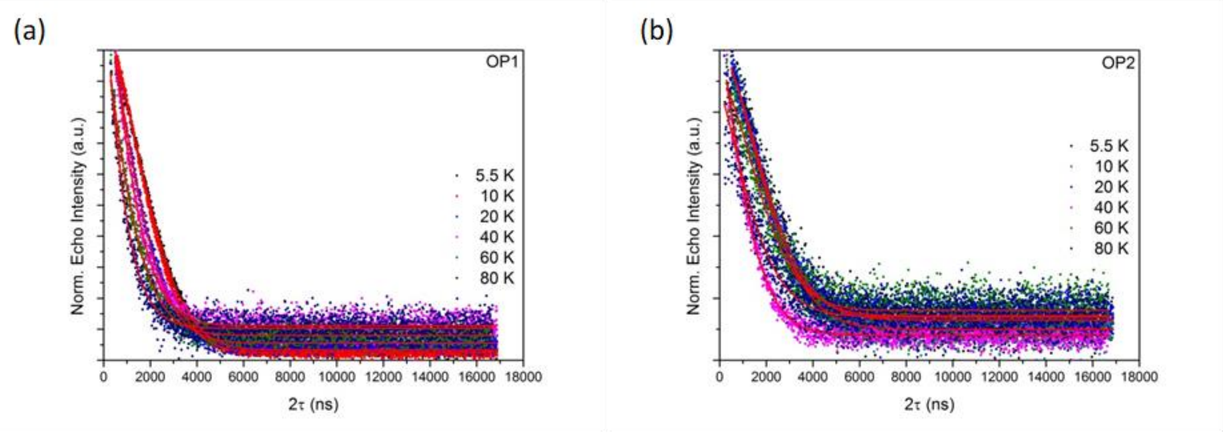

Figure S28. Normalized echo integral of **3** as a function of 2τ measured at (a) OP1and (b) OP2 (Figure S8), at the indicated temperatures. The red lines represent the best fits to equation (2), with the parameters in Table S6.

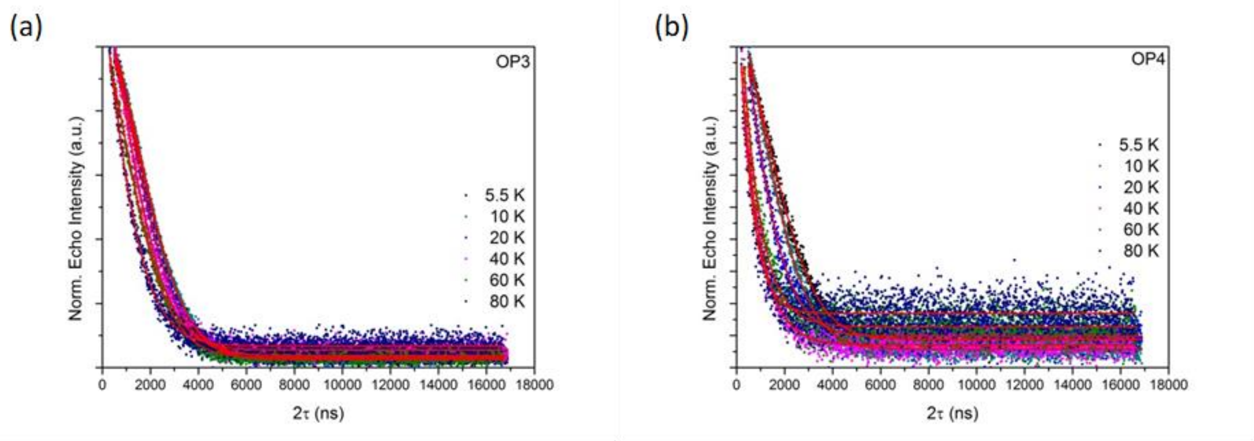

Figure S29. Normalized echo integral of **3** as a function of 2τ measured at (a) OP3 and (b) OP4 (Figure S8), at the indicated temperatures. The red lines represent the best fits to equation (2), with the parameters in Table S6.

Table S6. Extracted phase memory time constants (T_m) for **3** in μs (10 mM MeTHF) at X-band (9.7 GHz), based on Figures S28–S29.

T (K)	OP1	OP2	OP3	OP4
5.5	2.377	2.514	2.439	2.295
10	2.217	2.476	2.367	1.985
20	1.793	2.313	2.135	1.441
40	1.334	2.218	1.842	0.799
60	1.25	2.229	1.87	0.78
80	0.888	1.697	1.307	0.57

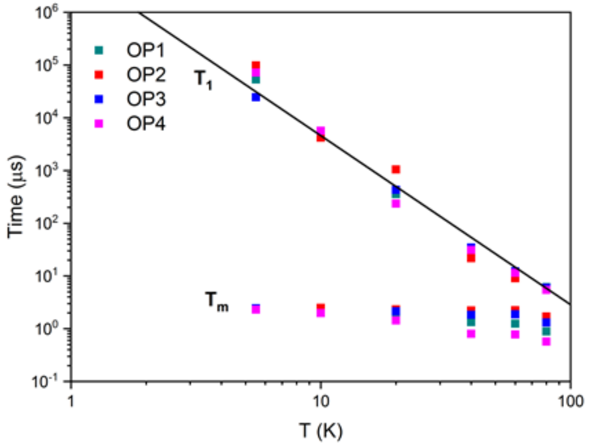

Figure S30. Temperature dependence of T_1 and T_m for **3** at X-band (9.7 GHz) and at different observer positions (Figure S8). The solid line corresponds to the best fit of the T_1 data using the equation $T_1^{-1} = CT^n$ with the Raman parameters of C_3 = 1.23(7) x 10^{-7} μs^{-1} and n_3 = 3.22(7).

2.5 Carr-Purcell-Meiboom-Gill (CPMG) experiments

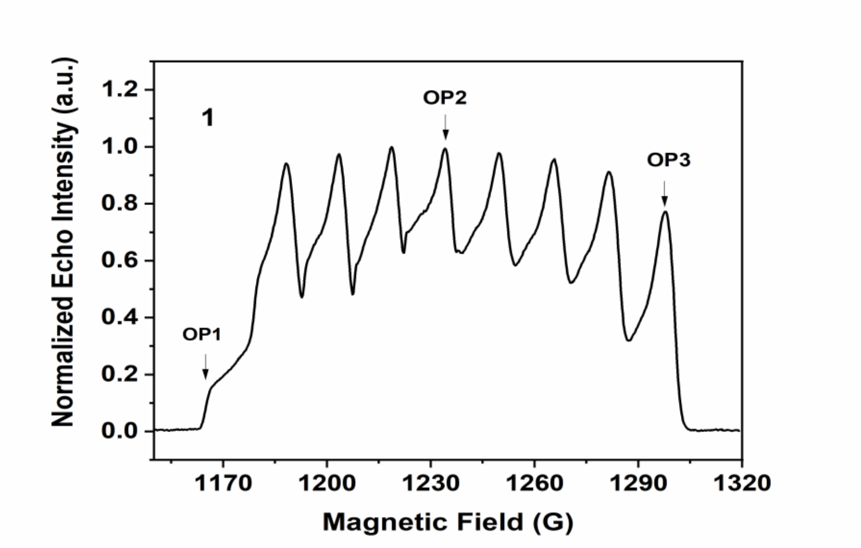

Figure S31. EDFS spectrum at Q-band (34 GHz) of **1** in MeTHF (10 mM) at 20 K. The arrows mark the observer positions at which the CPMG data in Table S7 were recorded.

Table S7. Extracted CPMG coherence times (T_{CPMG}) for **1** in MeTHF (10 mM) at 3 and 5 K, at Q-band (34 GHz) and at observer positions OP1-OP3 (Figure S31).

	**OP1**		**OP2**		**OP3**	
Temperature	$T_{CPMG,s}$ (μs)	$T_{CPMG,f}$ (μs)	$T_{CPMG,s}$ (μs)	$T_{CPMG,f}$ (μs)	$T_{CPMG,s}$ (μs)	$T_{CPMG,f}$ (μs)
3 K	40.5(5)	8.6(2)	50(1)	19.4(2)	41(1)	9.0(4)
5 K	35.5(4)	4.2(1)			24.7(4)	3.4(1)

Note: the subscripts ***s*** and ***f*** stand for the slow and fast (T_{SD}) components in the bi-exponential model.

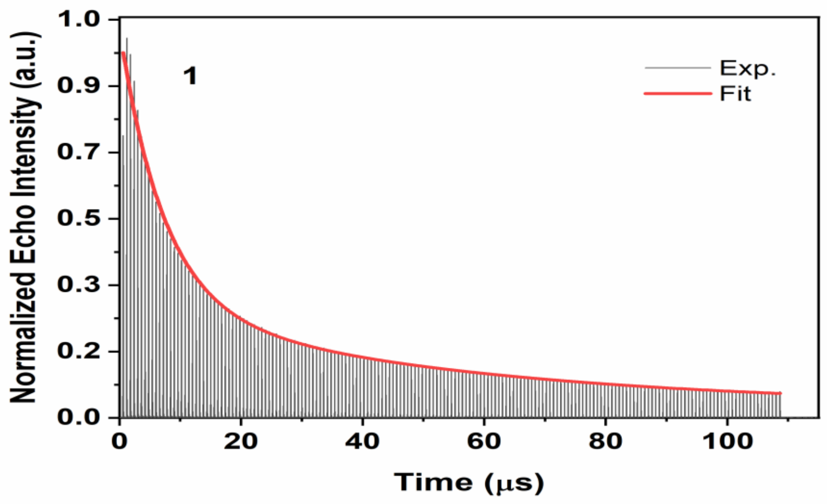

Figure S32. Echo signal decay recorded with a CPMG sequence for **1** in MeTHF (10 mM) at 3 K, at OP2 (Figure S31). The red line is an exponential fit yielding T_{CPMG} = 50(1) μs.

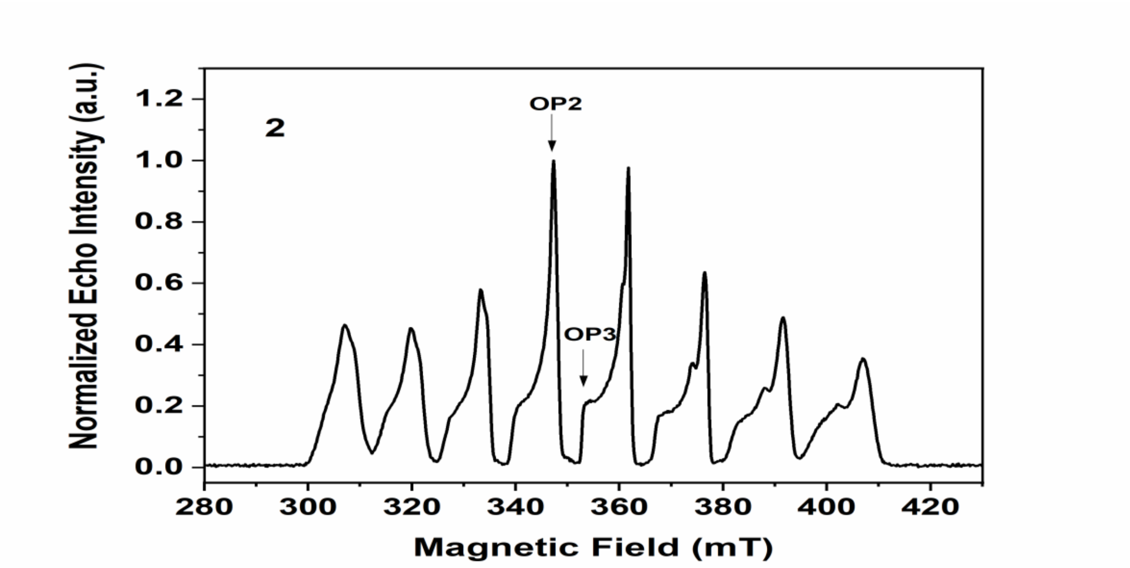

Figure S33. EDFS spectrum at X-band (9.7 GHz) of **2** in MeTHF (10 mM) at 5 K. The arrows mark the observer positions at which CPMG data in Table S8 were recorded.

Table S8. Extracted CPMG coherence times (T_{CPMG}) for **2** in MeTHF (10 mM) at X-band (9.7 GHz) and 5 K, at the observer positions marked in Figure S33.

	**OP2**		**OP3**	
Temperature	$T_{CPMG,\,s}$ (μs)	$T_{CPMG,\,f}$ (μs)	$T_{CPMG,\,s}$ (μs)	$T_{CPMG,\,f}$ (μs)
5 K	18.6(4)	3.1(1)	30(1)	5.0(2)

Note: the subscripts ***s*** and ***f*** stand for the slow and fast (T_{SD}) components in the bi-exponential model.

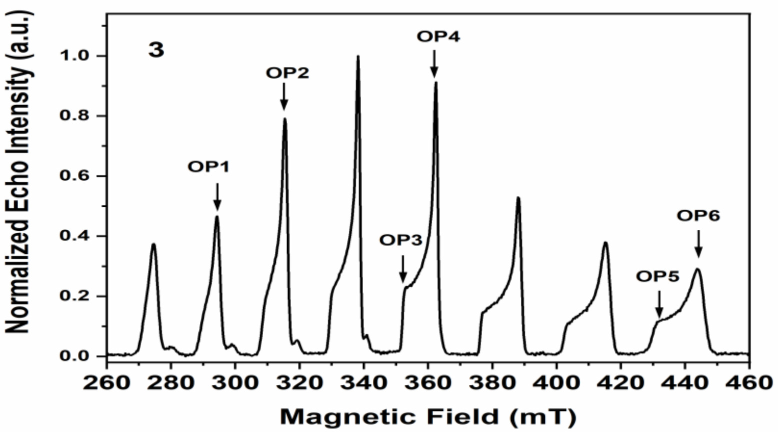

Figure S34. EDFS spectrum at X-band (9.7 GHz) for **3** in MeTHF (10 mM) at 20 K. The arrows mark the observer positions at which the CPMG data in Table S9 were recorded.

Table S9. Extracted spin-coherence times (T_{CPMG}) for **3** in MeTHF (10 mM) at X-band (9.7 GHz) and temperatures from 5.5 to 80 K, at the observer positions marked in Figure S34.

	**OP1**		**OP3**		**OP4**	
Temperature (K)	$T_{CPMG,s}$ (μs)	$T_{CPMG,f}$ (μs)	$T_{CPMG,s}$ (μs)	$T_{CPMG,f}$ (μs)	$T_{CPMG,s}$ (μs)	$T_{CPMG,f}$ (μs)
**5.5 K**	77(2)	8.8(6)	161(4)	9.0(9)	71.0(7)	6.7(3)
10 K	55(1)	10.8(3)	112(3)	6.9(7)	59.0(7)	7.1(3)
20 K	25(2)	7.1(2)	58(2)	8.5(5)	33.6(3)	5.7(1)
40 K	2.85(6)	0	14(2)	3.9(1)	10.3(2)	2.0(1)
60 K	2.4(3)	0	9(1)	0	4.46(8)	0
80 K			3.9(2)	0	3.6(3)	0
	**OP5**		**OP6**			
5.5 K	83(2)	12.0(5)	43.3(5)	5.6(2)		

10 K	46(2)	9.3(5)	25.9(3)	4(1)		
20 K			9.8(4)	2.6(1)		
40 K			4.2(2)	1.7(1)		
60 K			1.9(2)	0		
80 K			1.0(1)	0		

Note: the subscripts ***s*** and ***f*** stand for the slow and fast (T_{SD}) components in the bi-exponential model.

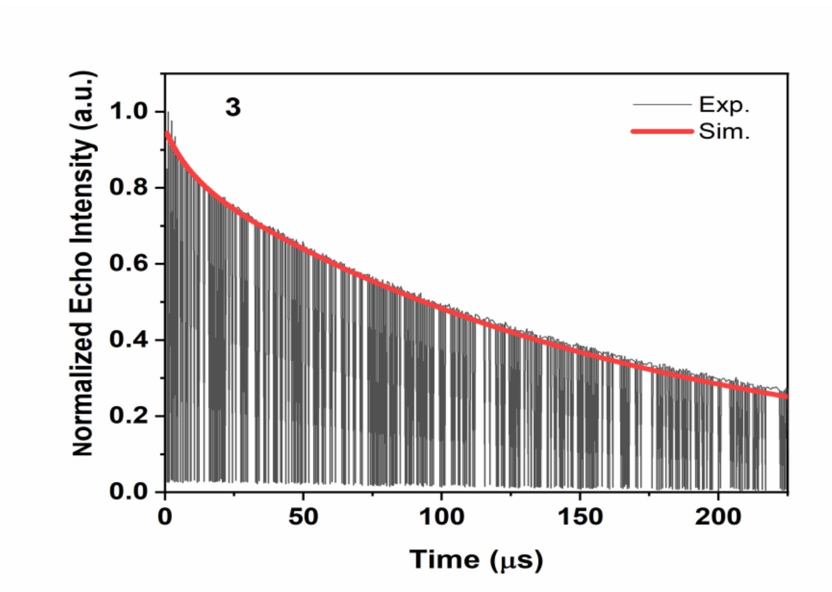

Figure S35. Echo signal decay recorded with a CPMG sequence for **3** in MeTHF (10 mM) at 5.5 K, at OP3 (Figure S34). The red line is an exponential fit yielding T_2^{dd} ($T_{CPMG,s}$) = 161(4) μs (Table S9).

2.6 Transient Nutation Experiments

In the transient nutation experiments, a nutation pulse, t_p, rotated the magnetization through an angle $\theta = \mu_B B_1 t_p/\hbar$, followed by a two-pulse Hahn-echo detection sequence t_p-t_w-$\pi/2$-τ-π-τ-*echo*, with fixed τ = 200 ns and t_w = 6000 ns that is chosen to be much longer than T_m time. The Rabi frequency, Ω_R, was determined by zero-filling the Rabi oscillation curves followed by a fast Fourier transform (FFT).

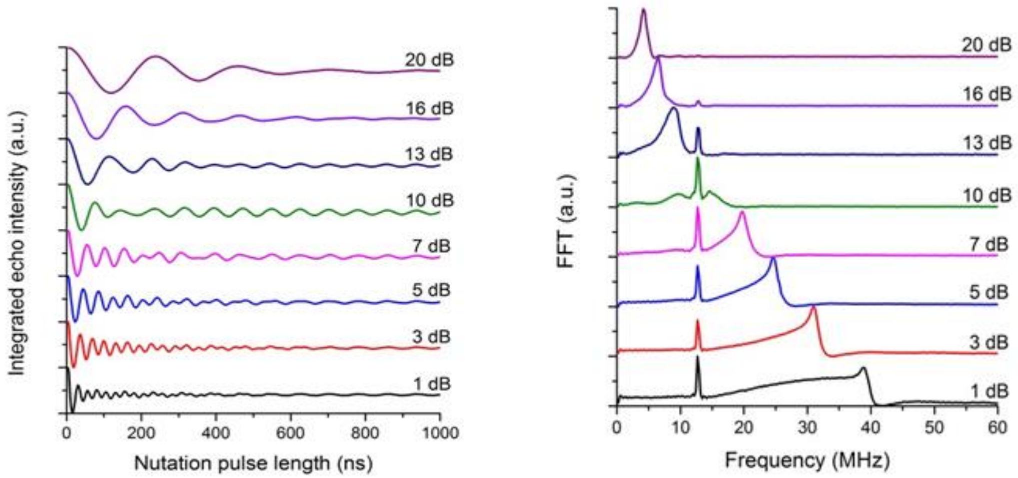

Figure S36. (left) Rabi oscillations for **1** at OP2 (Figure S6) and at 20 K, acquired at different microwave attenuations, and (right) the corresponding Fourier transforms.

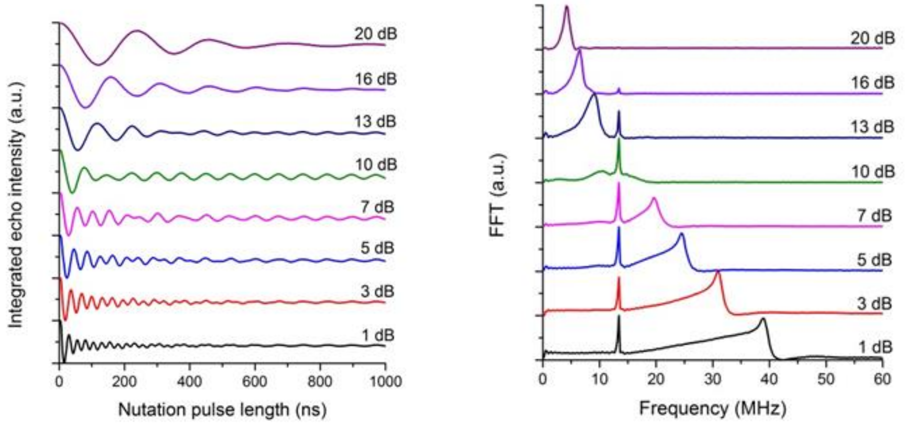

Figure S37. (left) Rabi oscillations for **1** at OP4 (Figure S6) and at 20 K, acquired at different microwave attenuations, and (right) the corresponding Fourier transforms.

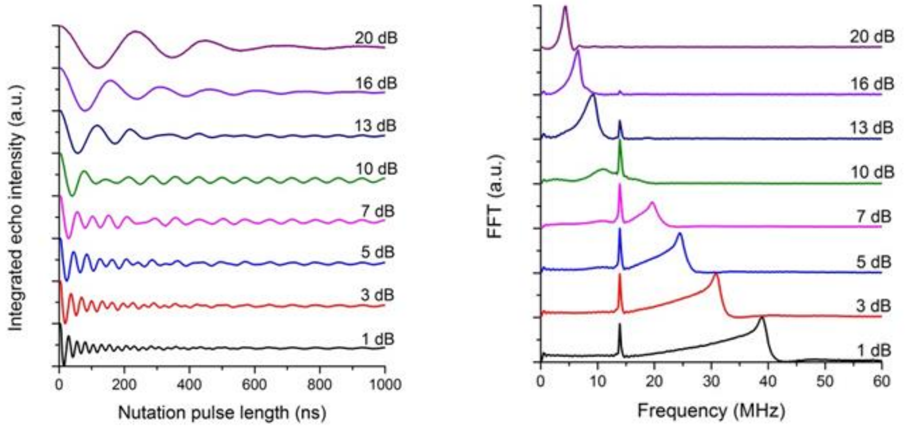

Figure S38. (left) Rabi oscillations for **1** at OP6 (Figure S4) and at 20 K, acquired at different microwave attenuations, and (right) the corresponding Fourier transforms.

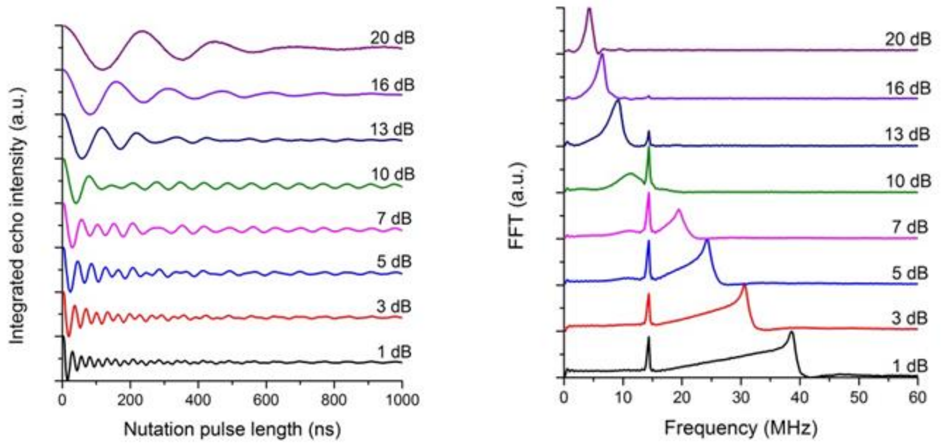

Figure S39. (left) Rabi oscillations for **1** at OP7 (Figure S6) and at 20 K, acquired at different microwave attenuations, and (right) the corresponding Fourier transforms.

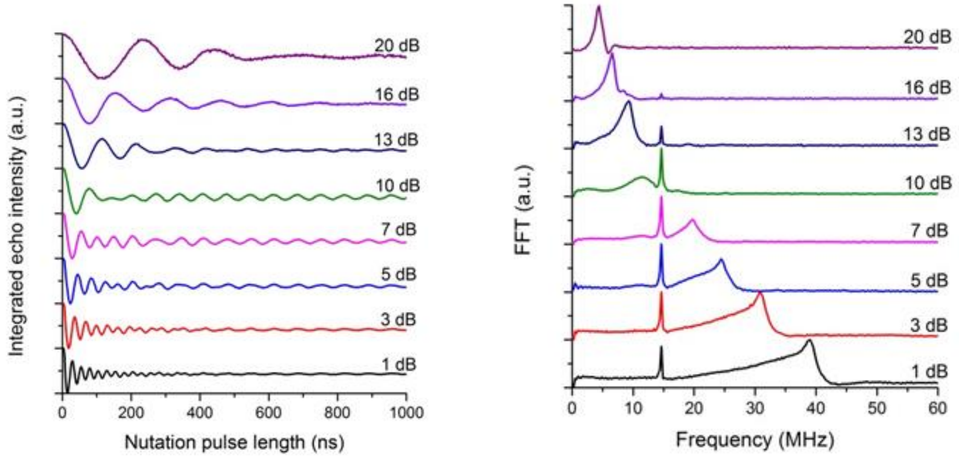

Figure S40. (left) Rabi oscillations for **1** at OP8 (Figure S6) and at 20 K, acquired at different microwave attenuations, and (right) the corresponding Fourier transforms.

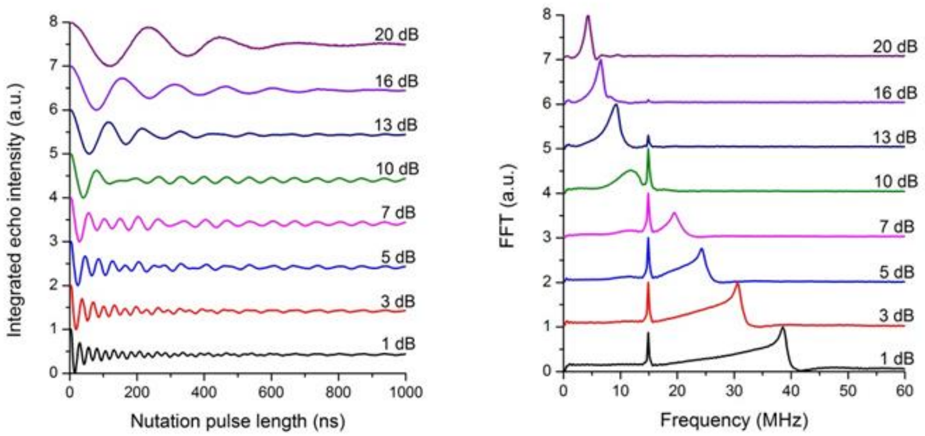

Figure S41. (left) Rabi oscillations for **1** at OP9 (Figure S6) and at 20 K, acquired at different microwave attenuations, and (right) the corresponding Fourier transforms.

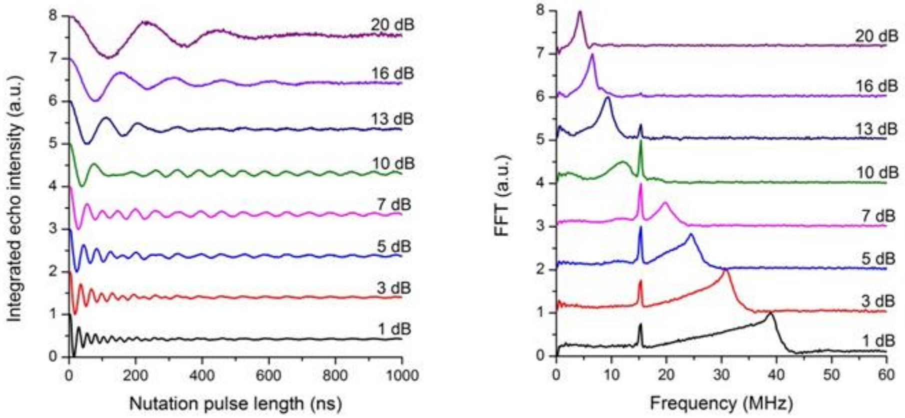

Figure S42. (left) Rabi oscillations for **1** at OP10 (Figure S6) and at 20 K, acquired at different microwave attenuations, and (right) the corresponding Fourier transforms

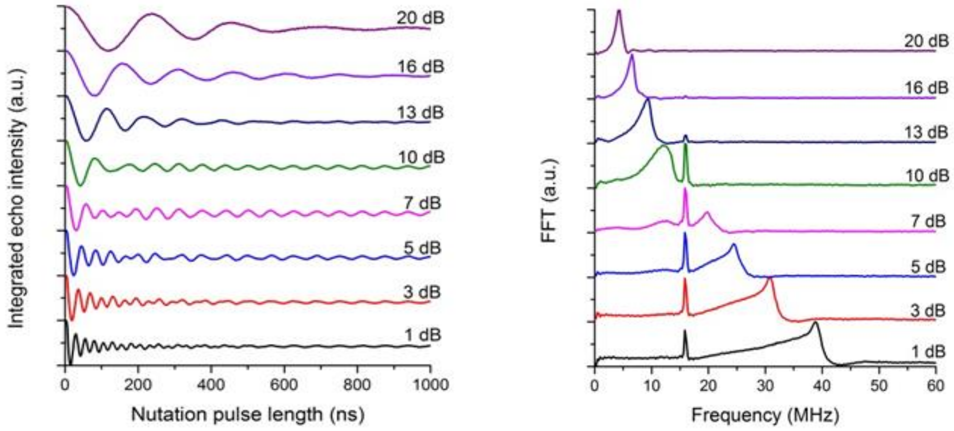

Figure S43. (left) Rabi oscillations for **1** at OP12 (Figure S6) and at 20 K, acquired at different microwave attenuations, and (right) the corresponding Fourier transforms.

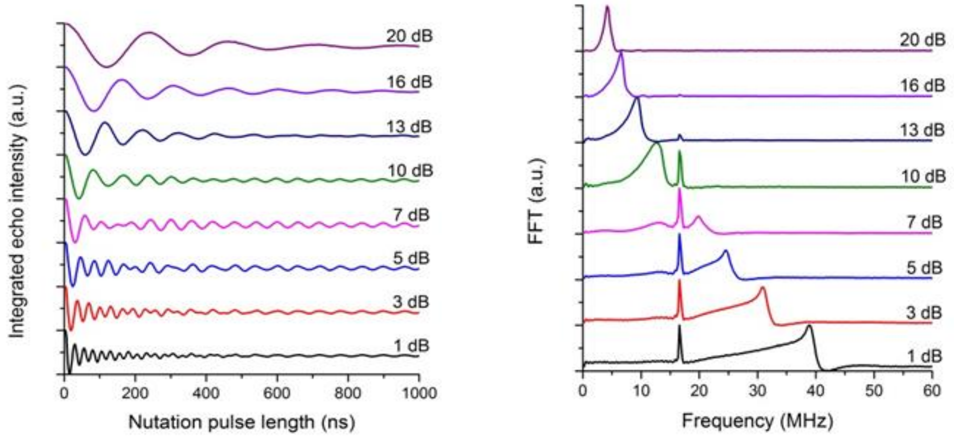

Figure S44. (left) Rabi oscillations for **1** at OP14 (Figure S6) and at 20 K, acquired at different microwave attenuations, and (right) the corresponding Fourier transforms.

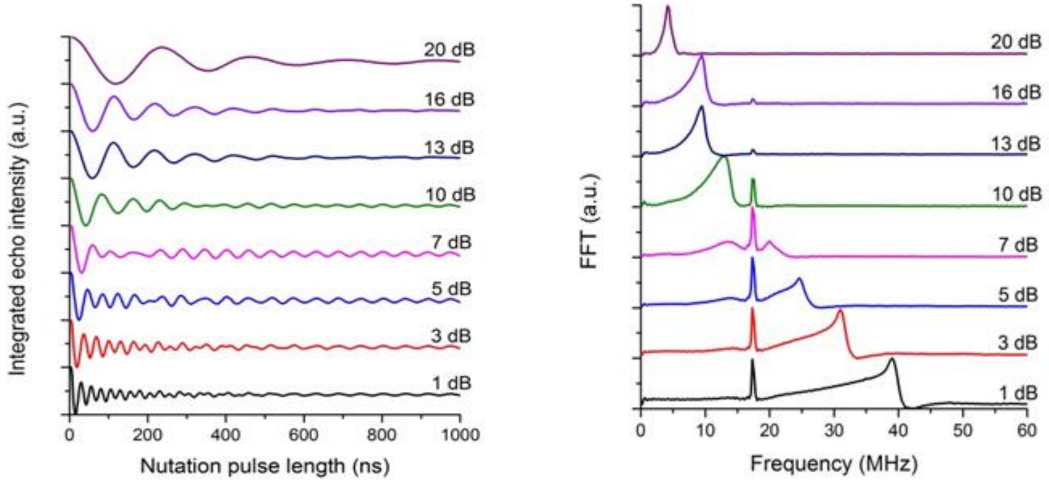

Figure S45. (left) Rabi oscillations for **1** at OP16 (Figure S6) and at 20 K, acquired at different microwave attenuations, and (right) the corresponding Fourier transforms.

Table S10. Extracted Rabi frequencies for **1** (10 mM MeTHF), at 20 K.

Attenuation (dB)	Relative B_1 (a.u.)	OP2	OP4	OP6	OP7	OP8	OP9	OP10	OP12	OP14	OP16
1	5.62	38.87	38.87	38.92	38.59	38.95	38.54	38.88	38.81	38.91	39.03
3	4.47	30.99	30.93	30.77	30.61	30.87	30.59	30.75	30.77	30.91	31
5	3.55	24.64	24.51	24.48	24.28	24.46	24.29	24.48	24.45	24.52	24.59
7	2.82	19.75	19.67	19.62	19.48	19.76	19.46	19.78	19.83	19.88	19.97
10	1.99	14.55	10.56	11.23	11.53	11.86	12.04	12.29	12.08	12.65	12.9
13	1.41	9.02	9.09	9.12	9.15	9.29	9.21	9.33	9.34	9.28	9.49
16	1	6.48	6.52	6.54	6.49	6.6	6.57	6.52	6.56	6.56	9.38
20	0.63	4.23	4.21	4.33	4.28	4.38	4.29	4.29	4.26	4.21	4.26

Table S11. Extracted Rabi frequencies for **1** (10 mM MeTHF), at 80 K.

Attenuation (dB)	Relative B1 (a.u.)	OP2	OP8	OP10	OP14
3	4.47	30.28	30.16	30.07	30.24
7	2.82	19.27	19.27	19.23	19.45
13	1.41	8.89	9.09	9.05	9.28

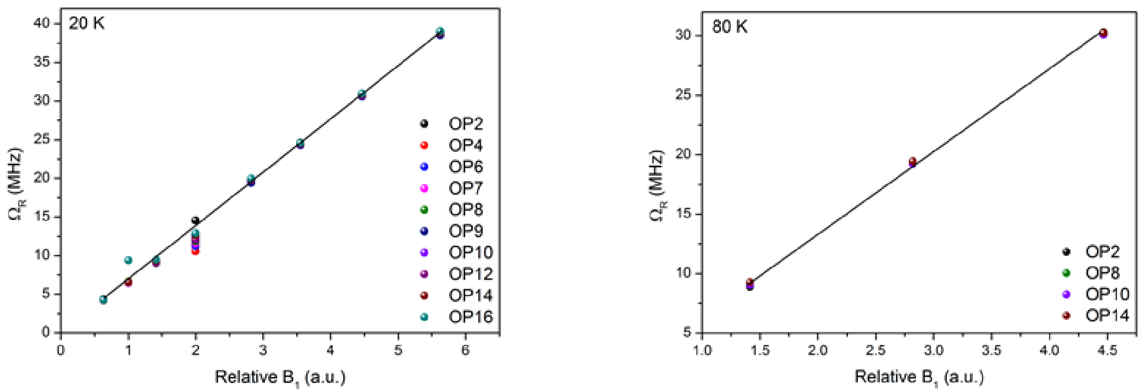

Figure S46. B_1 dependence of the Rabi frequency (Ω_R) of **1** at different observable positions (OP) and at (left) 20 K and (right) 80 K. The solid line is a guide for the eye emphasizing on the linear behaviour.

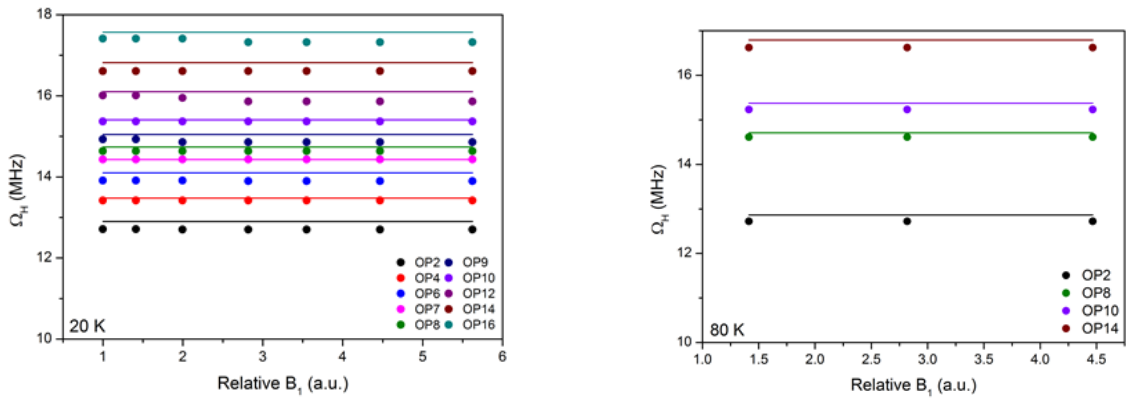

Figure S47. B_1 dependence of the ^{1}H nuclear frequency (Ω_H) of **1** at different observable positions (OP) (based on Figure S6), measured at (left) 20 K and (right) 80 K. The solid line shows the theoretical ^{1}H nuclear frequency (Ω_H) for each OP.

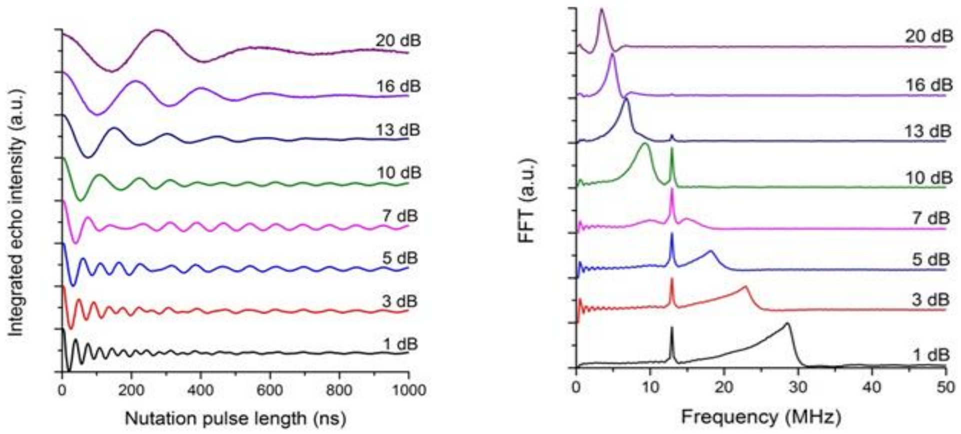

Figure S48. (left) Rabi oscillations for **2** at OP1 (Figure S7) and at 20 K, acquired at different microwave attenuations, and (right) corresponding Fourier transforms.

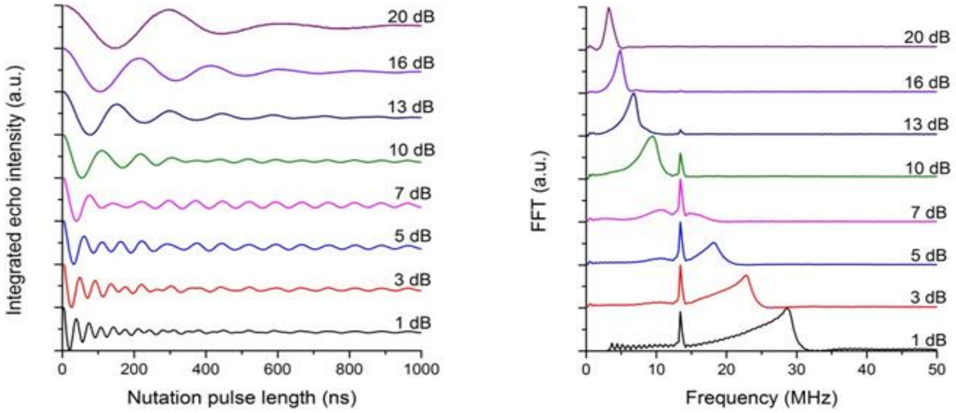

Figure S49. (left) Rabi oscillations for **2** at OP2 (Figure S7) and at 20 K, acquired at different microwave attenuations, and (right) the corresponding Fourier transforms.

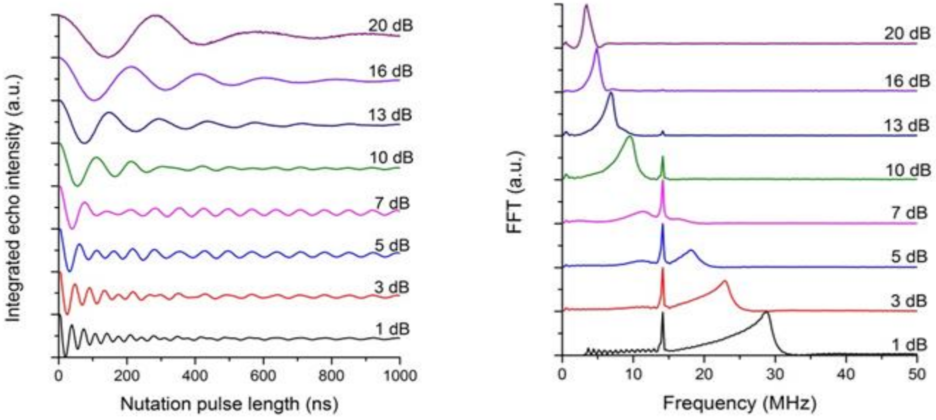

Figure S50. (left) Rabi oscillations for **2** at OP3 (Figure S7) and at 20 K, acquired at different microwave attenuations, and (right) the corresponding Fourier transforms.

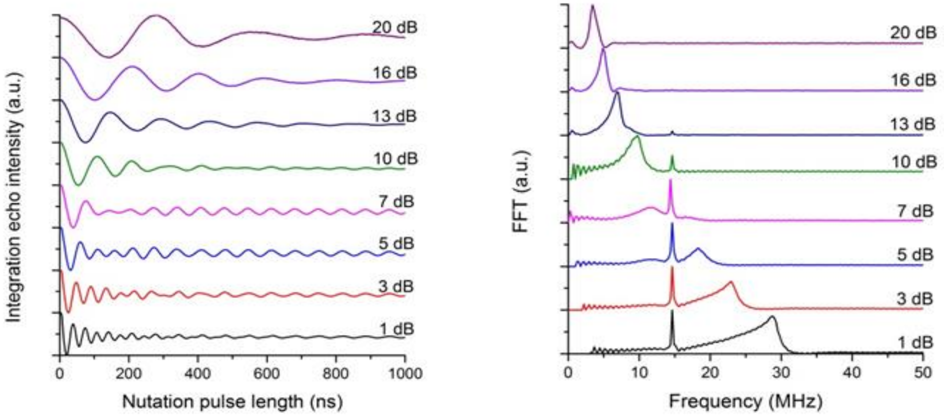

Figure S51. (left) Rabi oscillations for **2** at OP4 (Figure S7) and at 20 K, acquired at different microwave attenuations, and (right) the corresponding Fourier transforms.

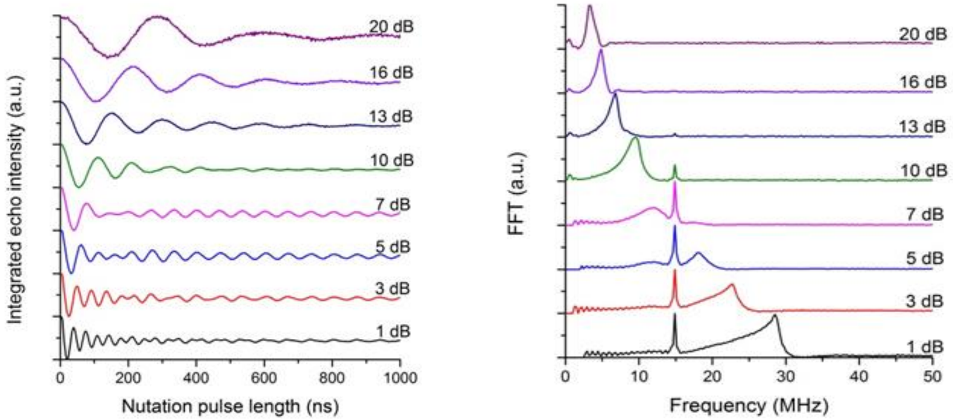

Figure S52. (left) Rabi oscillations for **2** at OP5' (Figure S7) and at 20 K, acquired at different microwave attenuations, and (right) the corresponding Fourier transforms.

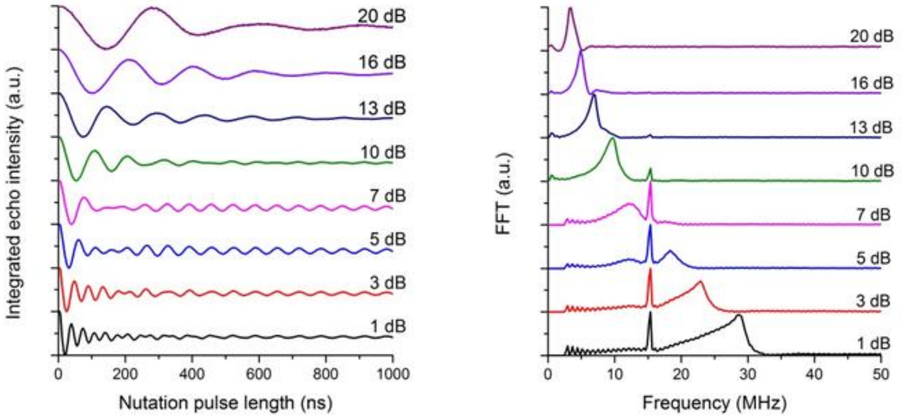

Figure S53. (left) Rabi oscillations for **2** at OP5 (Figure S7) and at 20 K, acquired at different microwave attenuations, and (right) the corresponding Fourier transforms.

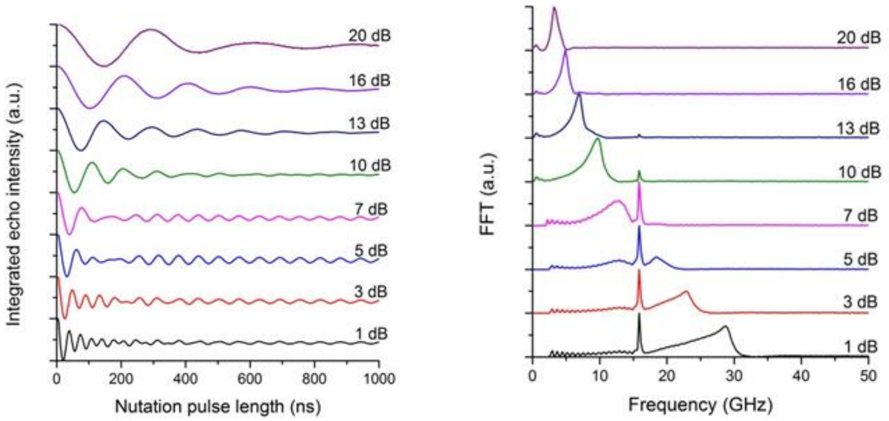

Figure S54. (left) Rabi oscillations for **2** at OP6 (Figure S7) and at 20 K, acquired at different microwave attenuations, and (right) the corresponding Fourier transforms.

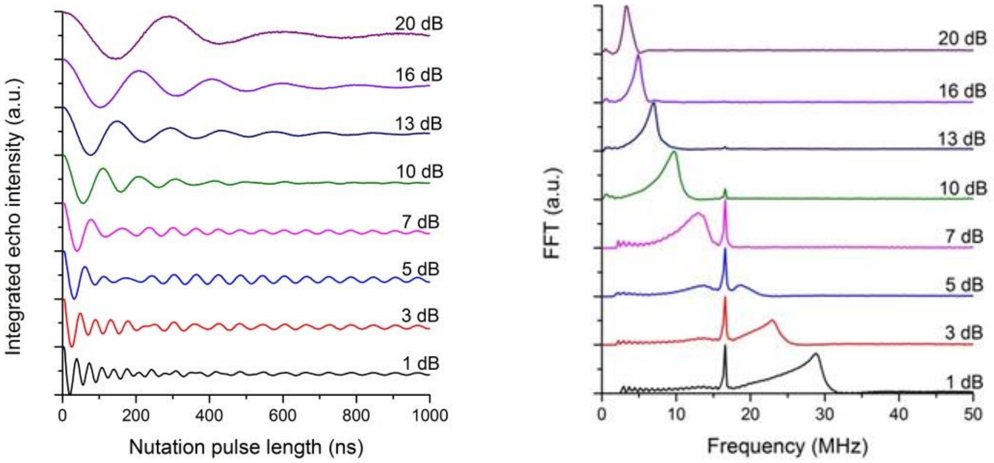

Figure S55. (left) Rabi oscillations for **2** at OP7 (Figure S7) and at 20 K, acquired at different microwave attenuations, (right) corresponding Fourier transforms.

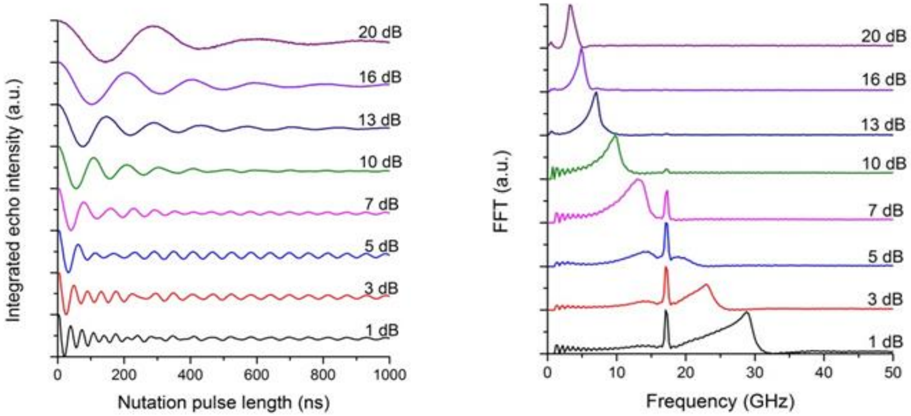

Figure S56. (left) Rabi oscillations for **2** at OP8 (Figure S7) and at 20 K, acquired at different microwave attenuations, and (right) the corresponding Fourier transforms.

Table S12. Extracted Rabi frequencies for **2** (10 mM MeTHF) at 20 K.

Attenuation (dB)	Relative B_1 (a.u.)	OP1	OP2	OP3	OP4	OP5'	OP5	OP6	OP7	OP8
1	5.62	28.56	28.65	28.73	28.8	28.54	28.61	28.71	28.81	28.82
3	4.47	22.9	22.78	22.93	22.93	22.69	22.89	22.89	22.96	22.95
5	3.55	18.18	18.17	18.18	18.31	18.08	18.31	18.42	18.65	18.93
7	2.82	15.09	10.9	11.17	12.05	12.34	12.45	12.86	12.99	13.2
10	1.99	9.34	9.45	9.51	9.75	9.56	9.69	9.67	9.74	9.77
13	1.41	6.84	6.76	6.83	6.96	6.83	6.96	6.95	6.95	6.95
16	1	4.89	4.84	4.83	5	4.86	4.88	4.87	4.92	4.9
20	0.63	3.44	3.19	3.34	3.42	3.3	3.32	3.24	3.3	3.28

Table S13. Extracted Rabi frequencies for **2** (10 mM MeTHF) at 60 K.

Attenuation (dB)	Relative B_1 (a.u.)	OP1	OP3	OP5	OP7
3	4.47	23.09	23.09	23.09	23.18
7	2.82	15.05	11.34	12.25	13.06
13	1.41	6.88	6.88	6.88	7.02

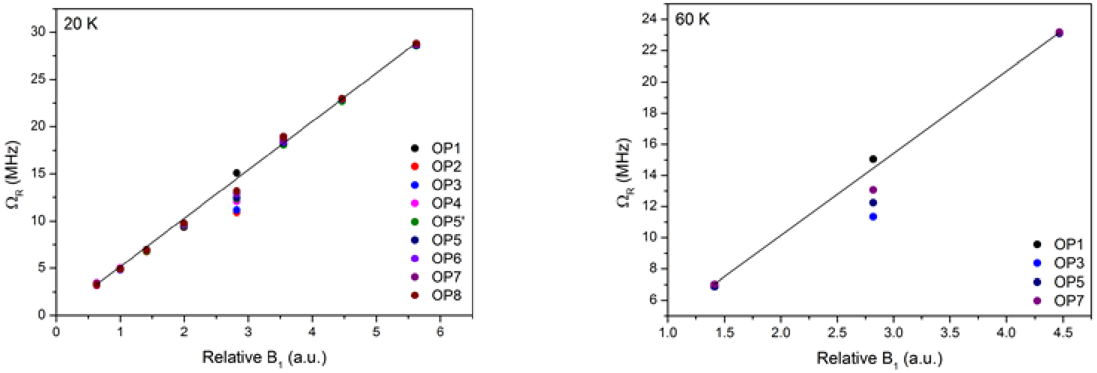

Figure S57. B_1 dependence of the Rabi frequency (Ω_R) of **2** at different observable positions (OP) (Figire S7) and at (left) 20 K and (right) 60 K. The solid line is a guide for the eye emphasizing on the linear behaviour.

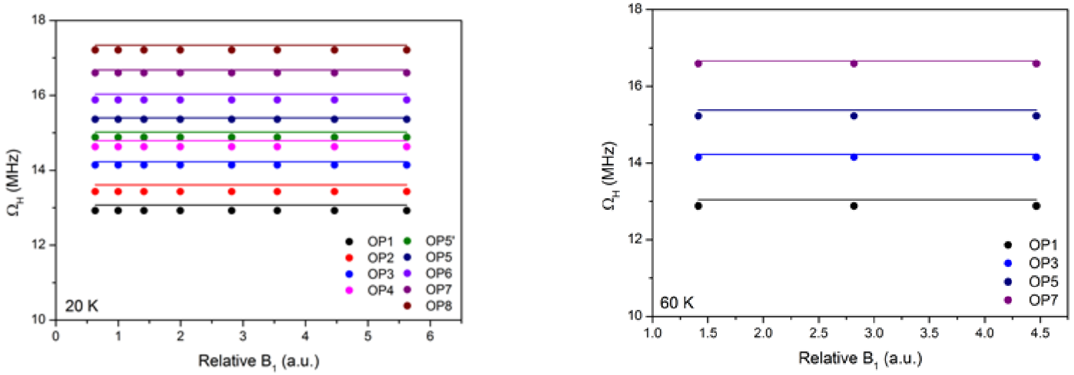

Figure S58. B_1 dependence of the ^1H nuclear frequency (Ω_H) of **2** at different observable positions (OP) (Figure S7) and at (left) 20 K and (right) 60 K. The solid line shows the theoretical ^1H nuclear frequency (Ω_H) for each OP.

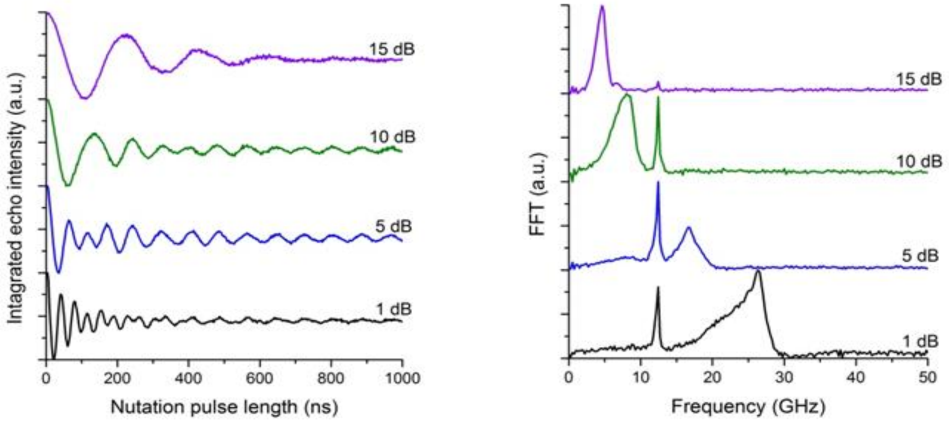

Figure S59. (left) Rabi oscillations for **3** at OP1 (Figure S8) and at 20 K, acquired at different microwave attenuations, and (right) the corresponding Fourier transforms.

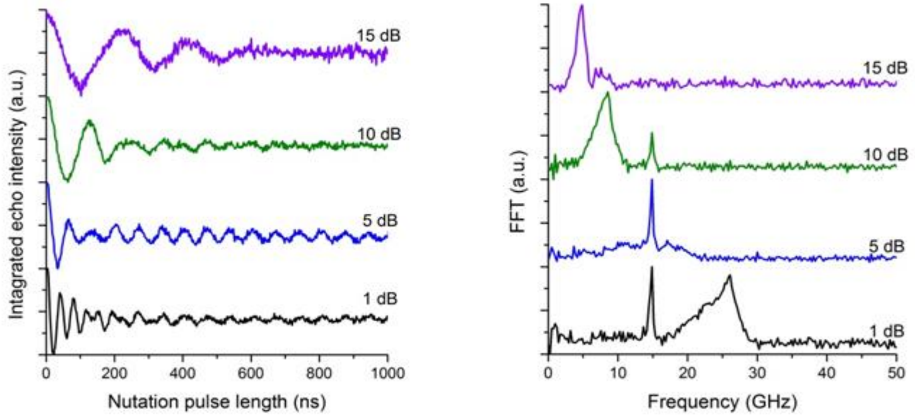

Figure S60. (left) Rabi oscillations for **3** at OP2 (Figure S8) and at 20 K, acquired at different microwave attenuations, and (right) the corresponding Fourier transforms.

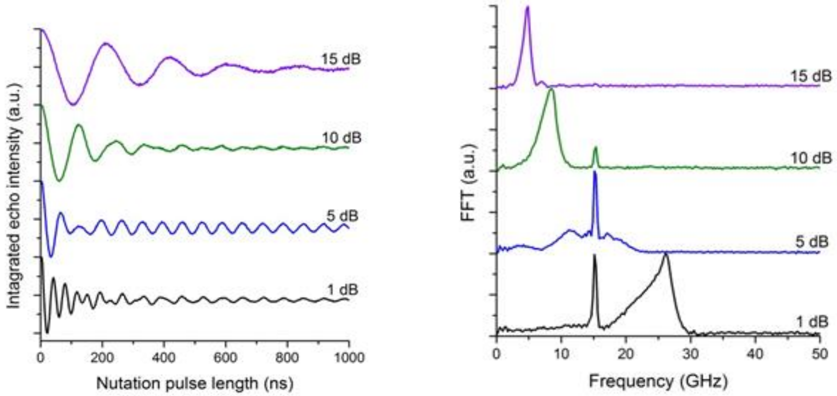

Figure S61. (left) Rabi oscillations for **3** at OP3 (Figure S8) and at 20 K, acquired at different microwave attenuations, and (right) the corresponding Fourier transforms.

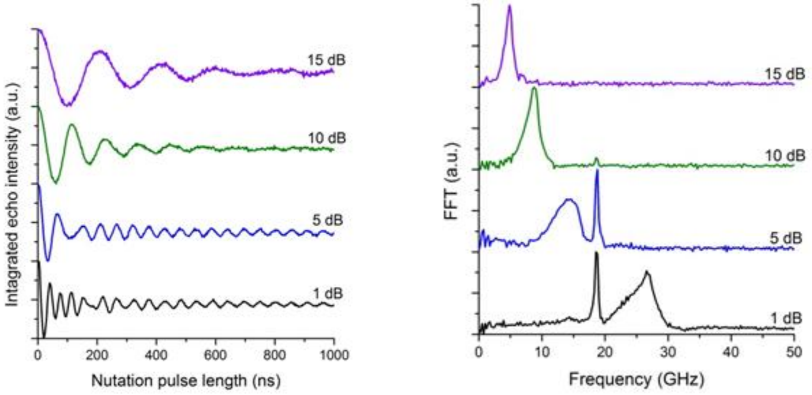

Figure S62. (left) Rabi oscillations for **3** at OP4 (Figure S8) and at 20 K, acquired at different microwave attenuations, and (right) the corresponding Fourier transforms.

Table S14. Extracted Rabi frequencies for **3** (10 mM MeTHF) at 80 K.

Attenuation (dB)	Relative B1 (a.u.)	OP1	OP3	OP5	OP7
1	5.62341	26.19	26.37	26.01	26.74
5	3.54813	17.06	16.56	17.06	14.65
10	1.99526	8.46	8.01	8.45	8.79
15	1.12202	4.77	4.64	4.77	4.87

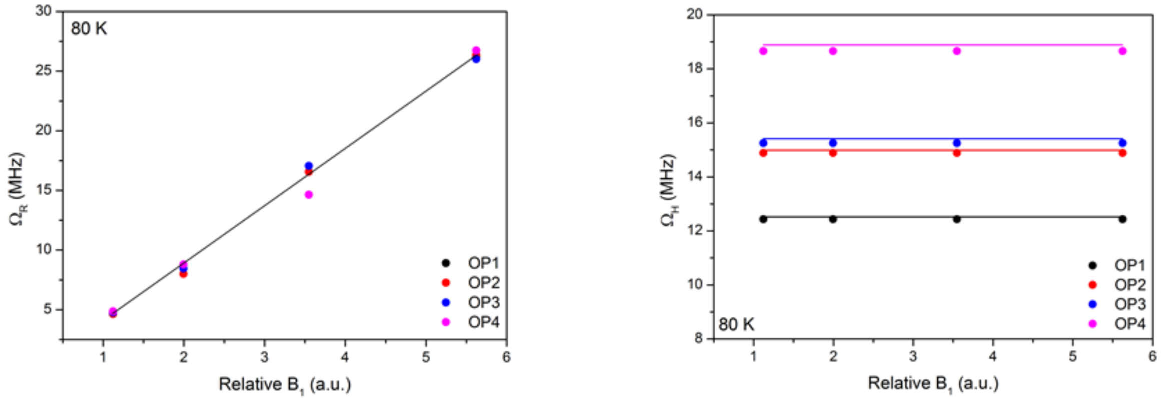

Figure S63. (left) B_1 dependence of the Rabi frequency (Ω_R) of **3** at different observable positions (OP) (Figure S8) at 80 K. The solid line is a guide for the eye emphasizing on the linear behaviour. (right) B_1 dependence of the ^{1}H nuclear frequency (Ω_H) of **3** at different applied field positions (OP) and at 80 K. The solid line shows the theoretical ^{1}H nuclear frequency (Ω_H) for each OP.

2.7 HYSCORE Spectra

HYSCORE experiments used a four-pulse electron spin-echo sequence, π/2 - τ - π/2 - t1 - π - t2 - π/2 - τ – *echo*, with π/2 and π pulse lengths of 16 and 32ns, respectively, and fixed τ time of 136 and 200 ns. The initial t_1 and t_2 times are 100 ns, increasing during the experiment with 20 ns steps up to 5200 ns; 256 points are collected in both dimensions. The absolute values were obtained after fast Fourier transformation of the obtained data in both directions, yielding to nuclear cross-peaks on a 2D (ν_1, ν_2) spectra. On the frequency domain spectra the (+,+) quadrant is described as the weak coupling regime ($2|\nu_n|>|A|$) and displays cross-peaks centred at the Larmor frequency of each nucleu, separated by the hyperfine coupling strength. Isotropic hyperfine couplings lead to ridges on the anti-diagonal of the HYSCORE spectra, while anisotropic contribution causes deviation from the anti-diagonal analogues to the magnitude of the anisotropy of the hyperfine coupling constants.

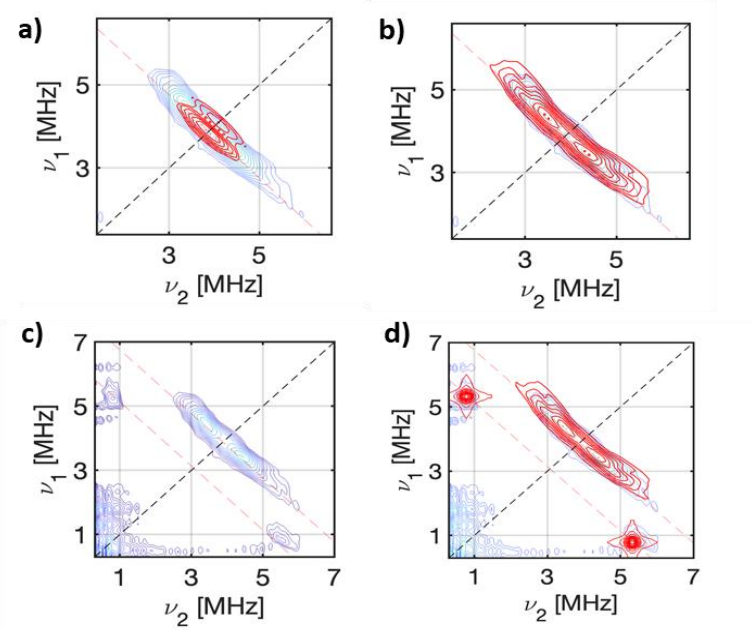

Figure S64. (a) ^{13}C HYSCORE spectrum for **1** (MeTHF) at B_0 = 360.7 mT (**OP10**; Figure S6; g_{xy}), T = 10 K and X-band (9.73 GHz) with calculation in red taking into account the point-dipole interactions and the associated spin densities at C atoms only; (b) Calculation taking into account the point-dipole interactions and the spin densities at $C^{2,5}$ and $C^{3,4}$, yielding $A_{\parallel,\perp}{}^{C2,5}$ = 7.0, 2.3 MHz and $A_{\parallel,\perp}{}^{C3,4}$ = 4.2, 0.65 MHz; (c) An expansion of the spectrum revealing ^{13}C and ^{29}Si ridges; (d) Calculation of the spectrum with the parameters in (b) and $a_{iso}(^{29}Si)$ = 4.6 MHz. The dashed-red antidiagonal line marks the ^{13}C and ^{29}Si Larmor frequencies.

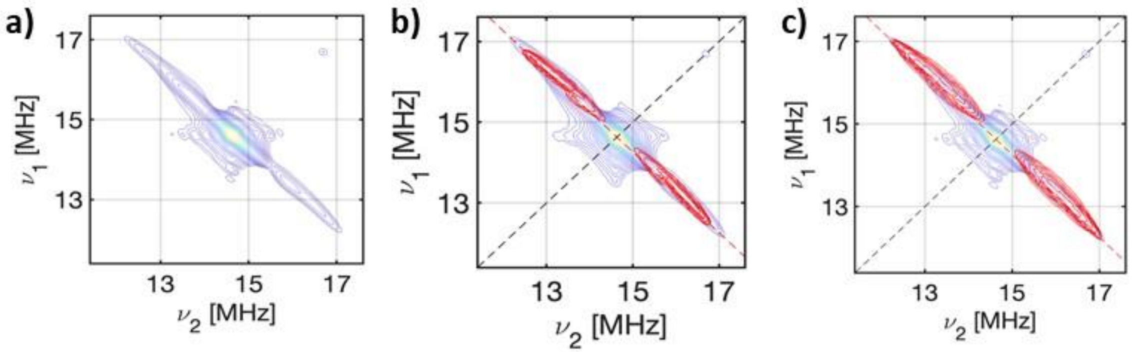

Figure S65. (a) ^{1}H HYSCORE spectrum for **1** (MeTHF) at B_0 = 345.3 mT (**OP8**; Figure S6), T = 15 K and X-band (9.734 GHz); (b) Calculation of the ^{1}H HYSCORE spectrum of **1** based on the dipole model only (see main text); (c) Calculation based on the model described in the main text using $\alpha_{isoH2,5}$ = - 1.227 MHz and $\alpha_{isoH3,4}$ = - 0.927 MHz occurring from the $A^{C2,5}$ and $A^{C3,4}$ using the McConnell model (see main text); The dashed-red antidiagonal line marks the ^{1}H Larmor frequency.

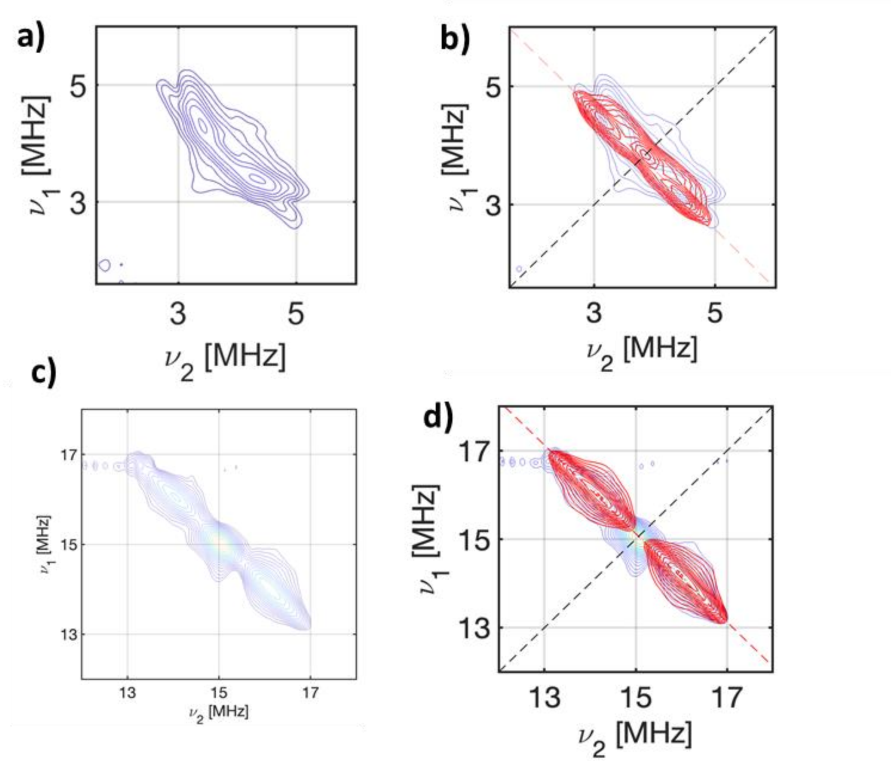

Figure S66. (a) ^{13}C HYSCORE spectrum for **1** (MeTHF) at B_0 = 353.8 mT (**OP9**; Figure S6; g_z), T = 10 K and X-band (9.734 GHz); (b) Calculation of the ^{13}C HYSCORE spectrum taking into account the point-dipole interactions and the associated spin densities at C atoms, yielding: $A_{\|,\perp}^{C1}$ = 1.38, 1.02 MHz, $A_{\|,\perp}^{C2,5}$ = 1.424, 0.267 MHz and $A_{\|,\perp}^{C3,4}$ = 2.61, 0.36 MHz; (c) ^{1}H HYSCORE spectrum for **1** (MeTHF) at B_0 = 353.8 mT (**OP9**; Figure S6), T = 10 K and X-band (9.734 GHz); (d) Calculation of the ^{1}H HYSCORE spectrum of **1** based on model described in the main text, using $\alpha_{isoH2,5}$ = - 0.302 MHz and $\alpha_{isoH3,4}$ = - 0.59 MHz, in agreement with C densities in (b). The dashed-red antidiagonal lines mark the ^{13}C (b) and ^{1}H (d) Larmor frequency.

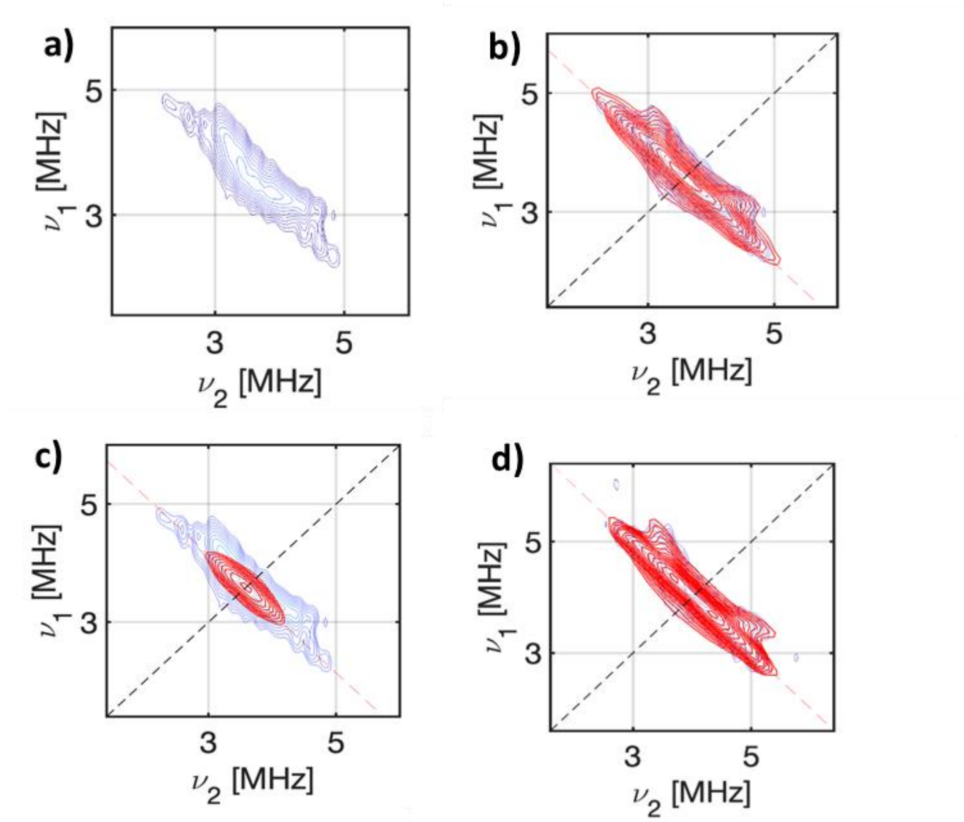

Figure S67. (a) ^{13}C HYSCORE spectrum for **2** (MeTHF) at B_0 = 333.3 mT (xy, **OP3**; Figure S7), T = 20 K and X-band (9.718 GHz); (b) Calculation of the spectrum at 333.3 mT taking into account the point-dipole interactions and the associated spin densities at C^2 and $C^{1,3}$, yielding: $A_{II,\perp}^{C2}$ = 9.0, 1.2 MHz and $A_{II,\perp}^{C1,3}$ = 0.48, -1.44 MHz. (c) Calculation of the ^{13}C HYSCORE spectrum of **2** based on the dipole model only (see main text); (d) ^{13}C HYSCORE spectrum for **2** at B_0 = 373.8 mT (xy, **OP6**; Figure S7) and its calculation (red) with the same parameters as (c). The dashed-red antidiagonal line marks the ^{13}C Larmor frequency.

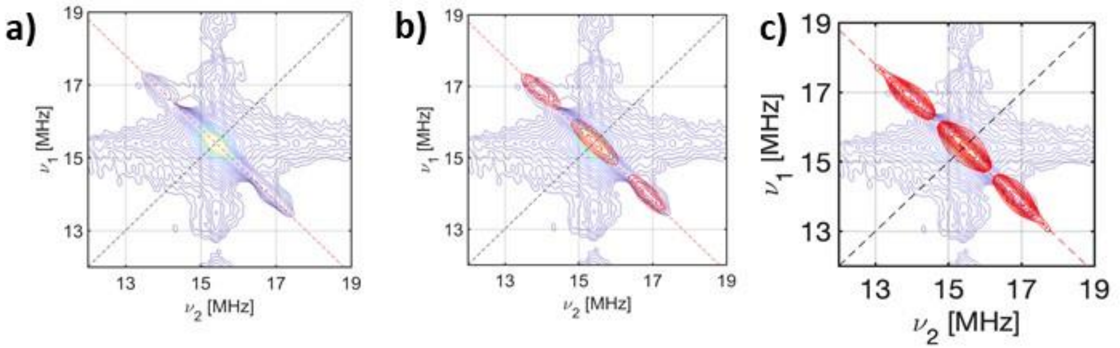

Figure S68. (a) ^{1}H HYSCORE spectrum for **2** (MeTHF) at B_0 = 361.7 mT (**OP5**; Figure S7), T = 20 K and X-band (9.72 GHz); (b) Calculation of the ^{1}H HYSCORE spectrum of **2** based on the dipole model only (see main text); (c) Calculation based on the model described in the main text using α_{isoH2} = - 2.038 and $\alpha_{isoH1,3}$ = 0, being in excellent agreement with the calculated α_{iso} values occurring from the A^{C2} and $A^{C1,3}$ using the McConnell model (see main text – Table 2).The dashed-red antidiagonal line marks the ^{1}H Larmor frequency.

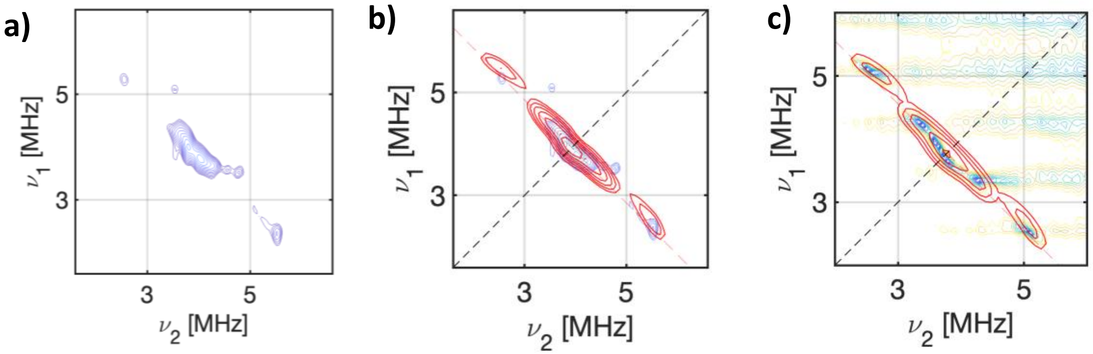

Figure S69. (a) ^{13}C HYSCORE spectrum for **2** (MeTHF) at B_0 = 366.8 mT (z, **OP6'**; Figure S7), T = 10 K and X-band (9.72 GHz); (b) Calculation of the ^{13}C HYSCORE at 366.8 mT taking into account the point-dipole interactions and the associated spin densities at C atoms, yielding: $A_{\|,\perp}{}^{C1,3}$ = 0.1, -2.34 MHz, $A_{\|,\perp}{}^{C2}$ = 4.88, 0.52 MHz and $A_{\|,\perp}{}^{C4,5}$ = 0.8, 0.03 MHz; (c) ^{13}C HYSCORE spectrum for **2** at B_0 = 352.8 mT (z, **OP5'**; Figure S7), and its calculation (red) with $A_{\|,\perp}{}^{C1,3}$ = 0.1, -1.56 MHz, $A_{\|,\perp}{}^{C2}$ = 4.5, 0.5 MHz and $A_{\|,\perp}{}^{C4,5}$ = 0.9, 0.03 MHz. The dashed-red antidiagonal line marks the ^{13}C Larmor frequency. Signal resolution is weaker in the z orientation compared to xy plane.

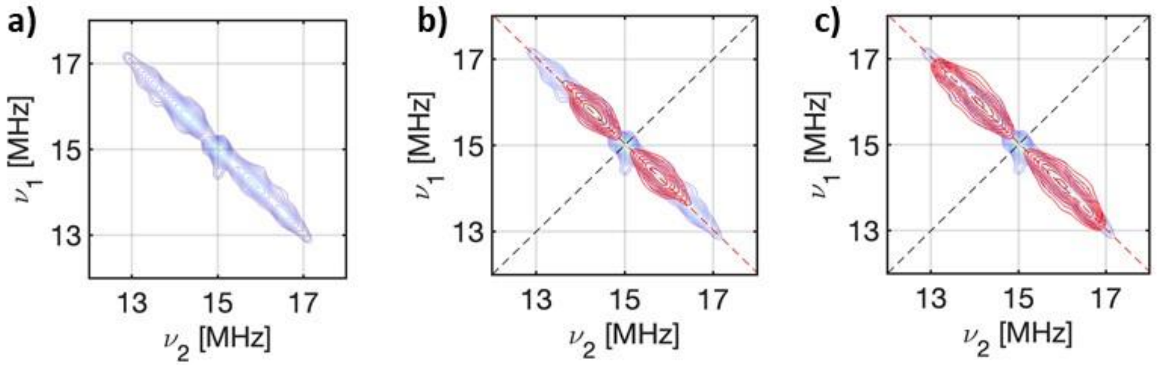

Figure S70. (a) ^{1}H HYSCORE spectrum for **2** (MeTHF) at B_0 = 352.8 mT (g_z, **OP5'**; Figure S7), T = 20 K and X-band (9.718 GHz); (b) Calculation of the ^{1}H HYSCORE spectrum of **2** based on the dipole model only (see main text); (c) Calculation based on the model described in the main text using α_{isoH2} = - 1.265 MHz and $\alpha_{isoH1,3}$ = 0, being in excellent agreement with the calculated α_{iso} values occurring from the A^{C2} and $A^{C1,3}$ using the McConnell model (see main text – Table 2).The dashed-red antidiagonal line marks the ^{1}H Larmor frequency.

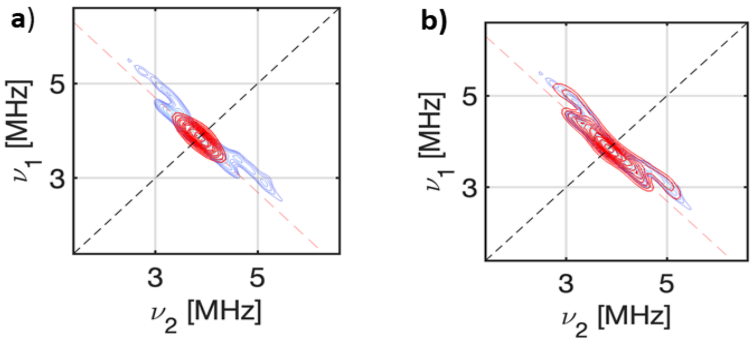

Figure S71. (a) ^{13}C HYSCORE spectrum for **3** (MeTHF) at B_0 = 362.2 mT (xy, **OP3**; Figure S8), T = 10 K and X-band (9.761 GHz), with calculation (red) based on the dipole model only (see main text); (b) Calculation taking into account the point-dipole interactions and the associated spin densities on all the carbon atoms on the Cp^{tt} ring, yielding the following hyperfine constants: $A_{II,\perp}^{C2}$ = 5.9, 0.5 MHz, $A_{II,\perp}^{C1,3}$ = -0.4, -1.5 MHz and $A_{II,\perp}^{C4,5}$ = 0.56, 0.28 MHz. The dashed-red antidiagonal line marks the ^{13}C Larmor frequency.

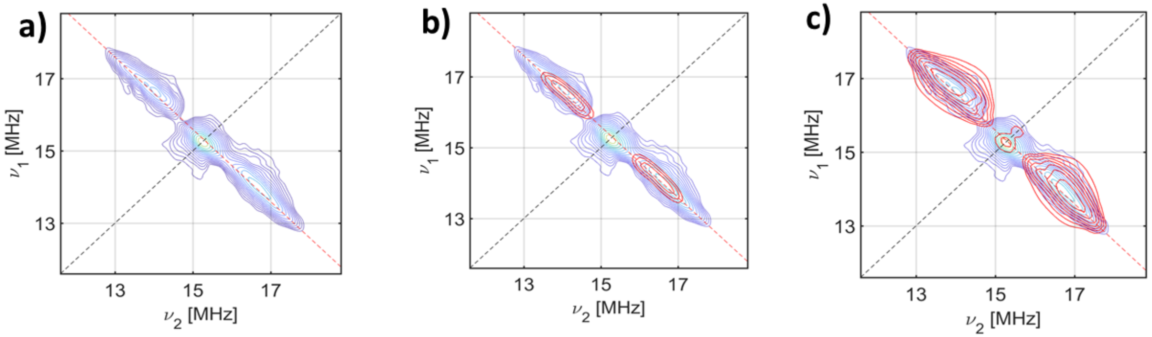

Figure S72. (a) ^{1}H HYSCORE spectrum for **3** (MeTHF) at B_0 = 362.2 mT (xy, **OP3**; Figure S8), T = 20 K and X-band (9.761 GHz); (b) Calculation of the spectrum based on the dipole model only (see main text); (c) Calculation based on the model described in the main text. The dashed-red antidiagonal line marks the ^{1}H Larmor frequency.

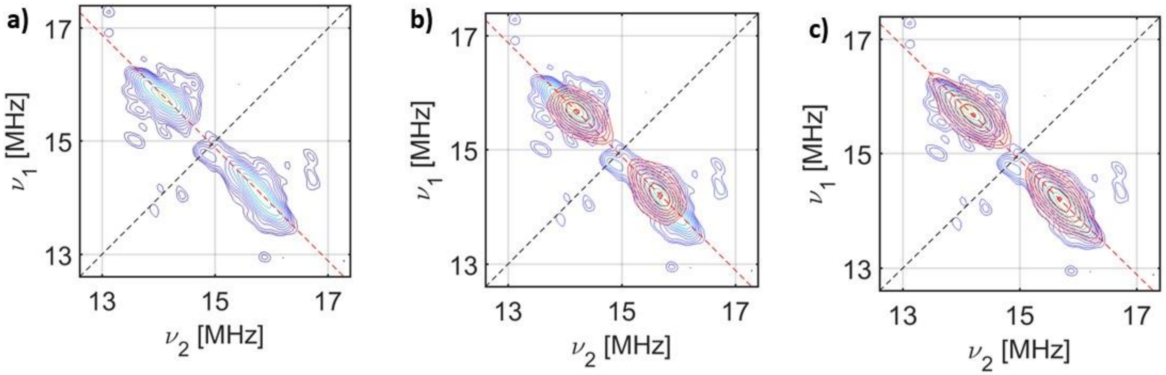

Figure S73. (a) ^{1}H HYSCORE spectrum for **3** (MeTHF) at B_0 = 352.4 mT (z, **OP2**; Figure S8), T = 10 K and X-band (9.76 GHz); (b) Calculation of the ^{1}H HYSCORE spectrum of **3** based on the dipole model only (see main text); (c) Calculation using the same parameters as for Figure S72. The dashed-red antidiagonal line marks the ^{1}H Larmor frequency.

3. **DFT Calculations**

Table S13. DFT-calculated EPR parameters for La(II) in **1-3** using experimental crystal structures.

Compound	$g_{\parallel}$	$g_{\perp}$	g_{iso}	$A_{\parallel}$ (MHz)	$A_{\perp}$ (MHz)	A_{iso} (MHz)	A_{iso} (exp, MHz)
**1**	2.005	1.969	1.981	391	389	390	427
**2**	1.999	1.964	1.976	363	359	360	387
**3**	1.996	1.951	1.966	640	642	641	637

Table S14. DFT-calculated SOMO composition (Löwdin) for La(II) functions and La-based spin density (Löwdin) in **1-3** using experimental crystal structures.

	Compound	La s	La p	La d	La f	La total
**SOMO**	**1**	0.077	0.000	0.594	0.003	-
	**2**	0.078	0.000	0.590	0.000	-
	**3**	0.100	0.001	0.611	0.002	-
**Spin Density**	**1**	0.078	0.010	0.621	0.002	0.712
	**2**	0.080	0.011	0.619	0.001	0.711
	**3**	0.102	0.013	0.647	0.000	0.761

Table S15. DFT-calculated ^{13}C hyperfine coupling parameters for Cp^R rings in **1** using crystal structure, in MHz. Note that average is for all L1-L3.

L1	$A_{\parallel}$	$A_{\perp}$	L2	$A_{\parallel}$	$A_{\perp}$	L3	$A_{\parallel}$	$A_{\perp}$	Avg.	$A_{\parallel}$	$A_{\perp}$
**C1**	0.3	-1.6	**C1**	2.2	-2.6	**C1**	2.5	-2.5	**C1**	1.7	-2.2
**C2**	-2.2	-0.8	**C2**	2.1	-0.5	**C2**	4.2	-0.3	**C2**	1.4	-0.5
**C3**	1.8	-0.5	**C3**	-2.2	-0.6	**C3**	-2.5	-0.7	**C3**	-1.0	-0.6
**C4**	2.2	-1.4	**C4**	0.4	-1.4	**C4**	-0.1	-1.7	**C4**	0.9	-1.5
**C5**	6.3	0.0	**C5**	7.5	0.0	**C5**	6.3	-0.2	**C5**	6.7	-0.1

Table S16. DFT-calculated ^{13}C hyperfine coupling parameters for Cp^R rings in **2** using crystal structure, in MHz. Missing entries for individual atoms indicate hyperfine coupling does not conform well to a simple axial form (i.e. is very rhombic). Note that average is for all L1-L3.

**L1**	$A_\parallel$	$A_\perp$	**L2**	$A_\parallel$	$A_\perp$	**L3**	$A_\parallel$	$A_\perp$	**Avg.**	$A_\parallel$	$A_\perp$
**C1**	-0.5	-2.5	**C1**	2.1	-1.8	**C1**	1.1	-2.9	**C1**	0.9	-2.4
**C2**	9.3	0.5	**C2**	10.1	0.6	**C2**	7.4	0.3	**C2**	8.9	0.5
**C3**	1.9	-2.0	**C3**	0.2	-2.4	**C3**	-3.4	-1.6	**C3**	-0.5	-2.0
**C4**			**C4**			**C4**			**C4**		
**C5**			**C5**			**C5**			**C5**		

Table S17. DFT-calculated ^{13}C hyperfine coupling parameters for Cp^R rings in **3** using crystal structure, in MHz. Missing entries for individual atoms indicate hyperfine coupling does not conform well to a simple axial form (i.e. is very rhombic). Note that average is for all L1-L3.

**L1**	$A_\parallel$	$A_\perp$	**L2**	$A_\parallel$	$A_\perp$	**L3**	$A_\parallel$	$A_\perp$	**Avg.**	$A_\parallel$	$A_\perp$
**C1**	-0.5	-1.5	**C1**	-0.2	-1.2	**C1**	-0.6	-1.5	**C1**	-0.4	-1.4
**C2**	5.4	0.2	**C2**	4.1	0.0	**C2**	4.4	0.1	**C2**	4.7	0.1
**C3**	0.3	-1.6	**C3**	0.0	-1.1	**C3**	0.4	-1.1	**C3**	0.3	-1.2
**C4**			**C4**			**C4**			**C4**		
**C5**			**C5**			**C5**			**C5**		

Table S18. DFT-calculated spin density (Mulliken) for Cp^R rings in **1** using crystal structure.

**L1**		**L2**		**L3**	
**C1**	-0.0226	**C1**	-0.0249	**C1**	-0.0300
**C2**	-0.0259	**C2**	0.0089	**C2**	0.0308
**C3**	0.0195	**C3**	-0.0162	**C3**	-0.0200
**C4**	-0.0135	**C4**	-0.0200	**C4**	-0.0188
**C5**	0.0509	**C5**	0.0510	**C5**	0.0436

Table S19. DFT-calculated spin density (Mulliken) for Cp^R rings in **2** using crystal structure.

**L1**		**L2**		**L3**	
**C1**	-0.0352	**C1**	-0.0289	**C1**	-0.0470
**C2**	0.0701	**C2**	0.0732	**C2**	0.0594
**C3**	-0.0439	**C3**	-0.0398	**C3**	-0.0307
**C4**	0.0118	**C4**	-0.0137	**C4**	-0.0281
**C5**	-0.0232	**C5**	-0.0029	**C5**	0.0250

Table S20. DFT-calculated spin density (Mulliken) for Cp^R rings in **3** using crystal structure.

**L1**		**L2**		**L3**	
**C1**	-0.0328	**C1**	-0.0180	**C1**	-0.0323
**C2**	0.0472	**C2**	0.0446	**C2**	0.0426
**C3**	-0.0170	**C3**	-0.0420	**C3**	-0.0254
**C4**	-0.0149	**C4**	0.0026	**C4**	-0.0108
**C5**	-0.0052	**C5**	-0.0192	**C5**	-0.0094

Table S21. DFT-calculated ^{13}C hyperfine coupling parameters for Cp^R rings in **1** using optimised structure, in MHz. Missing entries for individual atoms indicate hyperfine coupling does not conform well to a simple axial form (i.e. is very rhombic). Note that average here is only for L2 and L3, while L1 is distinct.

**L1**	$A_{\parallel}$	$A_{\perp}$	**L2**	$A_{\parallel}$	$A_{\perp}$	**L3**	$A_{\parallel}$	$A_{\perp}$	**Avg.**	$A_{\parallel}$	$A_{\perp}$
**C1**	-0.2	-1.7	**C1**	2.6	-1.7	**C1**	2.2	-1.7	**C1**	2.4	-1.7
**C2**	-1.6	-0.4	**C2**	5.0	0.5	**C2**	3.0	0.0	**C2**	4.0	0.2
**C3**	2.1	-0.2	**C3**			**C3**	-2.1	-0.7	**C3**		
**C4**	5.4	1.6	**C4**			**C4**	2.0	0.0	**C4**		
**C5**	5.9	0.5	**C5**	5.4	0.6	**C5**	7.8	0.9	**C5**	6.6	0.7

Table S22. DFT-calculated ^{13}C hyperfine coupling parameters for Cp^R rings in **2** using optimised structure, in MHz. Missing entries for individual atoms indicate hyperfine coupling does not conform well to a simple axial form (i.e. is very rhombic). Note that average is for all L1-L3.

**L1**	$A_{\parallel}$	$A_{\perp}$	**L2**	$A_{\parallel}$	$A_{\perp}$	**L3**	$A_{\parallel}$	$A_{\perp}$	**Avg.**	$A_{\parallel}$	$A_{\perp}$
**C1**	0.0	-2.6	**C1**	0.1	-2.6	**C1**	0.0	-2.5	**C1**	0.0	-2.5
**C2**	9.9	0.9	**C2**	9.8	0.9	**C2**	9.7	0.9	**C2**	9.8	0.9
**C3**	0.1	-2.6	**C3**	0.0	-2.6	**C3**	0.0	-2.5	**C3**	0.0	-2.6
**C4**			**C4**			**C4**			**C4**		
**C5**			**C5**			**C5**			**C5**		

Table S23. DFT-calculated ^{13}C hyperfine coupling parameters for Cp^R rings in **3** using optimised structure, in MHz. Missing entries for individual atoms indicate hyperfine coupling does not conform well to a simple axial form (i.e. is very rhombic). Note that average is for all L1-L3.

**L1**	$A_{\parallel}$	$A_{\perp}$	**L2**	$A_{\parallel}$	$A_{\perp}$	**L3**	$A_{\parallel}$	$A_{\perp}$	**Avg.**	$A_{\parallel}$	$A_{\perp}$
**C1**	-0.4	-1.2	**C1**	-0.3	-1.3	**C1**	-0.3	-1.2	**C1**	-0.3	-1.2
**C2**	4.6	0.5	**C2**	4.7	0.5	**C2**	4.6	0.5	**C2**	4.6	0.5
**C3**	-0.3	-1.2	**C3**	-0.4	-1.3	**C3**	-0.4	-1.3	**C3**	-0.3	-1.2
**C4**			**C4**			**C4**			**C4**		
**C5**			**C5**			**C5**			**C5**		

Table S24. DFT-calculated spin density (Mulliken) for Cp^R rings in **1** using optimised structure.

**L1**		**L2**		**L3**	
**C1**	-0.0211	**C1**	-0.0281	**C1**	-0.0308
**C2**	-0.0227	**C2**	0.0255	**C2**	0.0125
**C3**	0.0143	**C3**	-0.0190	**C3**	-0.0189
**C4**	-0.0033	**C4**	-0.0177	**C4**	-0.0125
**C5**	0.0342	**C5**	0.0287	**C5**	0.0455

Table S25. DFT-calculated spin density (Mulliken) for Cp^R rings in **2** using optimised structure.

**L1**		**L2**		**L3**	
**C1**	-0.0399	**C1**	-0.0401	**C1**	-0.0405
**C2**	0.0733	**C2**	0.0735	**C2**	0.0732
**C3**	-0.0407	**C3**	-0.0393	**C3**	-0.0398
**C4**	-0.0044	**C4**	-0.0057	**C4**	-0.0064
**C5**	-0.0065	**C5**	-0.0071	**C5**	-0.0059

Table S26. DFT-calculated spin density (Mulliken) for Cp^R rings in **3** using optimised structure.

**L1**		**L2**		**L3**	
**C1**	-0.0329	**C1**	-0.0327	**C1**	-0.0297
**C2**	0.0532	**C2**	0.0524	**C2**	0.0494
**C3**	-0.0330	**C3**	-0.0341	**C3**	-0.0357
**C4**	-0.0110	**C4**	-0.0111	**C4**	-0.0096
**C5**	-0.0083	**C5**	-0.0075	**C5**	-0.0097

Table S27. DFT-calculated EPR parameters for La(II) in **1-3** using optimised structures.

**Compound**	$g_{\parallel}$	$g_{\perp}$	g_{iso}	$A_{\parallel}$ **(MHz)**	$A_{\perp}$ **(MHz)**	A_{iso} **(MHz)**	A_{iso} **(exp, MHz)**
**1**	2.001	1.968	1.979	419	417	417	427
**2**	1.999	1.974	1.982	352	347	349	387
**3**	2.004	1.963	1.977	621	620	620	637

Table S28. DFT-calculated La-based spin density (Löwdin) in **1-3** using optimised structures.

	**Compound**	**La s**	**La p**	**La d**	**La f**	**La total**
**Spin Density**	**1**	0.08	0.01	0.62	0.00	0.71
	**2**	0.08	0.01	0.62	0.00	0.71
	**3**	0.10	0.01	0.65	0.00	0.76

4. References

1 M. E. Fieser, M. R. MacDonald, B. T. Krull, J. E. Bates, J. W. Ziller, F. Furche and W. J. Evans. Structural, Spectroscopic, and Theoretical Comparison of Traditional vs Recently Discovered Ln^{2+} Ions in the [K(2.2.2-cryptand)][($C_5H_4SiMe_3$)$_3$Ln] Complexes: The Variable Nature of Dy^{2+} and Nd^{2+}. *J. Am. Chem. Soc.*, **2015**, 137, 369-382.

2. P. B. Hitchcock, M. F. Lappert, L. Maron and A. V. Protchenko. Lanthanum Does Form Stable Molecular Compounds in the +2 Oxidation State. *Angew. Chem. Int. Ed.*, **2008**, 47, 1488-1491.

3. J. Liu, PhD thesis, University of Manchester, 2019.

4. A. Schweiger and J. Jeschke. Principles of Pulse Electron Paramagnetic Resonance. *Oxford University Press*, Oxford, **2001**.

5. S. Stoll and A. Schweiger. Easyspin, a comprehensive software package for spectral simulation and analysis in EPR. *J. Magn. Reson.*, **2006**, 178, 42-55.

6. S. Stoll and R. D. Britt. General and efficient simulation of pulse EPR spectra. *Phys. Chem. Chem. Phys.*, 2009, 11, 6614-6625.
